# Supplementary material for: The sequences of 150,119 genomes in the UK Biobank
Source: Nature. 2022 Jul 20;607(7920):732–40. doi: 10.1038/s41586-022-04965-x (PMC9329122; doi:10.1038/s41586-022-04965-x)
Supplement: Supplementary file 1 — This file contains Supplementary Figs. 1–22, Supplementary Tables 1–25, Supplementary Notes 1–23 and Supplementary References—see contents page for details. [file 41586_2022_4965_MOESM1_ESM.pdf]

nature portfolio

https://doi.org/10.1038/s41586-022-04965-x

Supplementary information

The sequences of 150,119 genomes in the UK Biobank

In the format provided by the authors and unedited

Supplementary material:

The sequences of 150,119 genomes in the UK biobank

Supplementary Fig. 1 Histogram of average sequence coverage per sample in the 150,119 WGS samples. 4

Supplementary Fig. 2 Sensitivity and precision for GATK and GraphTyper callsets in 500 regions benchmarking dataset across the seven Genome in a bottle (GIAB) v3.3.2 truth sets. 5

Supplementary Fig. 3 Fraction of rare variants (FRV) as a function of the definition of “rare”. 6

Supplementary Fig. 4 Imputation and phasing accuracy across variant datasets in the three populations. 7

Supplementary Fig. 5 Process outline for UKB sequencing pipeline at deCODE genetics. 8

Supplementary Fig. 6 Pipeline for processing of sequence data at deCODE genetics. 9

Supplementary Fig. 7 Logic used to compute PASS/FAIL for a WGS cram file. 10

Supplementary Fig. 8 Average sequence coverage per base pair across the genome. 11

Supplementary Fig. 9 Number of variants per region in the 500 regions test set for the GATK and GraphTyper callsets. 12

Supplementary Fig. 10 Distribution of indel sizes in GATK and GraphTyper callsets. 13

Supplementary Fig. 11 VAF and mutation classes in SNP and indel call sets. 14

Supplementary Fig. 12 Fraction of variants by mutation type in the GATK, GraphTyper and GraphTyper HQ sets. 15

Supplementary Fig. 13 Imputation accuracy for variants with AAscore > 0.9 in the three populations. 16

Supplementary Fig. 14 Manhattan plots, quantile-quantile (QQ) plots and histograms of inverse-normal transformed values after adjustment for covariates age, sex and 40 principal components, when applicable, for quantitative traits with significant results reported in this manuscript. 17

Supplementary Fig. 15 Manhattan plots and quantile-quantile (QQ) plots for case-control phenotypes with significant results reported in this manuscript. 30

Supplementary Fig. 16 Locus plots. 33

Supplementary Fig. 17 UMAP and ethnicity. 34

Supplementary Fig. 18 Cohort ADMIXTURE summaries. 35

Supplementary Fig. 19 UMAP ADMIXTURE. 36

Supplementary Fig. 20 The first six principal components of the XBI cohort. 37

Supplementary Fig. 21 The first six principal components of the XAF cohort. 38

Supplementary Fig. 22 The first six principal components of the XSA cohort. 39

Supplementary Table 1 Genome in a bottle (GIAB) v3.3.2 truth set comparison of GATK and GraphTyper in 500 random regions. 40

Supplementary Table 2 Genotype consistency 41

Supplementary Table 3 Analysis of variant transmission of related samples in the 500 randomly selected 50kb test regions. 42

Supplementary Table 4 Comparison of imputation of variants from the GATK and GraphTyper call sets on chr22 10-11Mb in the XBI dataset. 43

Supplementary Table 5 Comparison of SNP and Indel call sets to WES. 44

Supplementary Table 6 Mutation saturation, results presented for autosomes and chrX separately. 45

Supplementary Table 7 SNP and Indel call set summary 46

Supplementary Table 8 Regression of average DR overlapping gene exons on annotations from Gene discovery informatics toolkit33. 47

Supplementary Table 9 a) Pearson correlation coefficient and b) r2 between DR score and measures of sequence constraint and functional impact, computed over all autosomal chromosomes. 48

Supplementary Table 10 Number of individuals in the three cohorts described in this study. 49

Supplementary Table 11 Imputation and phasing accuracy as a function of frequency within each cohort. 50

Supplementary Table 12 Number of markers that impute (Imp Info > .8) in 500k set of UKB using the imputation panel presented here (150k WGS) and an imputation by Bycroft et al.5. 51

Supplementary Table 13 Association of number of repeat copies of microsatellite in 3' UTR in DMPK with myotonic dystrophy. 52

Supplementary Table 14 Information on genes presented. 53

Supplementary Table 15 Phenotypes used in this study, their field in the UKB data showcase and adjustments performed prior to association analysis 54

Supplementary Table 16 QA/QC metrics derived from the files delivered to the UKB. 55

Supplementary Table 17 Metrics collected for each lane by bamqc_summary. 56

Supplementary Table 18 Results for 500 random test regions. 57

Supplementary Table 19 Number of common variants (frequency > .1%) that showed significant association with sequencing center in the 500 random regions test set. 58

Supplementary Table 20 Number of common variants (frequency > 0.1%) that show significant association to sequencing center, indicating batch effects. 59

Supplementary Table 21 Three-way comparison between the GraphTyperHQ, GATK and WES200k59 call analyzed inside WES capture regions within the set of 109,618 individuals present in both the WES200k call set an our set of 150,119 individuals. 60

Supplementary Table 22 Batch effects for sequencing center in the raw genotype calls. 61

Supplementary Table 23 Batch effects for sequencing center in the imputed genotype calls. 62
Supplementary Table 24 Correction factors and inflation metrics from phenotypes used in this study. 63
Supplementary Table 25 R2 between raw genotypes and imputed markers in the XBI cohort. 64

Supplementary Note 1: WGS data quality specification. 65
Supplementary Note 2: Whole genome sequencing 66
Supplementary Note 3: Sequence processing pipeline 66
Supplementary Note 4: Sequence coverage 68
Supplementary Note 5: SNP and indel calling with Calling with GATK 69
Supplementary Note 6: Evaluation of SNP and indel callers across 500 random regions 70
Supplementary Note 7: Comparison of final GraphTyper and GATK call sets. 73
Supplementary Note 8: Batch effects in final dataset 74
Supplementary Note 9: Overlap with UKBB WES SNPs 75
Supplementary Note 10: Microsatellite calling with popSTR 76
Supplementary Note 11: Imputation results 78
Supplementary Note 12: Genome annotation 78
Supplementary Note 13: WGS individuals carrying actionable genotypes meeting ACMG criteria 79
Supplementary Note 14: Genotype count of rare LoF variants 79
Supplementary Note 15: GWAS enrichment analysis 79
Supplementary Note 16: Overlap with ENCODE regions 80
Supplementary Note 17: RNA sequence data 80
Supplementary Note 18: Computing principal components within cohorts 80
Supplementary Note 19: Inbreeding 81
Supplementary Note 20: IBD segment computation 82
Supplementary Note 21: ADMIXTURE 82
Supplementary Note 22: Birthplace data 82
Supplementary Note 23: Websites 82
Supplentary References 84

Supplementary Figures

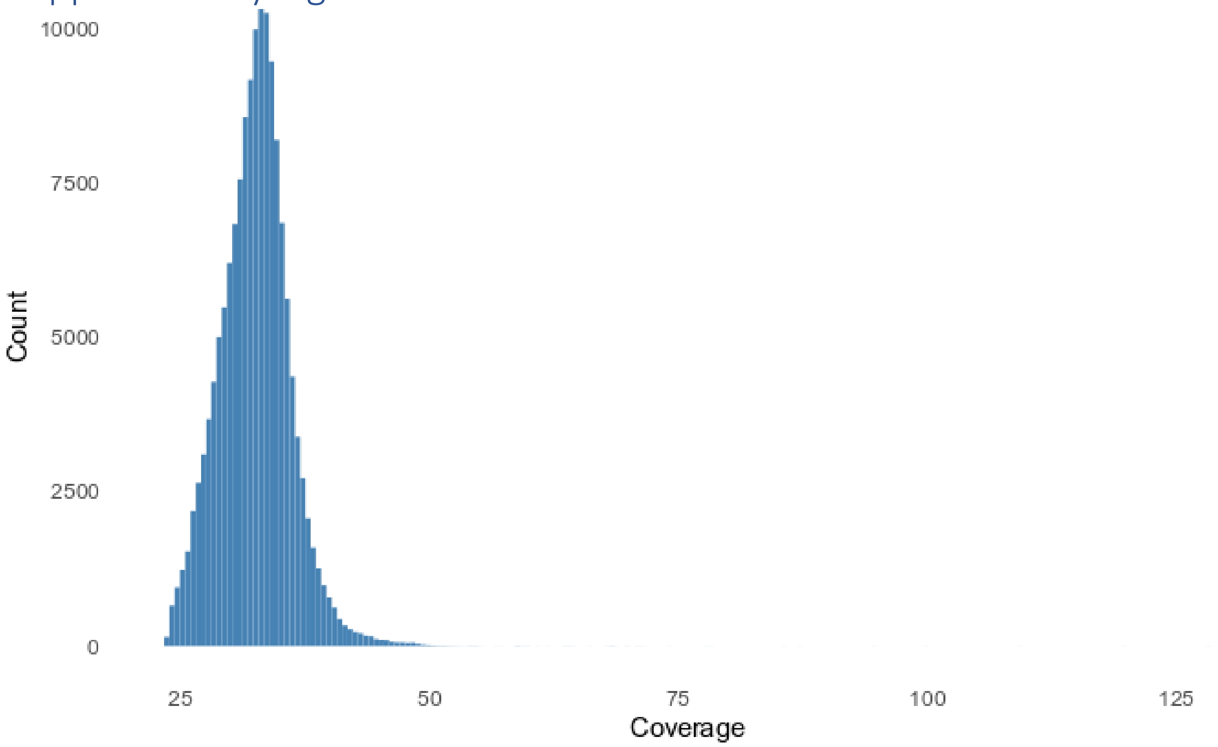

Supplementary Fig. 1 Histogram of average sequence coverage per sample in the 150,119 WGS samples.

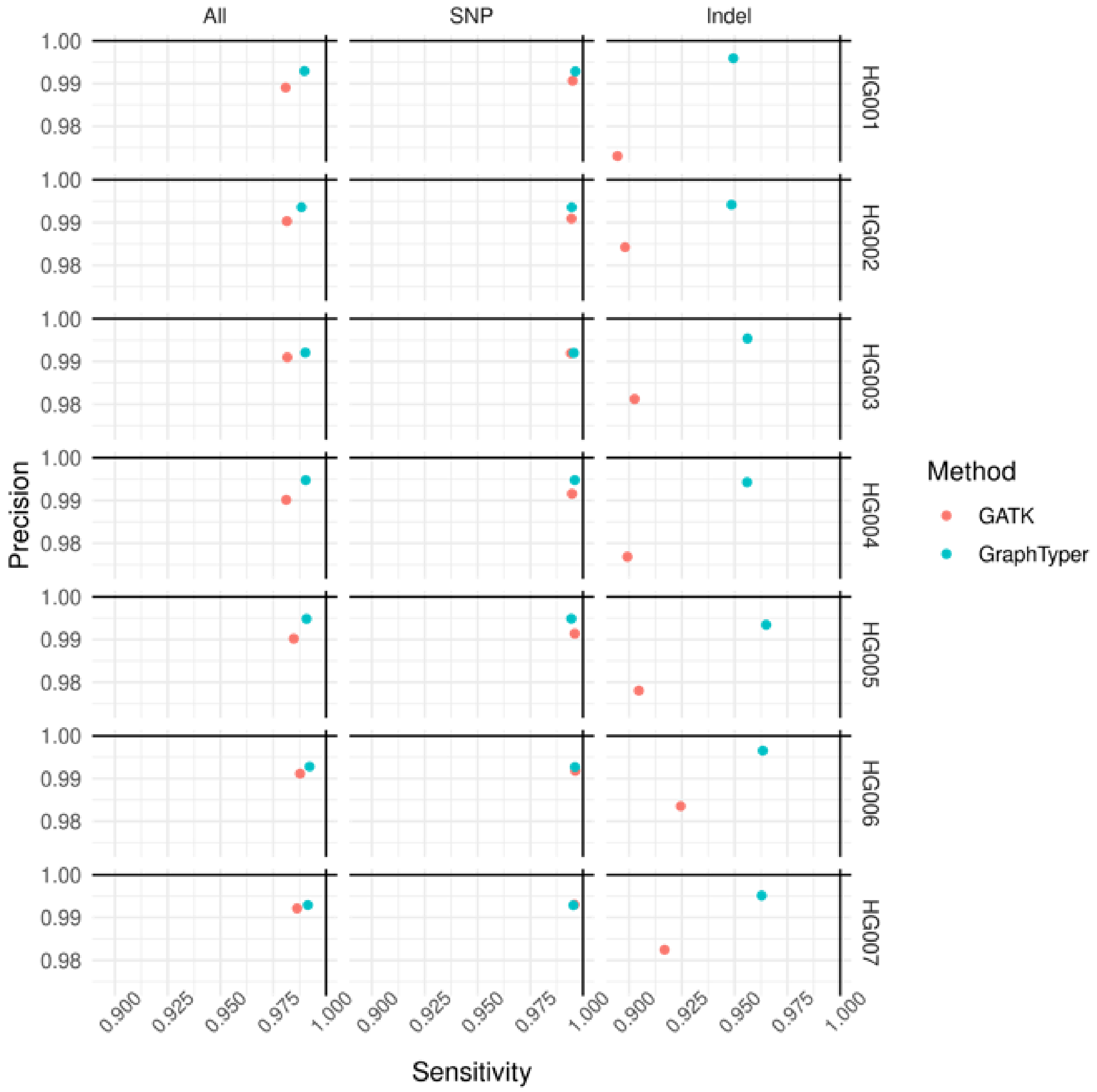

Supplementary Fig. 2 Sensitivity and precision for GATK and GraphTyper callsets in 500 regions benchmarking dataset across the seven Genome in a bottle (GIAB) v3.3.2 truth sets.

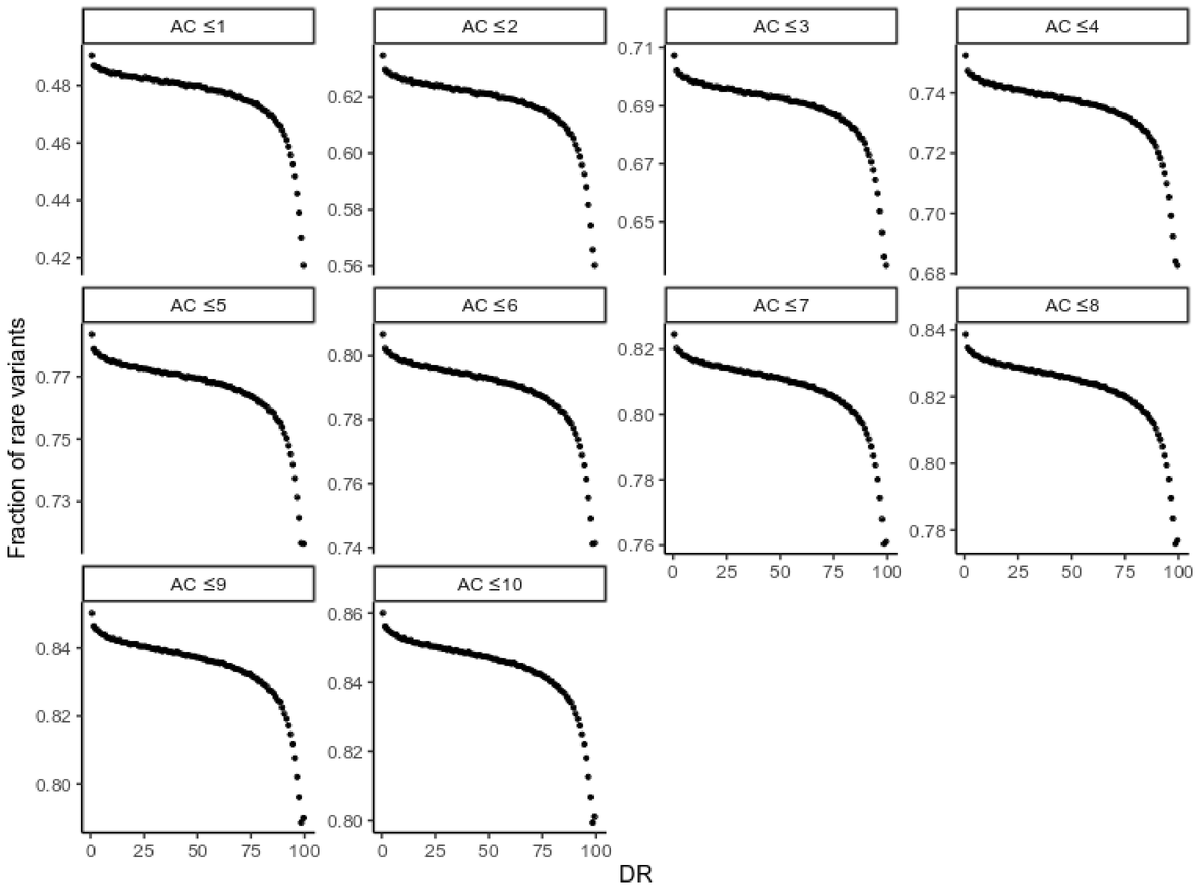

Supplementary Fig. 3 Fraction of rare variants (FRV) as a function of the definition of “rare”. We vary the allele count cutoff from at most 1 to at most 10 carriers. Note that homozygous carriers have an allele count of 2.

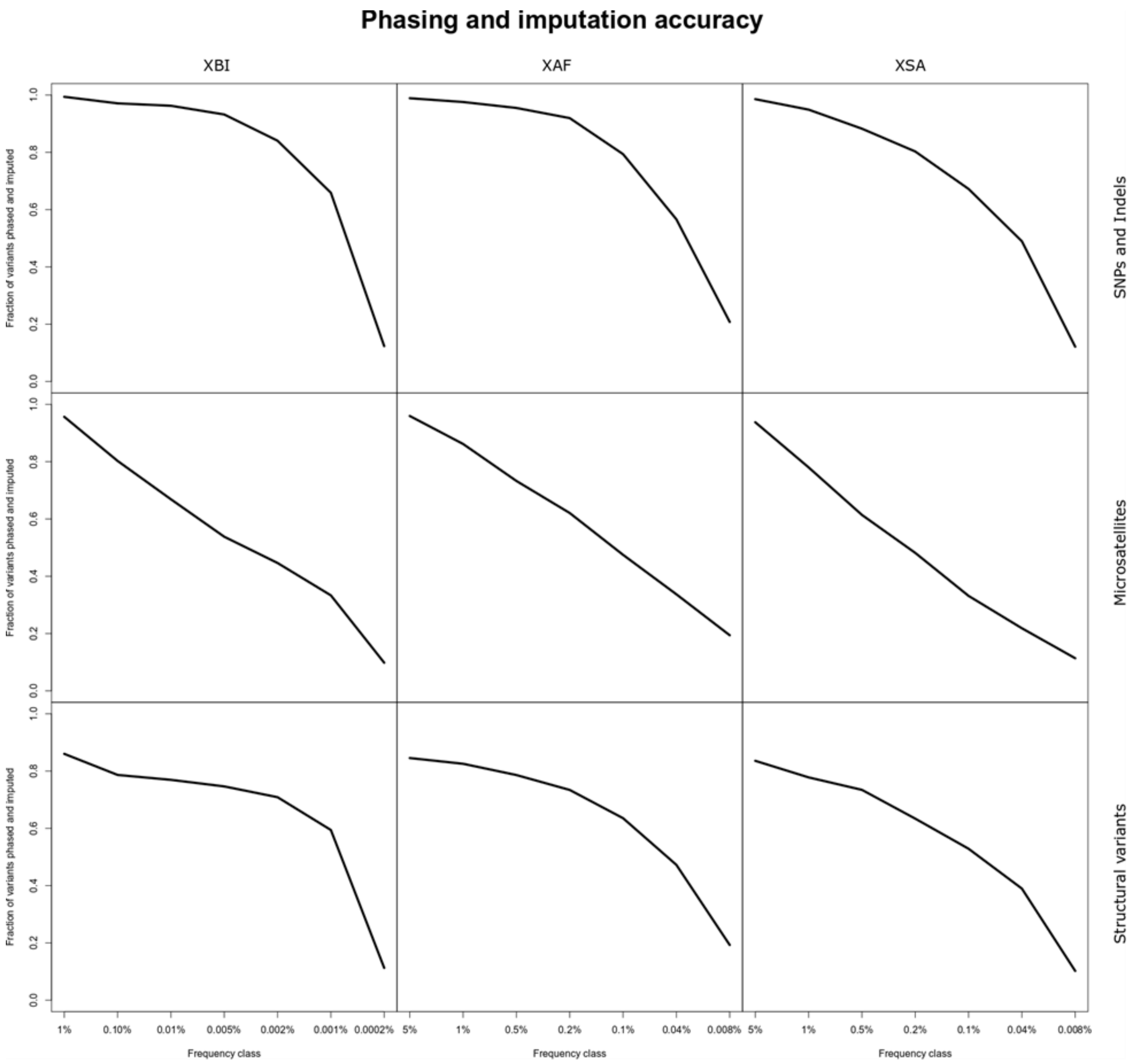

Supplementary Fig. 4 Imputation and phasing accuracy across variant datasets in the three populations.

A variant is considered imputed if Leave one out r2 (L1or2) of phasing was greater than 0.5 and imputation information was greater than 0.8. x-axis splits variants into frequency classes based on the frequency in each cohort.

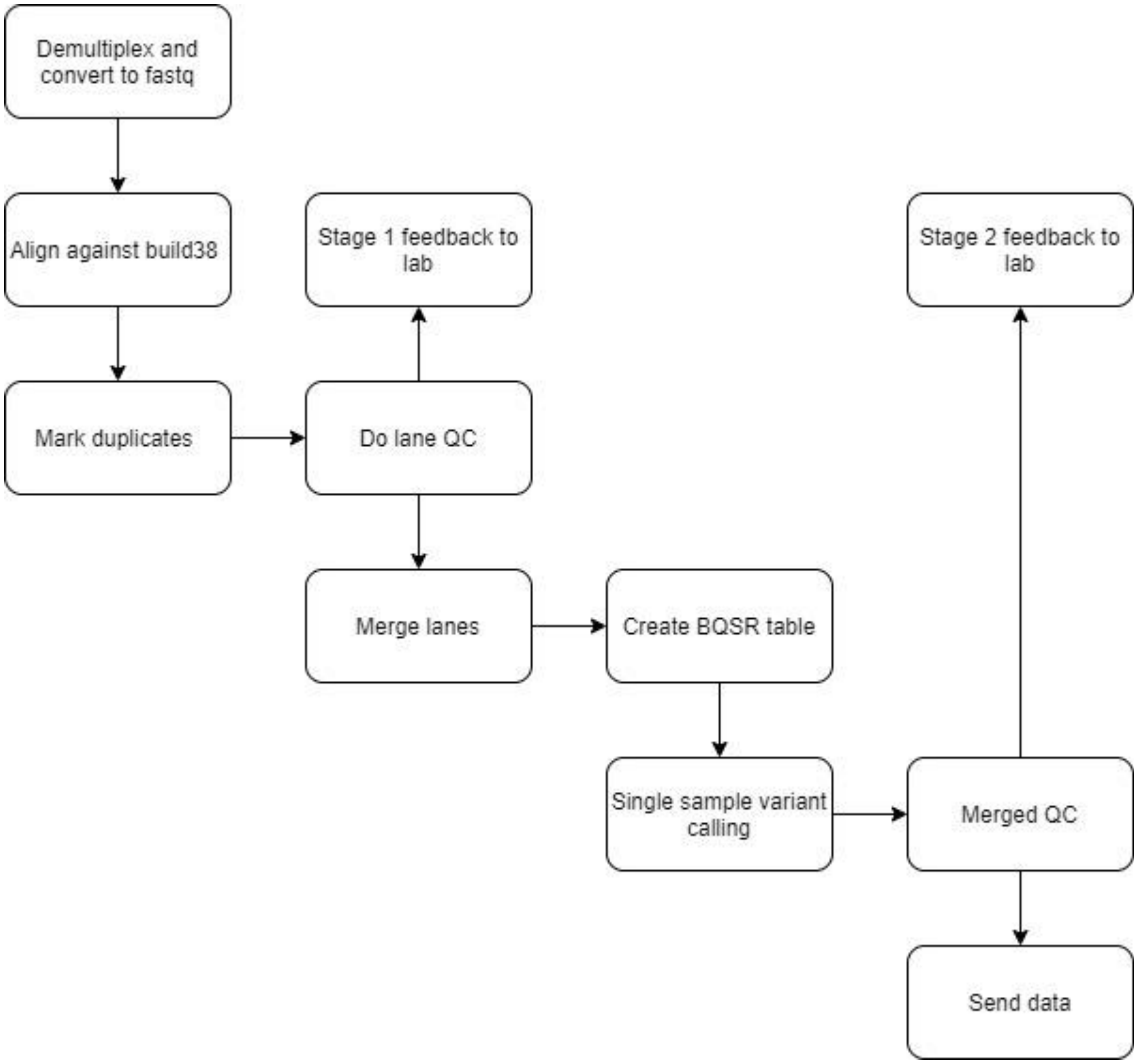

Supplementary Fig. 5 Process outline for UKB sequencing pipeline at deCODE genetics.

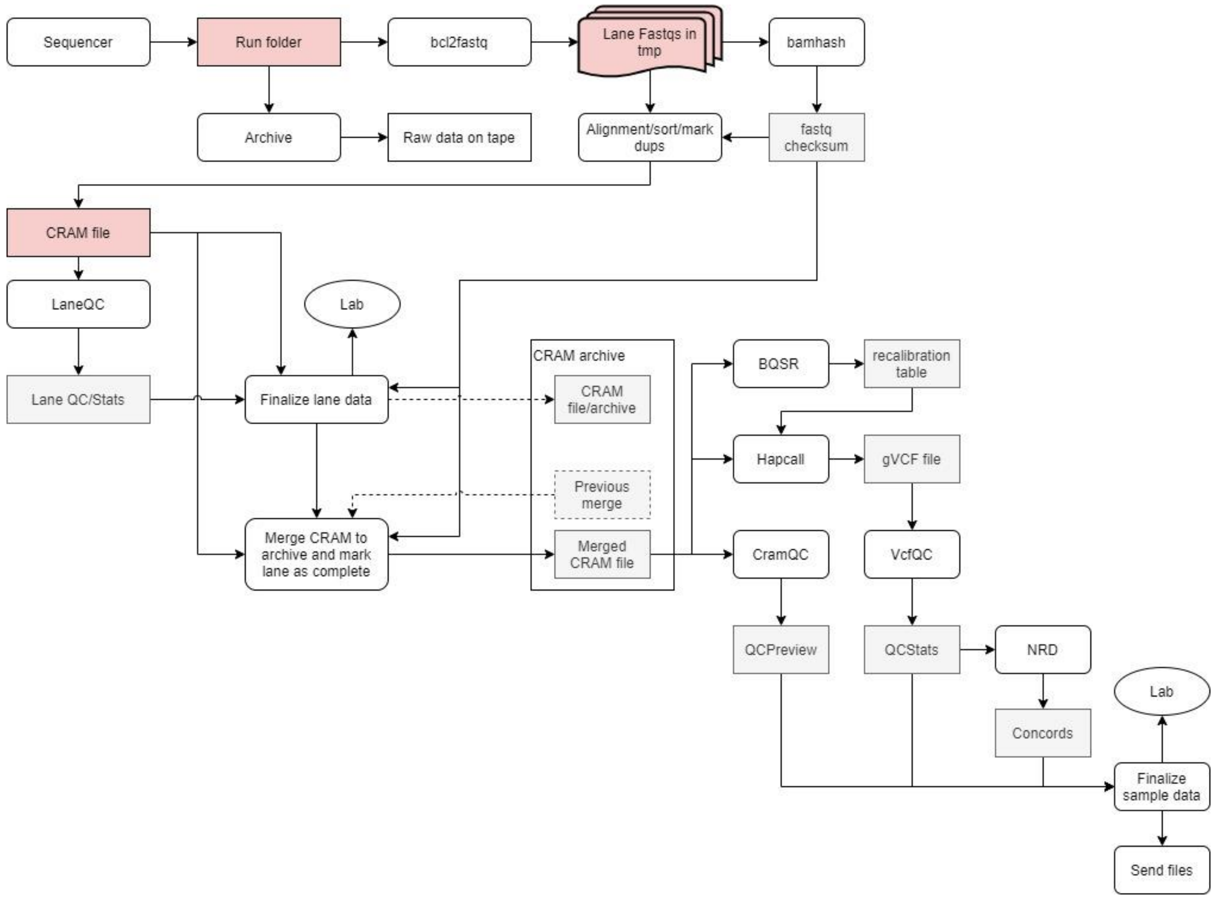

Supplementary Fig. 6 Pipeline for processing of sequence data at deCODE genetics.

```
QC_VERDICT = 'PASS'

    if freemix_percentage >= 1.0:
        QC_VERDICT = 'REVIEW'

    if coverage < 26:
        QC_VERDICT = 'REVIEW'

    if freemix_percentage >= 5.0:
        QC_VERDICT = 'FAIL'

    if prc_proper_pairs < 95.0:
        QC_VERDICT = 'FAIL'

    if prc_auto_ge_15x < 95.0:
        QC_VERDICT = 'FAIL'

    if discordance_prc is not -1 and discordance_prc >= 2.0:
        QC_VERDICT = 'FAIL'
```

Supplementary Fig. 7 Logic used to compute PASS/FAIL for a WGS cram file.

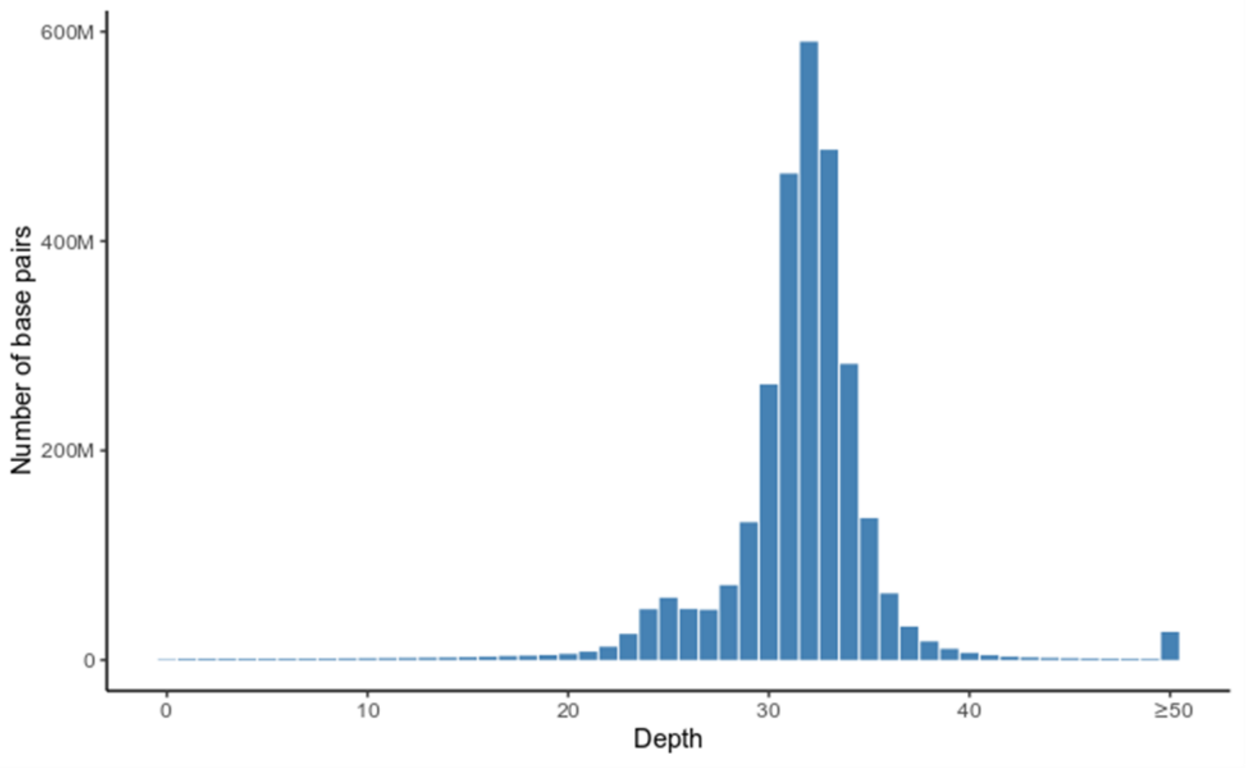

Supplementary Fig. 8 Average sequence coverage per base pair across the genome. The average coverage is computed from 1,000 randomly selected samples.

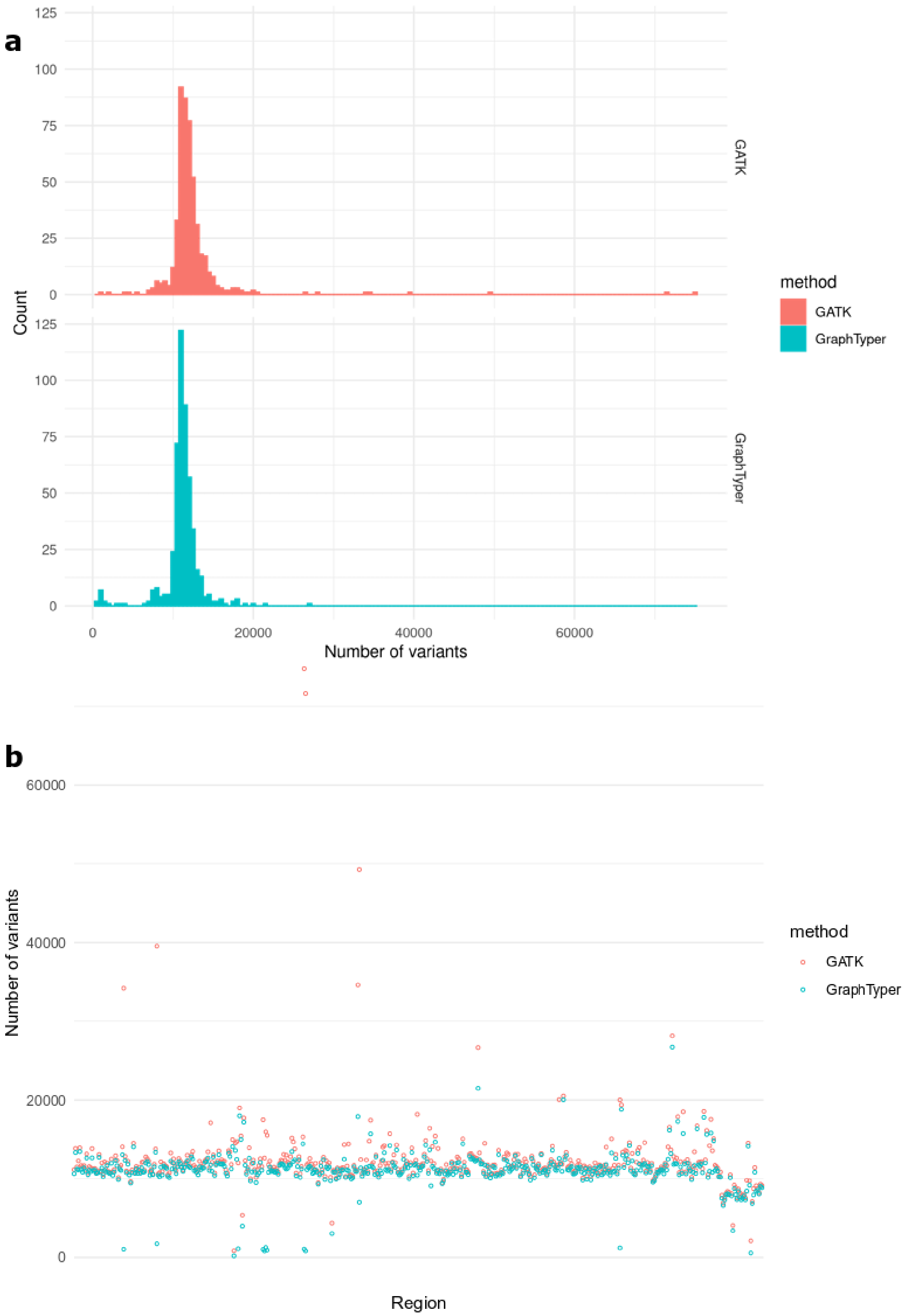

Supplementary Fig. 9 Number of variants per region in the 500 regions test set for the GATK and GraphTyper callsets.
Presented as a histogram a) and ordered by region b).

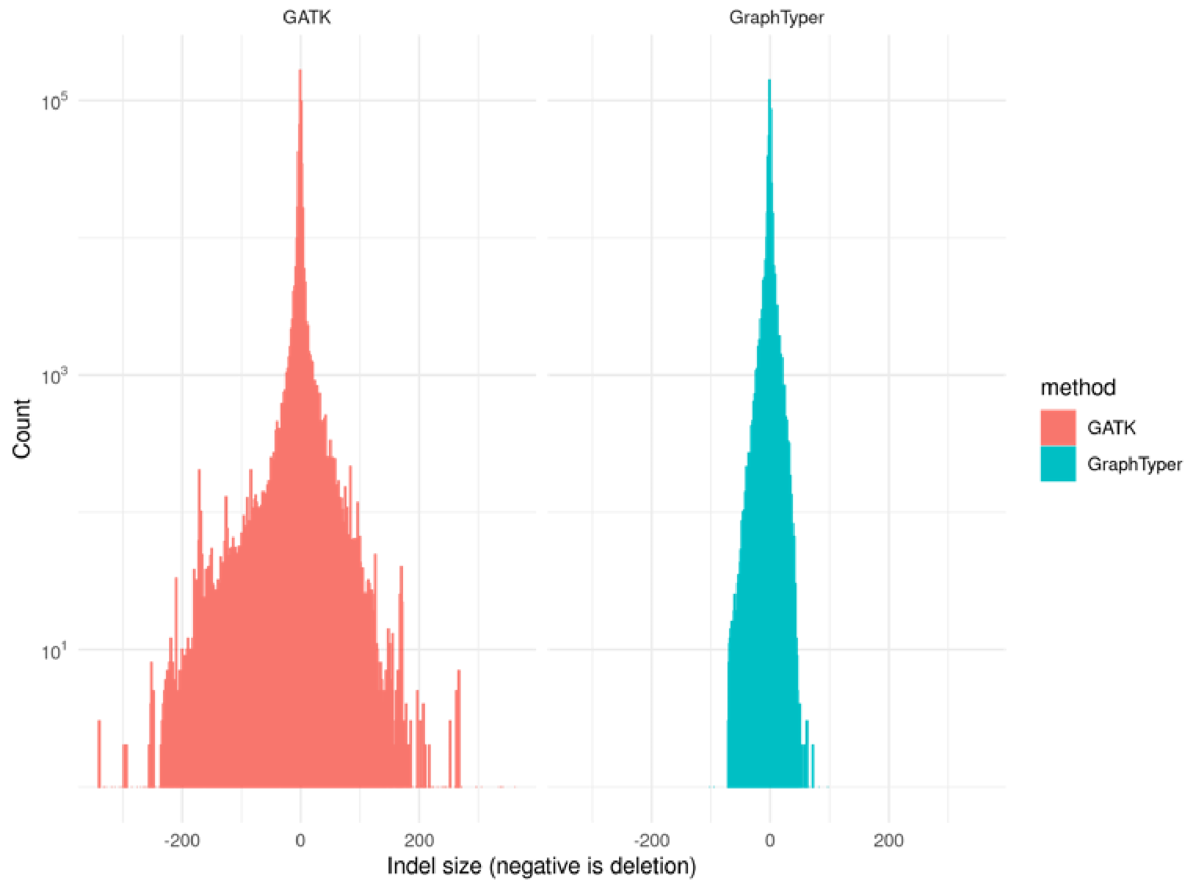

Supplementary Fig. 10 Distribution of indel sizes in GATK and GraphTyper callsets. Negative size indicates a deletion.

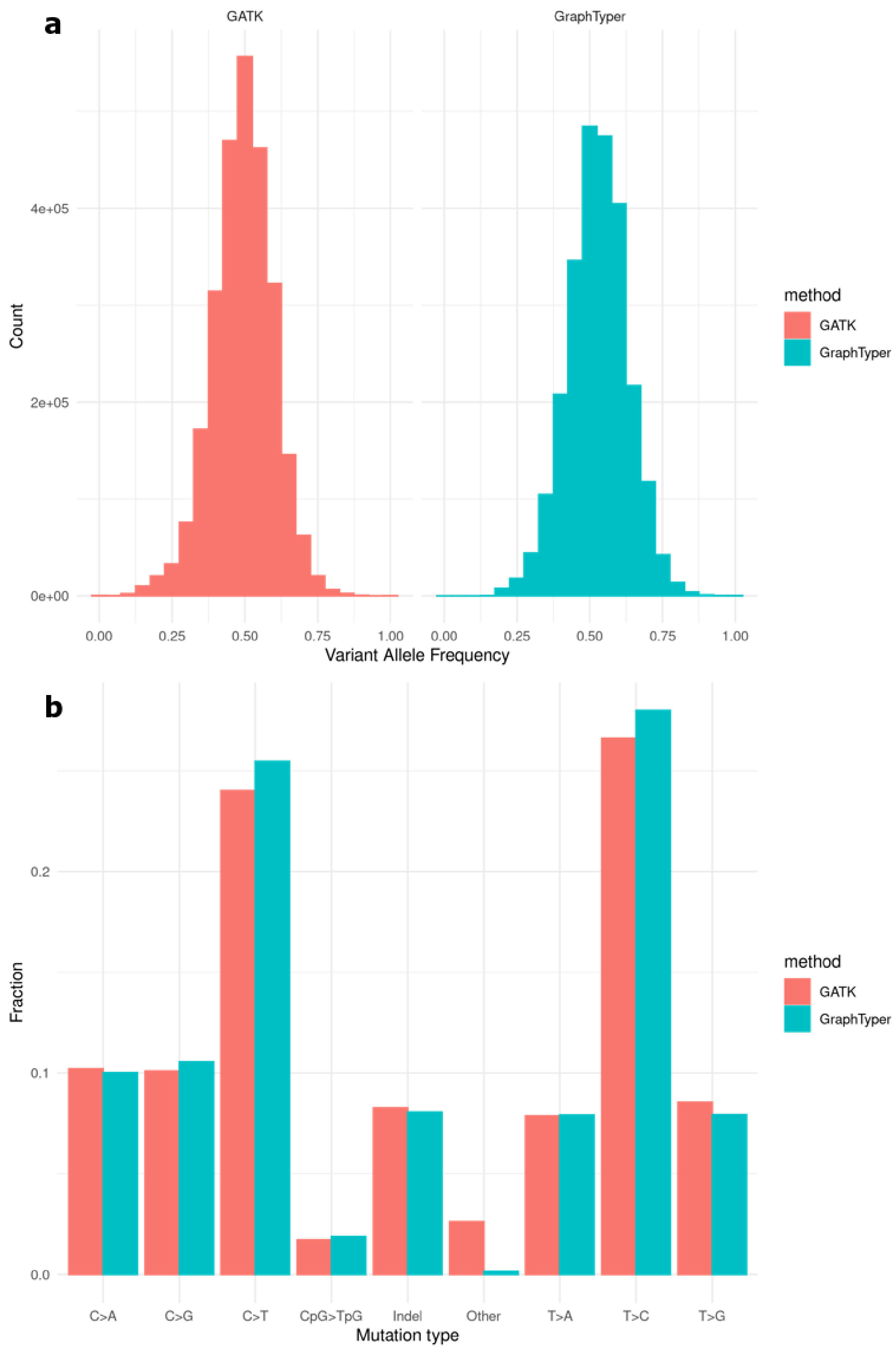

Supplementary Fig. 11 VAF and mutation classes in SNP and indel call sets.
a) Variant allele frequencies (VAF) of singletons. b) Mutation classes of singletons. Results are for the GATK and GraphTyper callsets on 500 randomly selected regions.

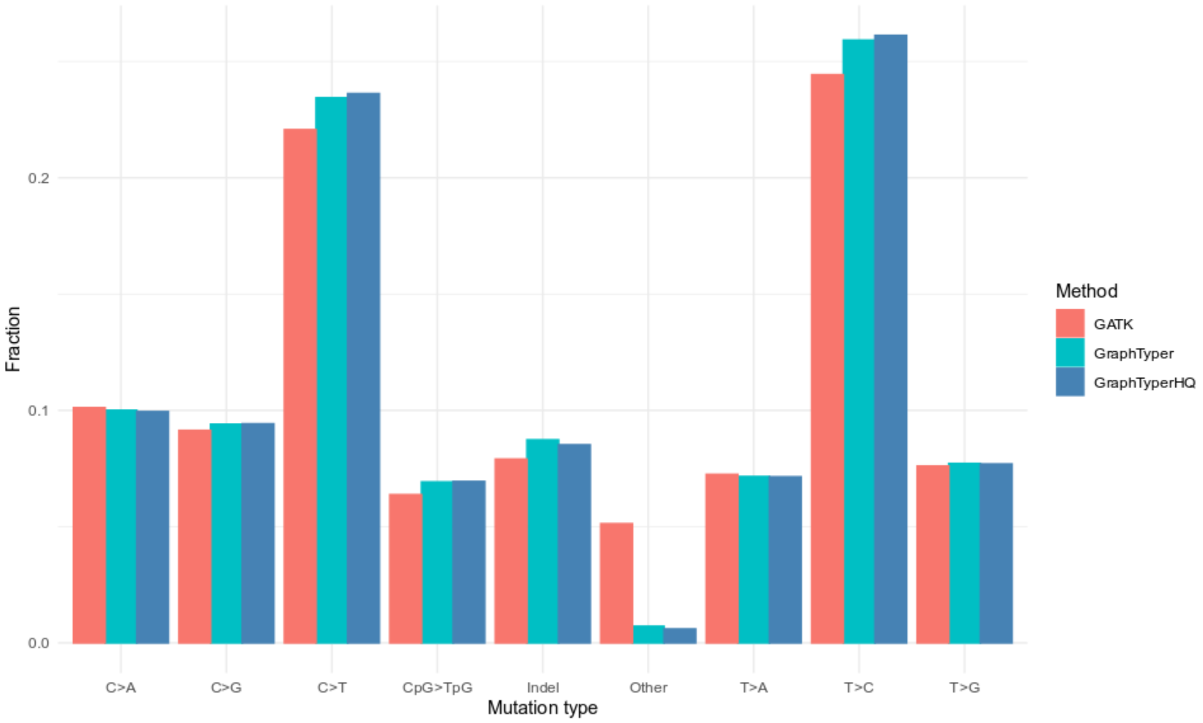

Supplementary Fig. 12 Fraction of variants by mutation type in the GATK, GraphTyper and GraphTyper HQ sets.

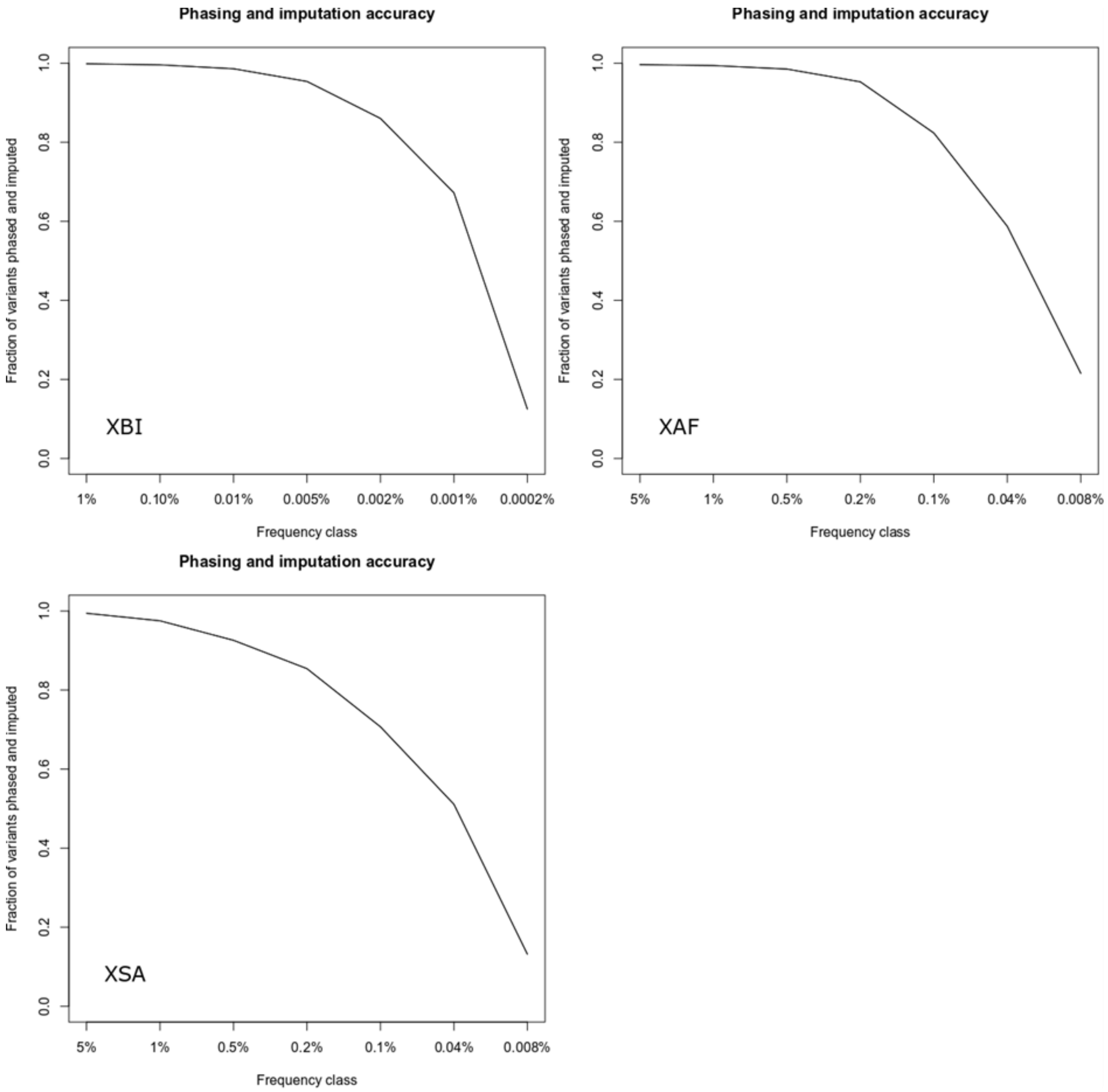

Supplementary Fig. 13 Imputation accuracy for variants with AAscore > 0.9 in the three populations.

Top left: XBI, Top Right: XAF, Bottom: XSA. A variant was considered imputed if Leave one out r2 of phasing was greater than 0.5 and imputation information was greater than 0.8. x-axis splits variants into frequency classes based on the number of carriers in the sequence dataset, with the number representing the minimum number of carriers in the frequency class. Variants are split by variant type.

Supplementary Fig. 14 Manhattan plots, quantile-quantile (QQ) plots and histograms of inverse-normal transformed values after adjustment for covariates age, sex and 40 principal components, when applicable, for quantitative traits with significant results reported in this manuscript.
For Manhattan plots, the x-axis represents chromosome locations and the y-axis shows the –log10 significance levels of the associations. For QQ plots, the inflation (λ) is shown in the title of each graph, for all variants and for rare variants only (λ_maf<0.01). For the histograms, the x-axis shows the value range of the inverse-normal transformed points and the y-axis shows the count of individuals within value ranges. P-values are computed using a two-sided χ^2-test.

a) Total cholesterol, structural variant analysis, European ancestry (N=412,119)

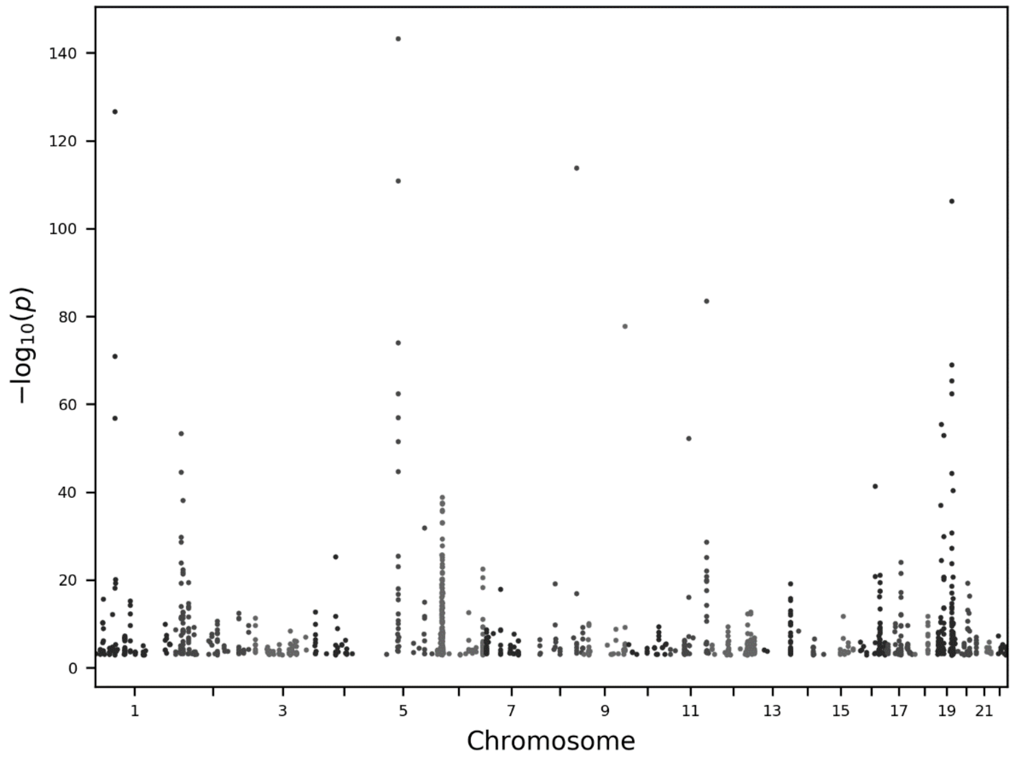

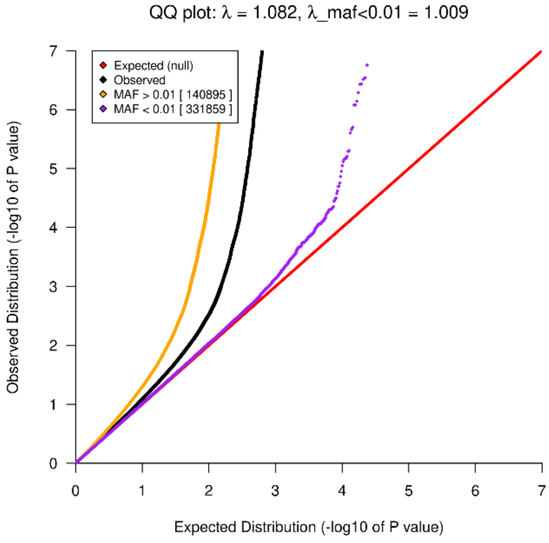

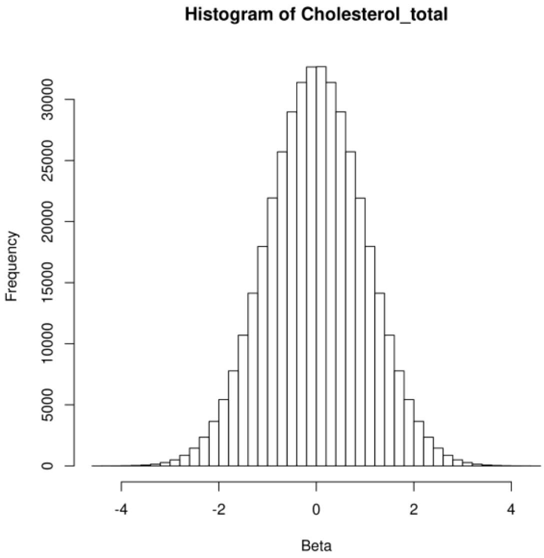

b) Calcium levels, structural variant analysis, European ancestry (N=378,246)

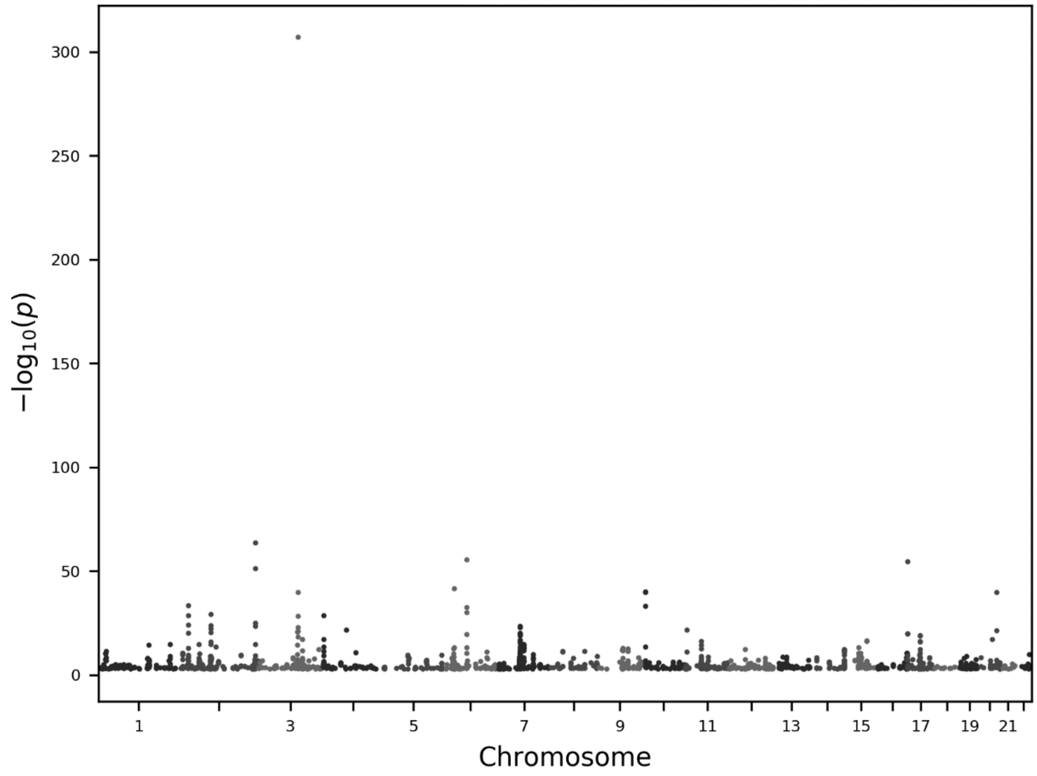

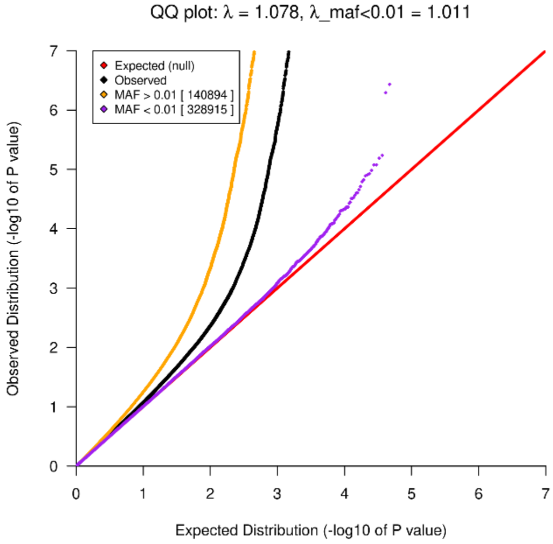

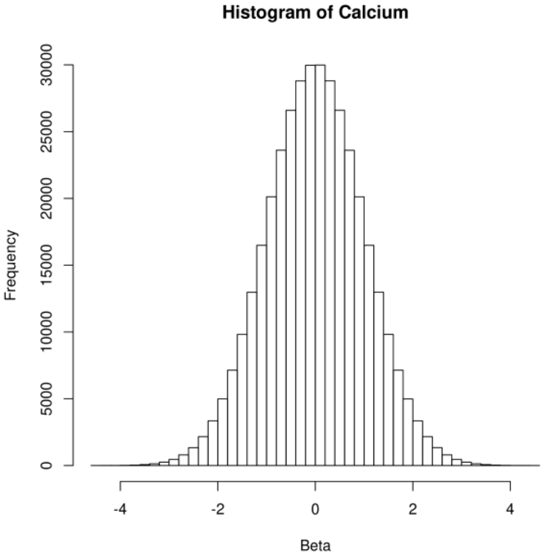

c) Albumin levels, structural variant analysis, European ancestry (N=378,395)

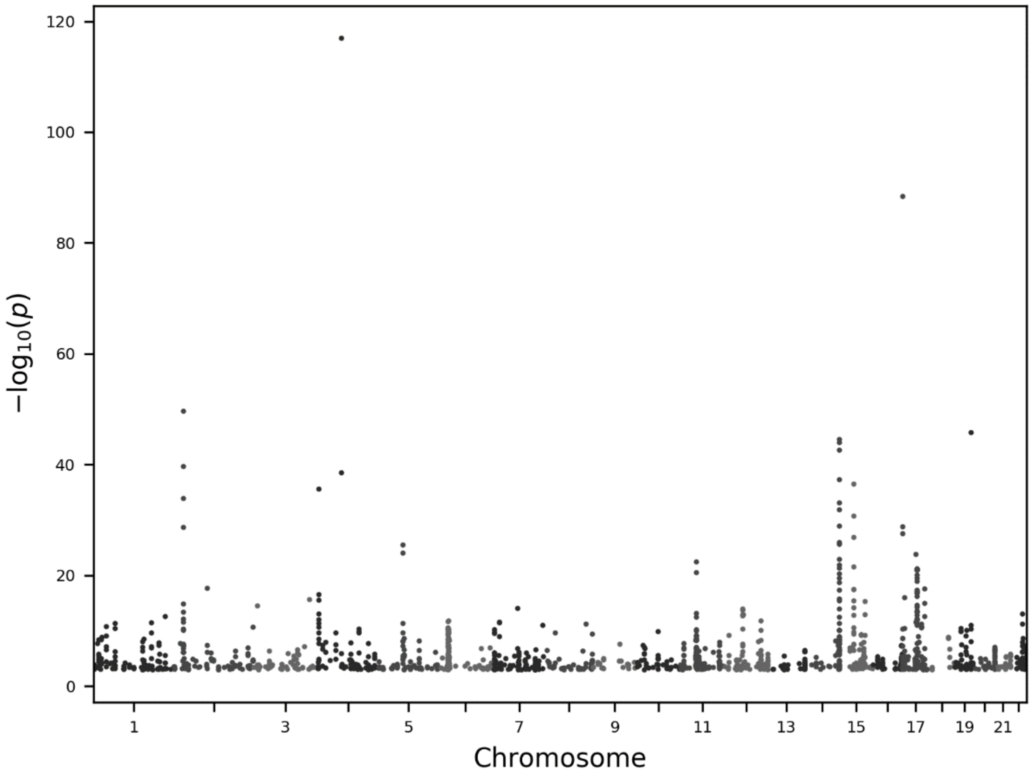

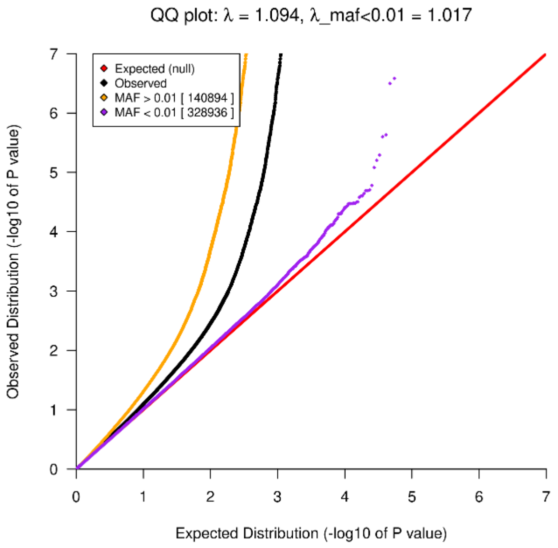

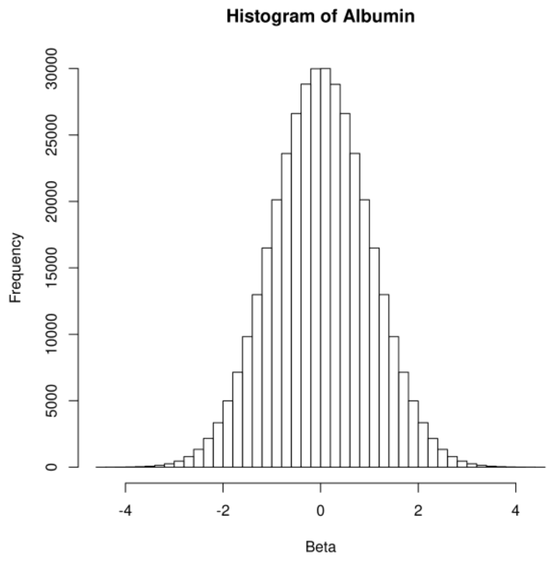

d) Standing height, SNV analysis, European ancestry (N=430,136)

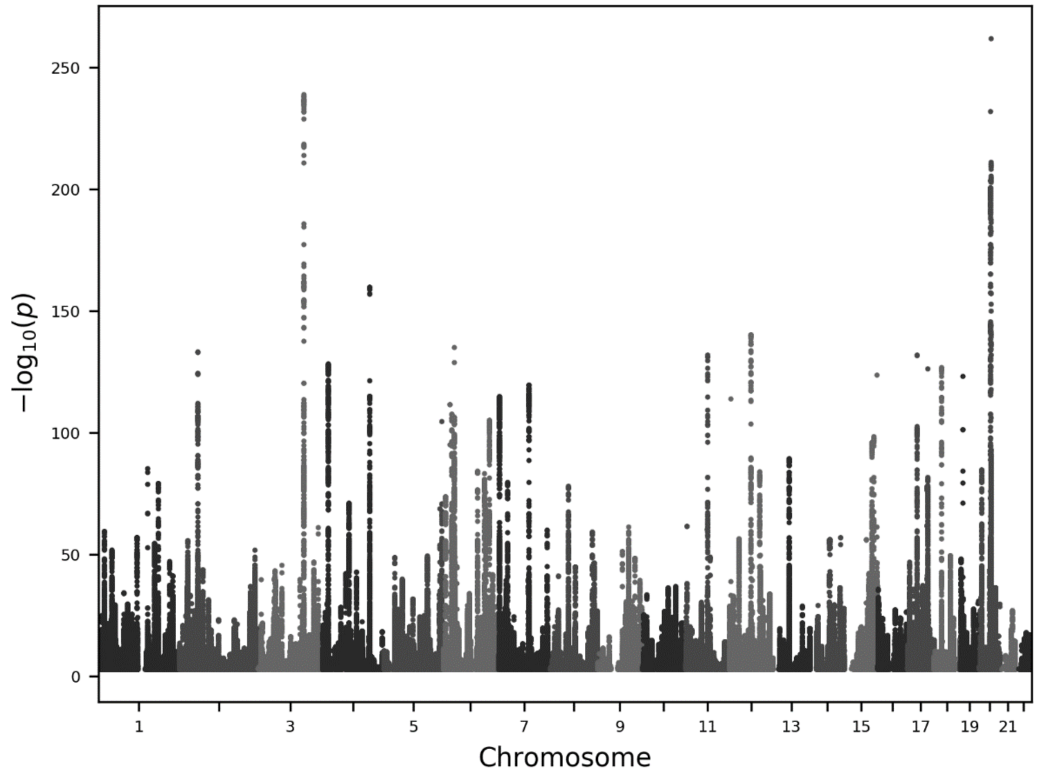

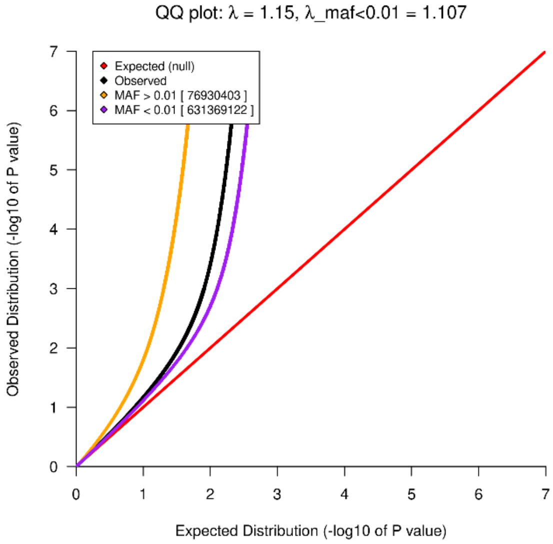

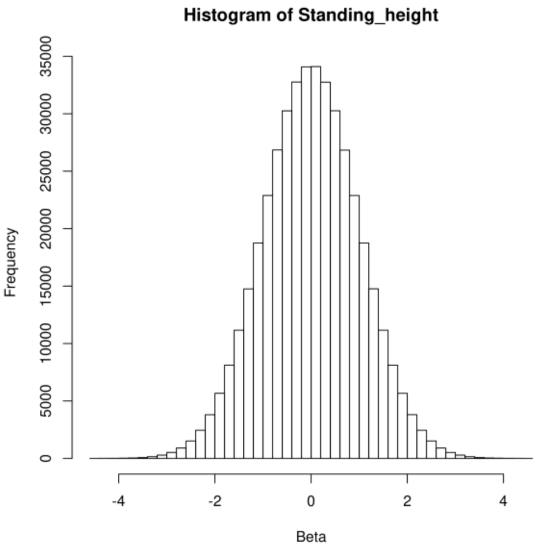

e) IGF-1 levels, SNV analysis, European ancestry (N=409,982)

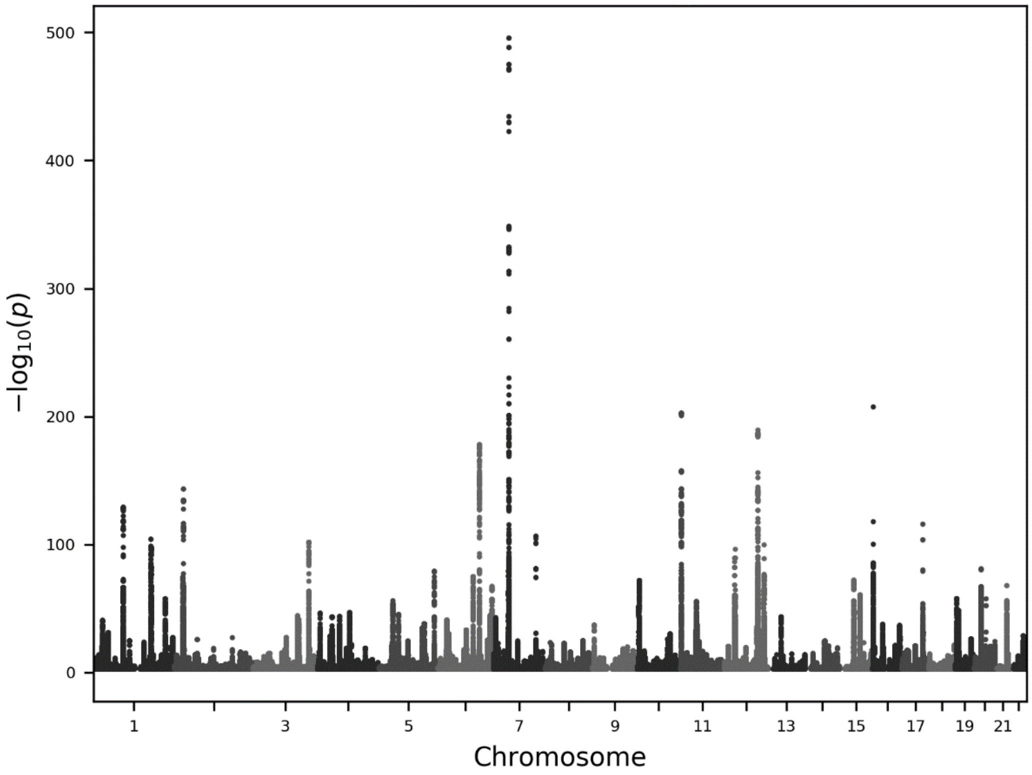

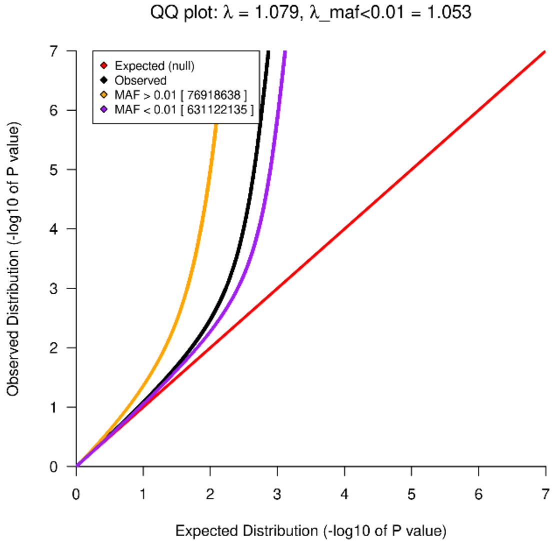

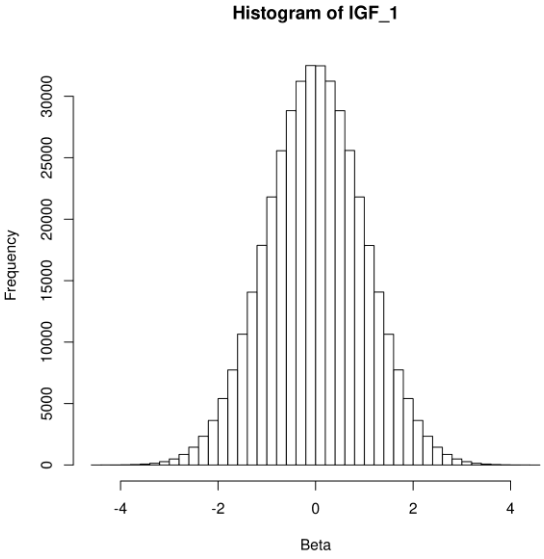

f) Mean corpuscular volume, SNV analysis, European ancestry, male sex (N=182,270)

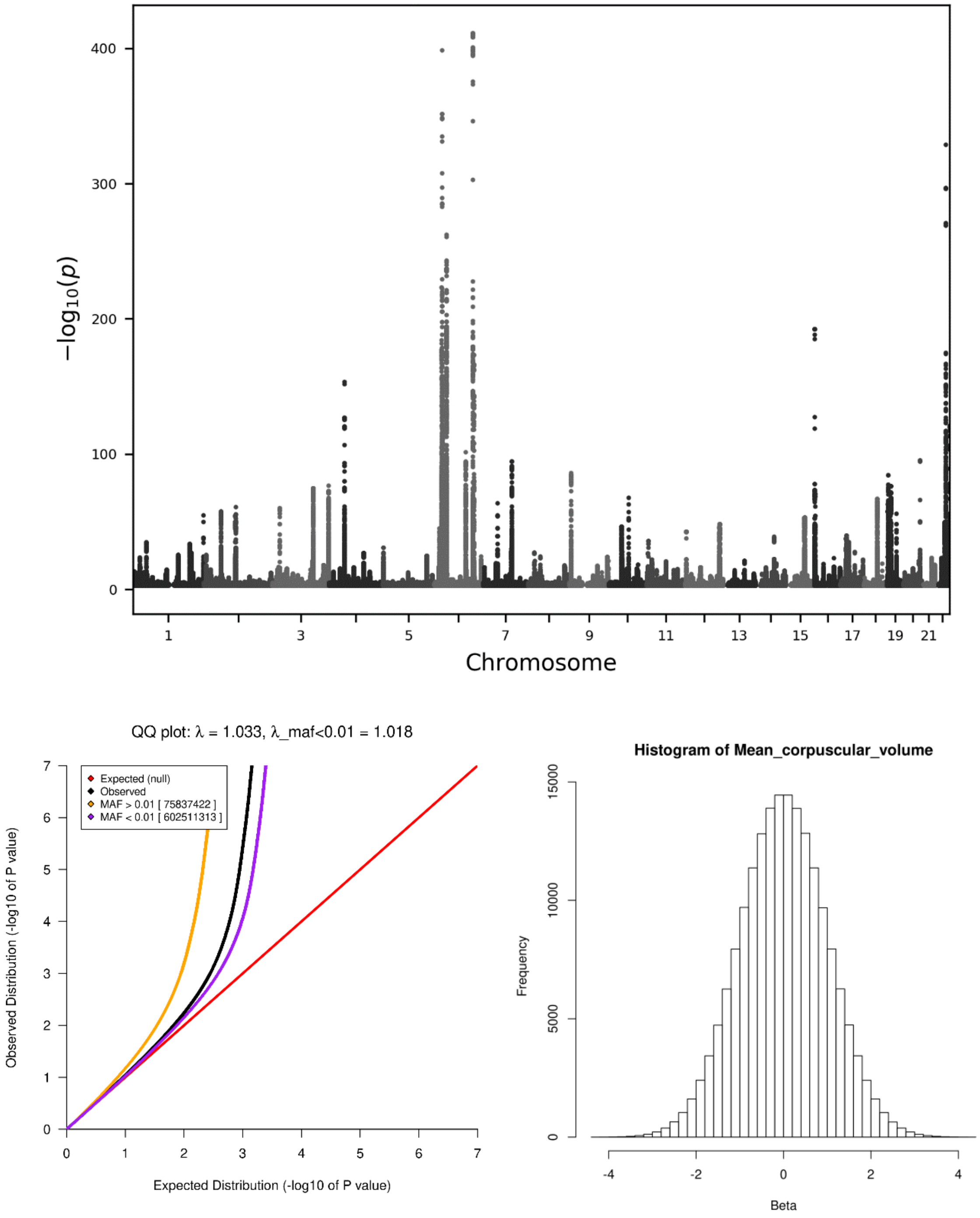

g) Age at menopause, structural variant analysis, European ancestry, female sex (N=141,129)

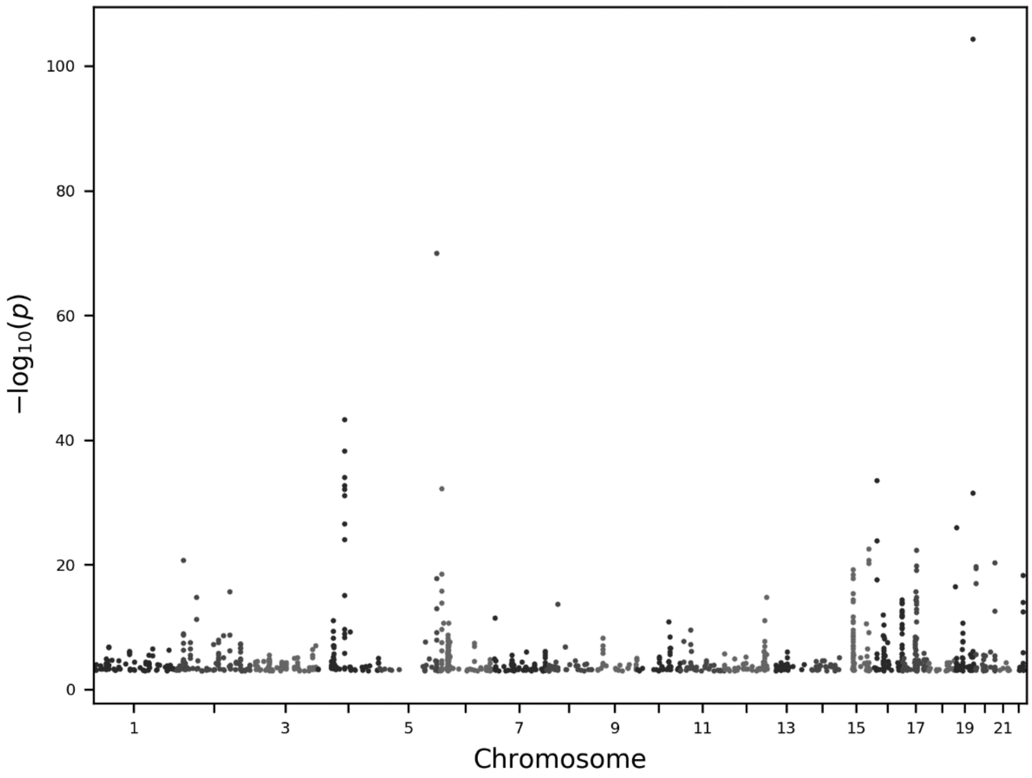

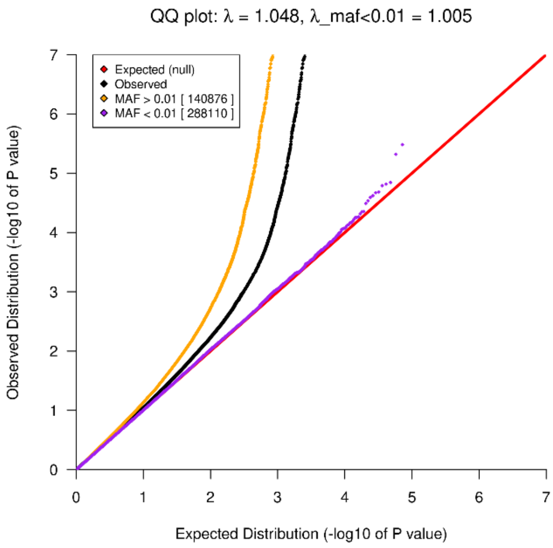

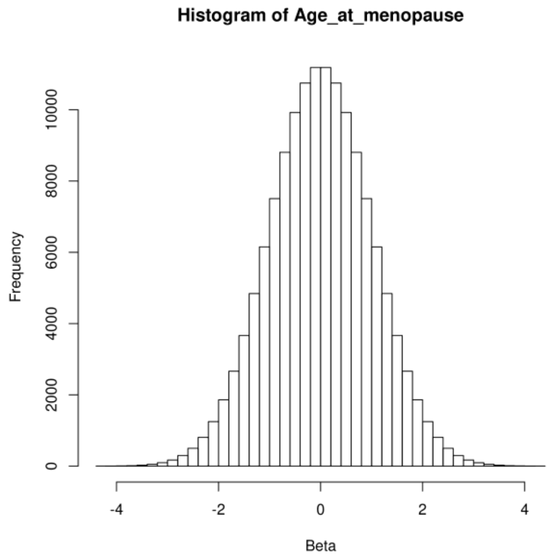

h) Non-high density lipoprotein, structural variant analysis, European ancestry (N=378,146)

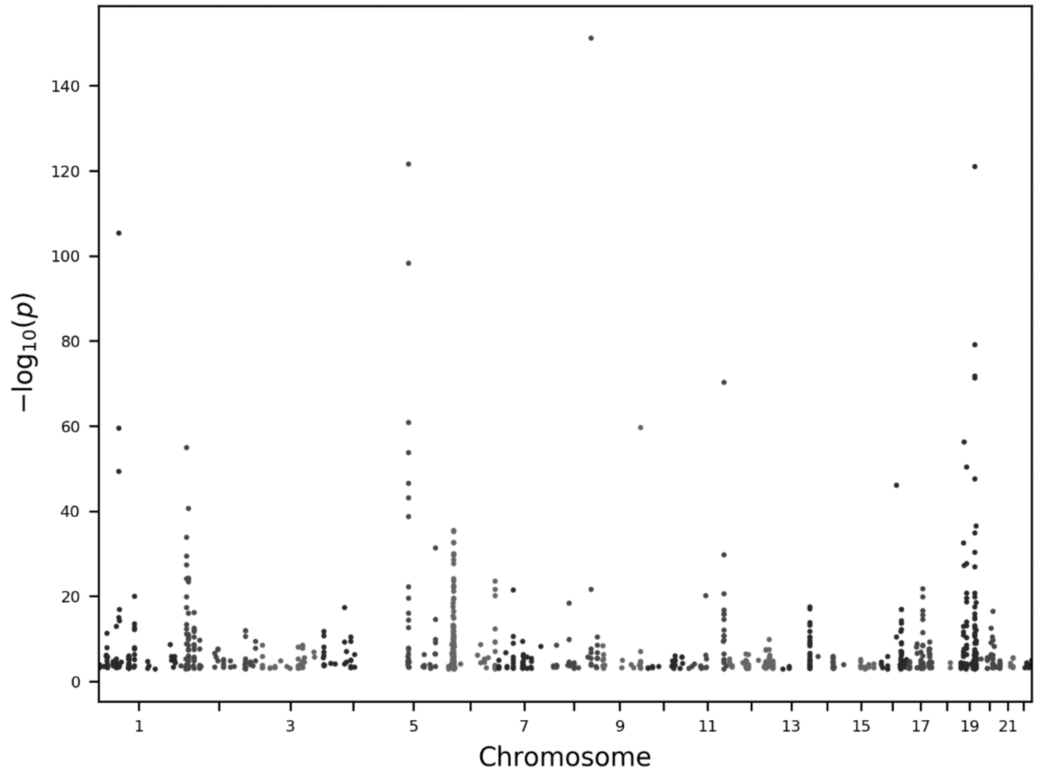

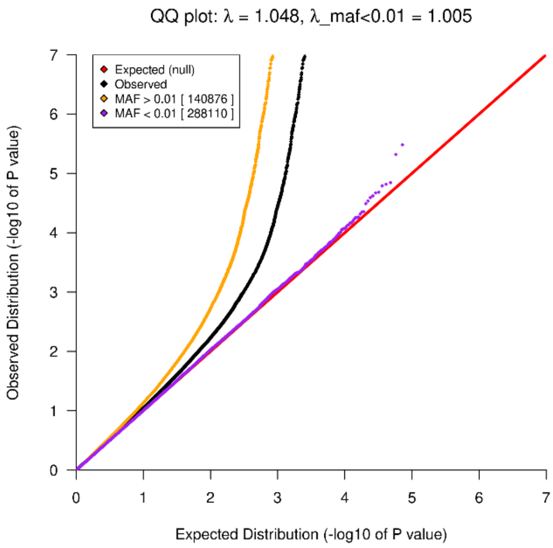

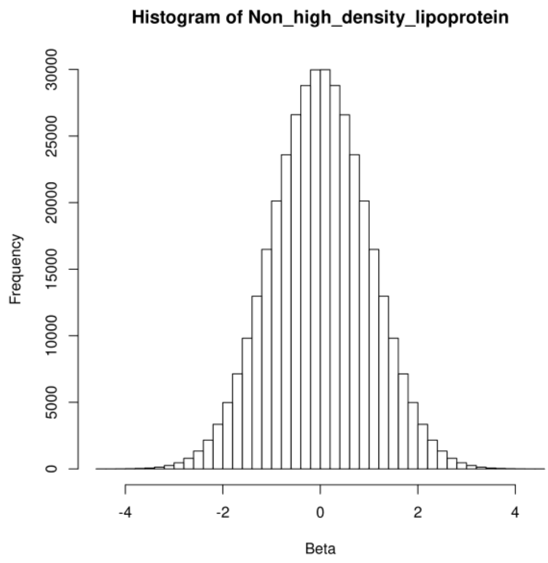

i) Non-high density lipoprotein, SNV analysis, African ancestry (N=8,359)

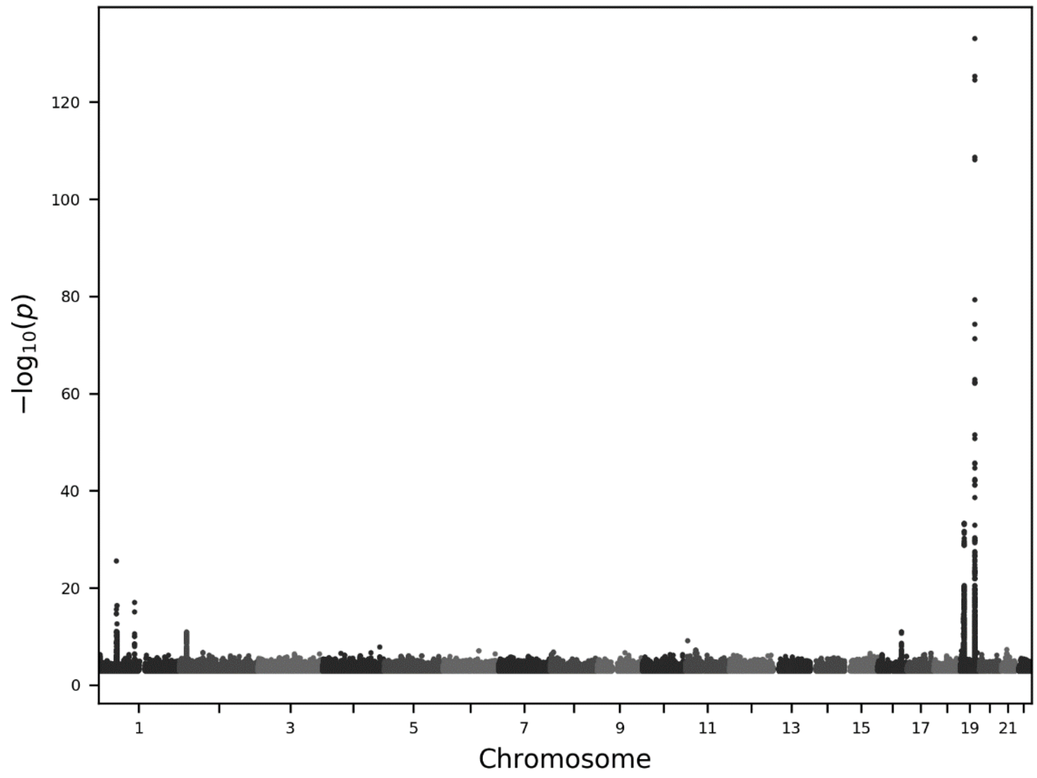

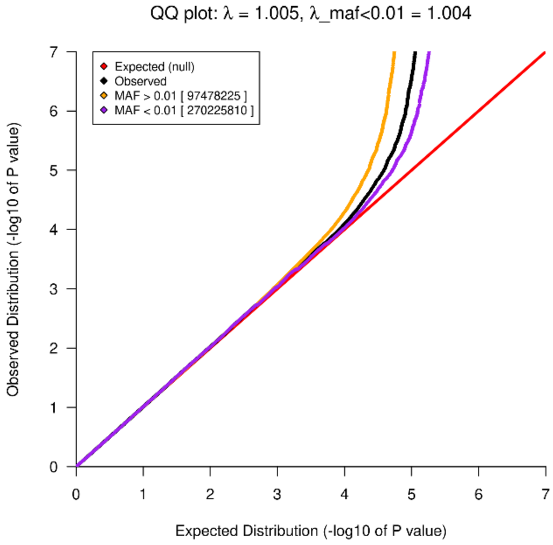

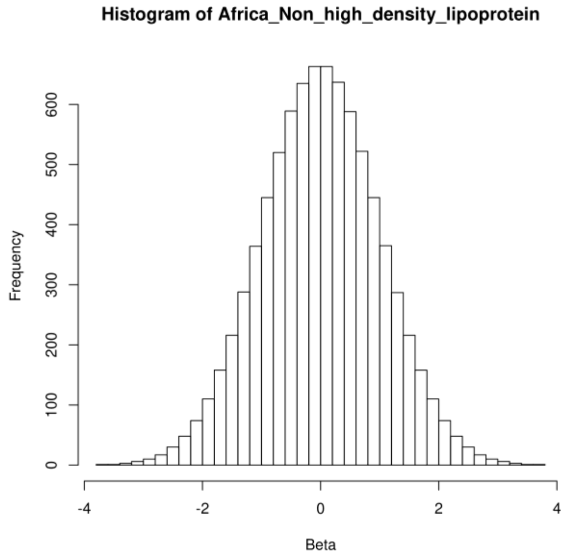

j) Hemoglobin concentration, SNV analysis, Asian ancestry (N=8,842)

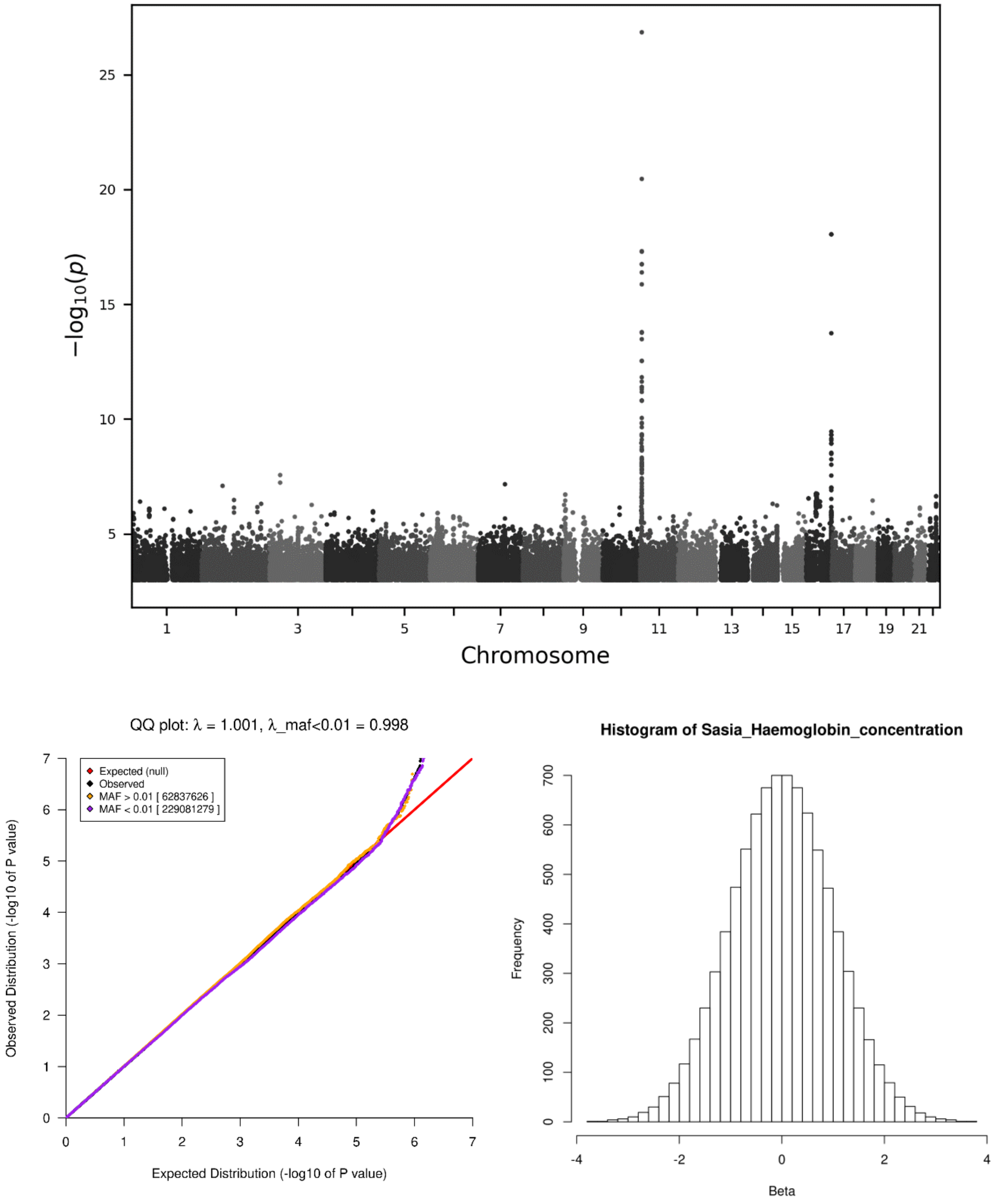

k) Age at menarche, SNV analysis, European ancestry (N=226,436)

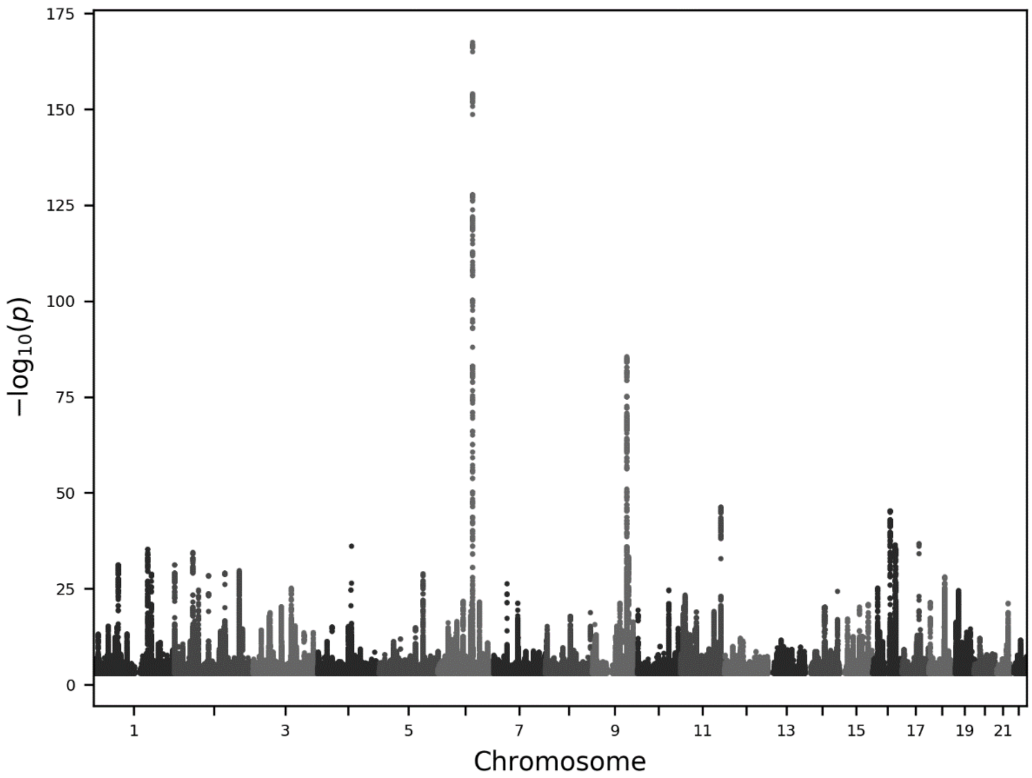

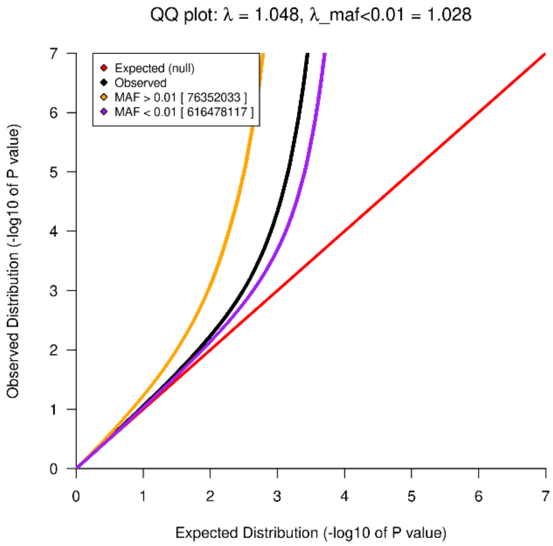

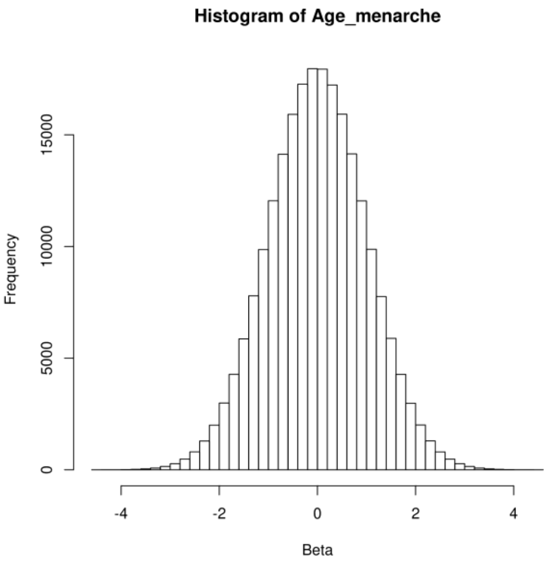

l) Urate levels, SNV analysis, European ancestry (N=411,640)

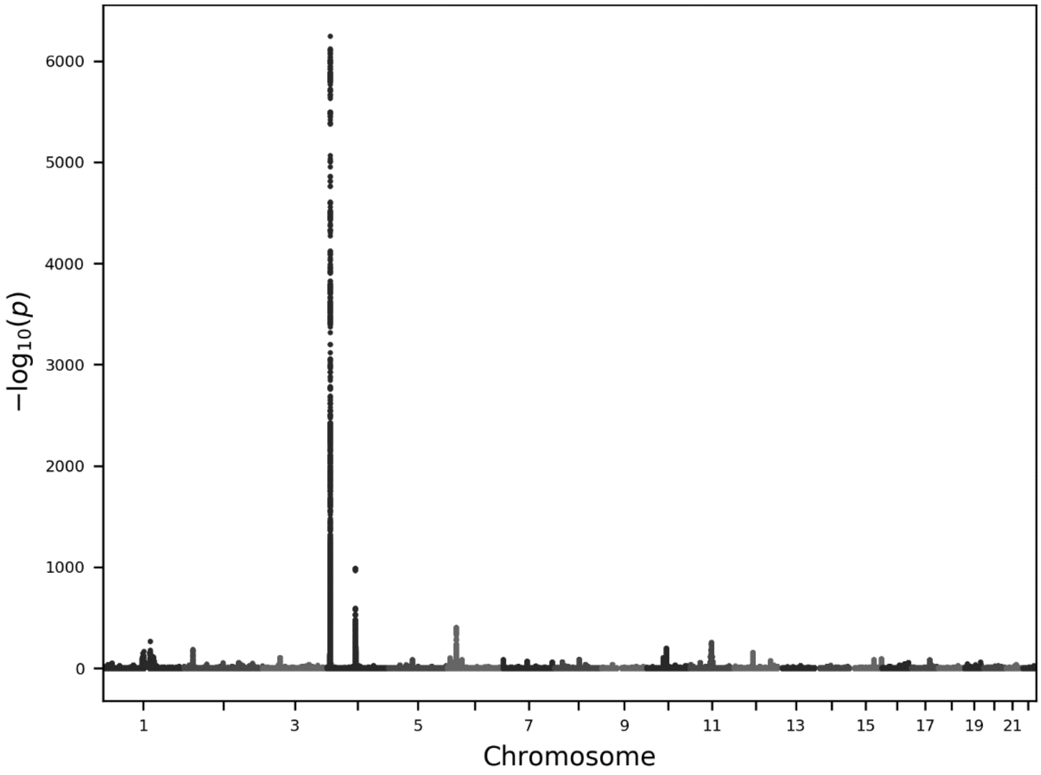

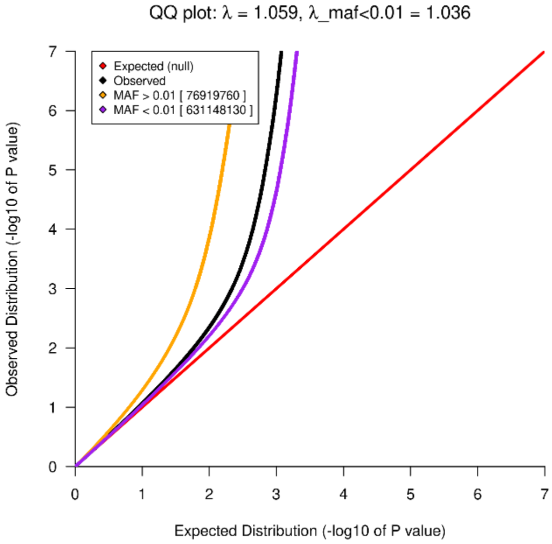

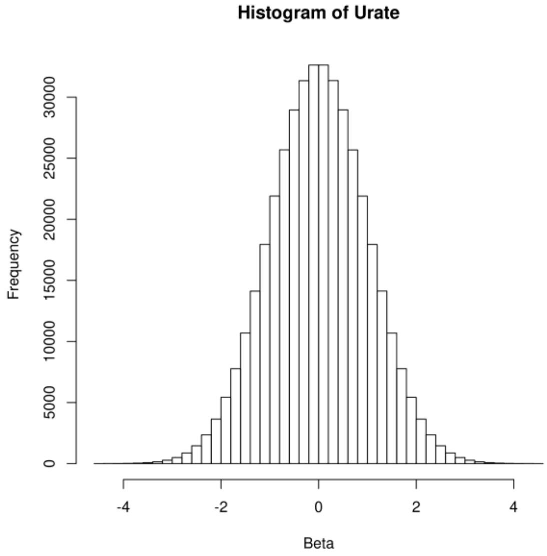

m) Glycine, metabolomics analysis, European ancestry (N=411,640)

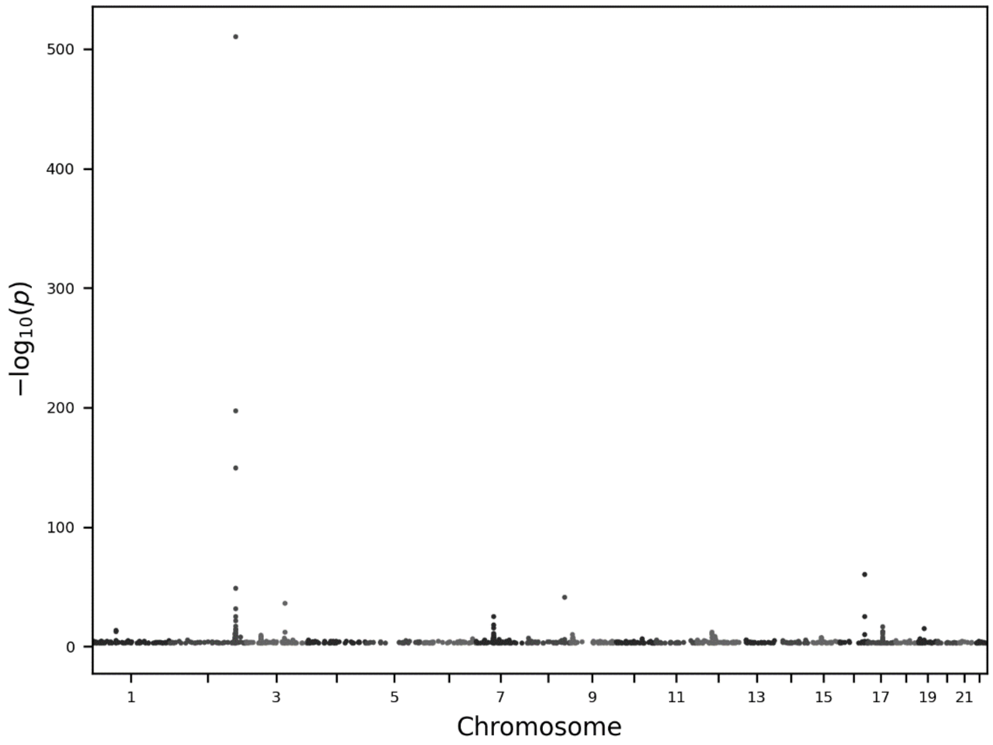

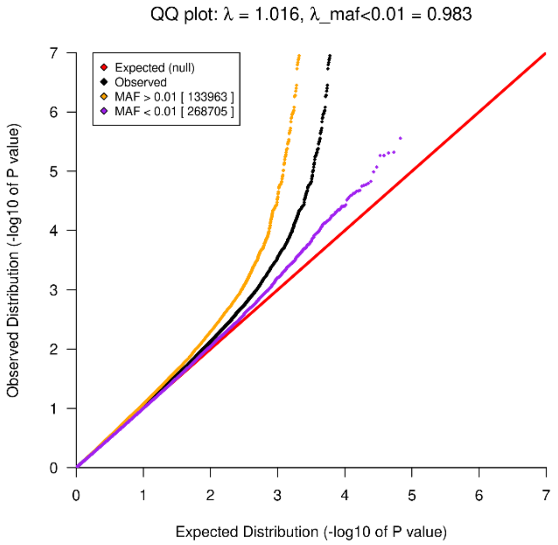

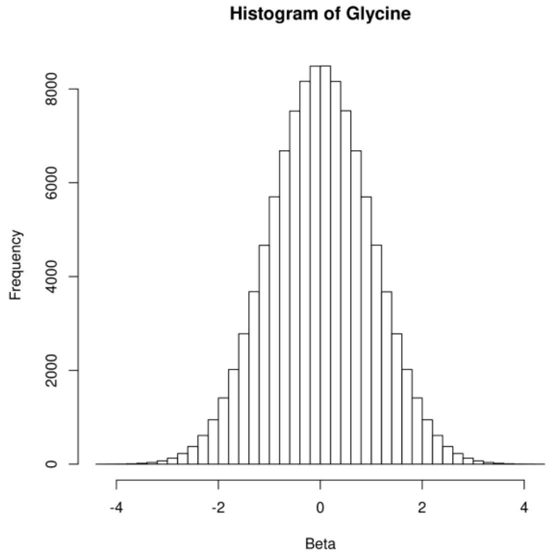

Supplementary Fig. 15 Manhattan plots and quantile-quantile (QQ) plots for case-control phenotypes with significant results reported in this manuscript.
For Manhattan plots, the x-axis represents chromosome locations and the y-axis shows the –log10 significance levels of the associations. For QQ plots, the inflation (λ) is shown in the title of each graph, for all variants and for rare variants only (λ_maf<0.01). P-values are computed using a two-sided χ^2-test.

a) *Hereditary ataxia, microsatellite analysis, European ancestry (Ncases=335, Ncontrols=430,603)*

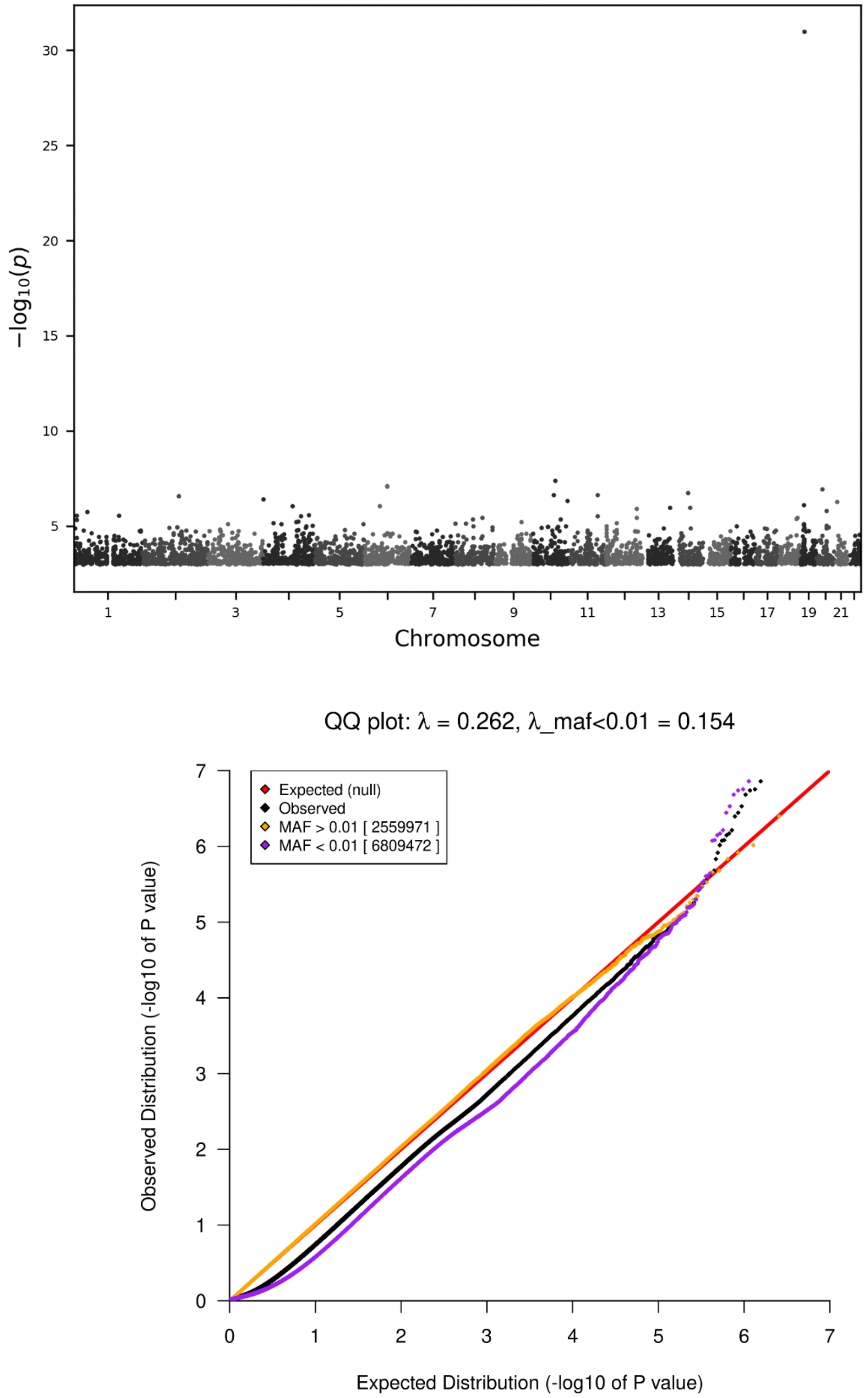

b) *Myotonic disorders, microsatellite analysis, European ancestry (Ncases=99, Ncontrols=430,839)*

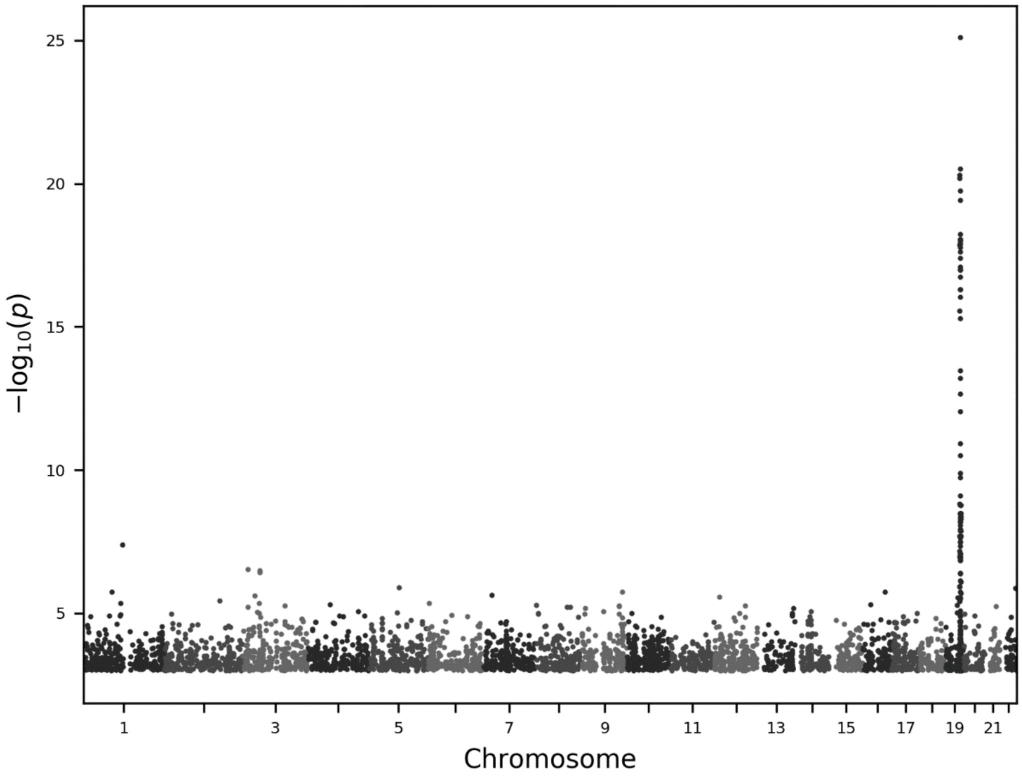

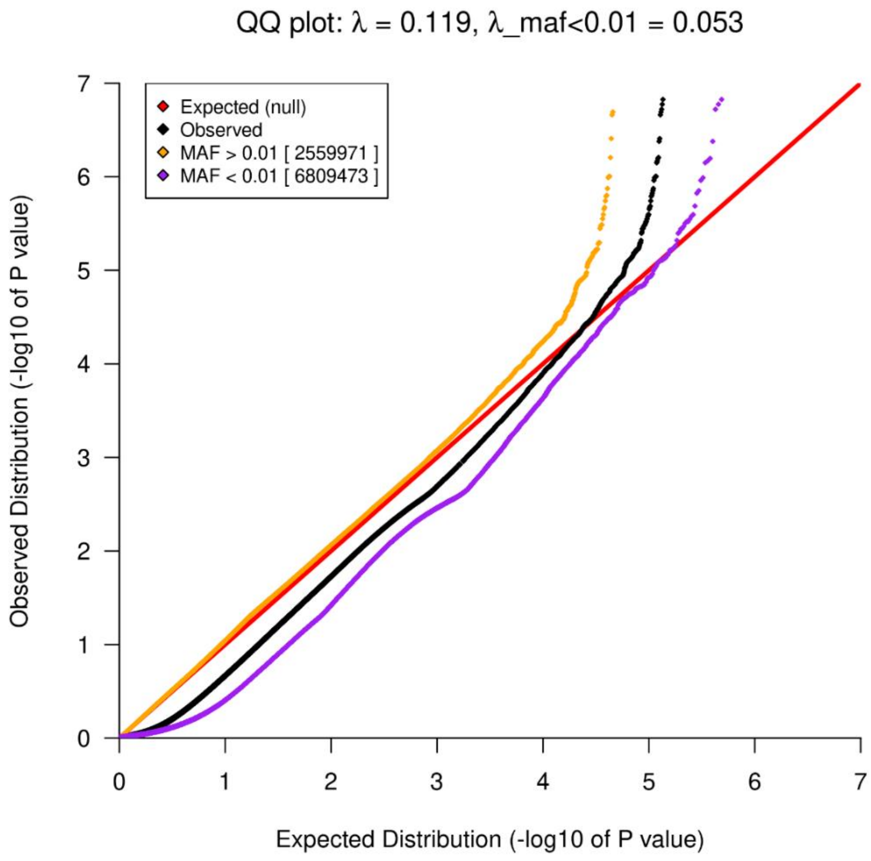

c) Gout, SNV analysis, European ancestry (Ncases=16,353, Ncontrols=414,694)

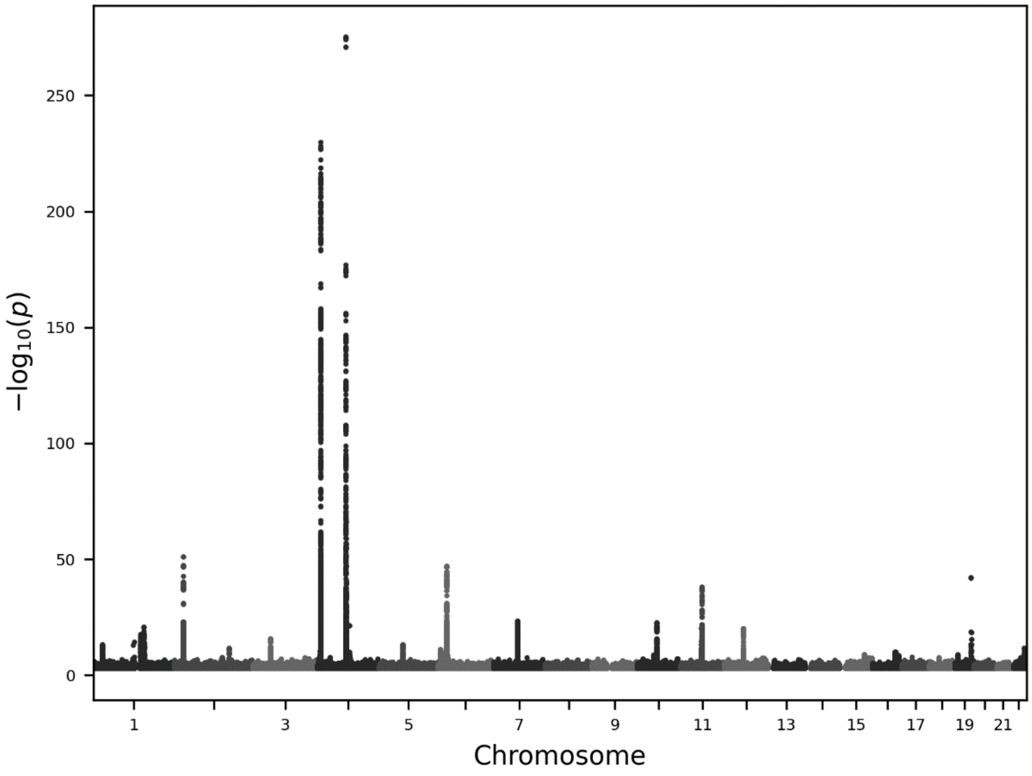

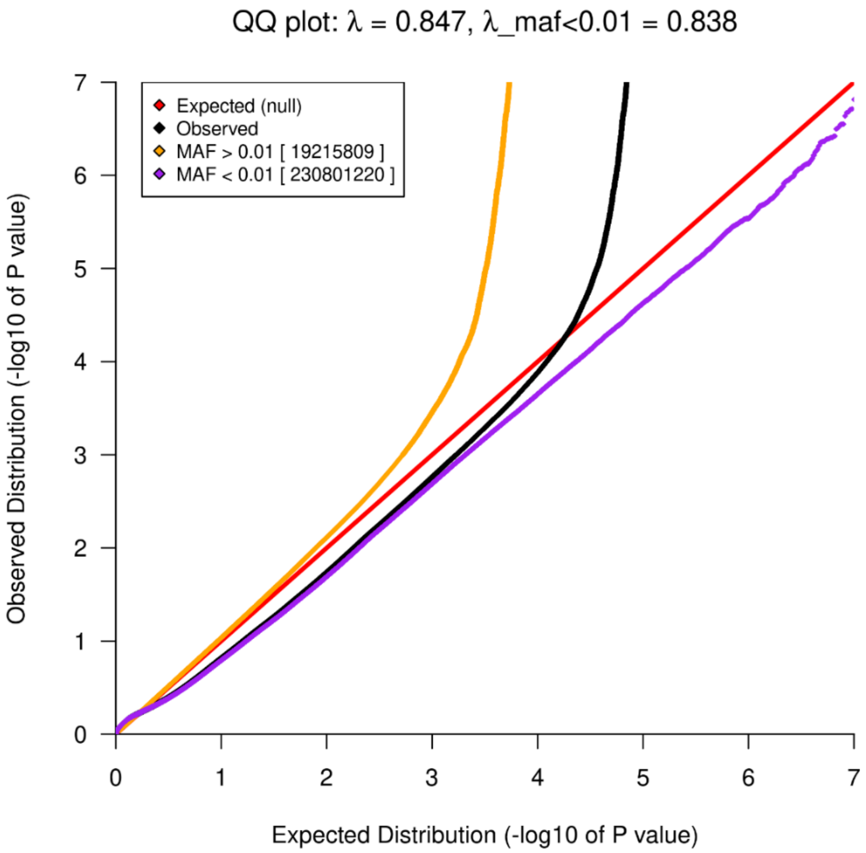

A)

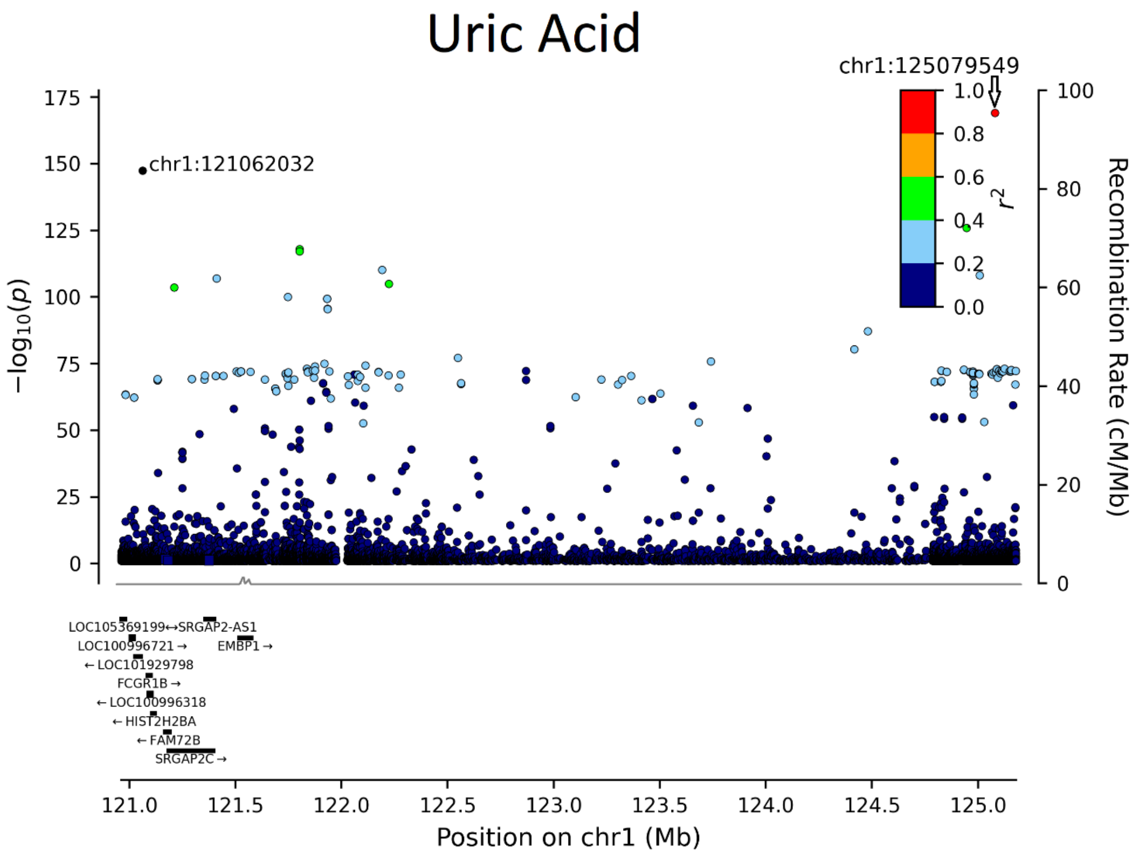

B)

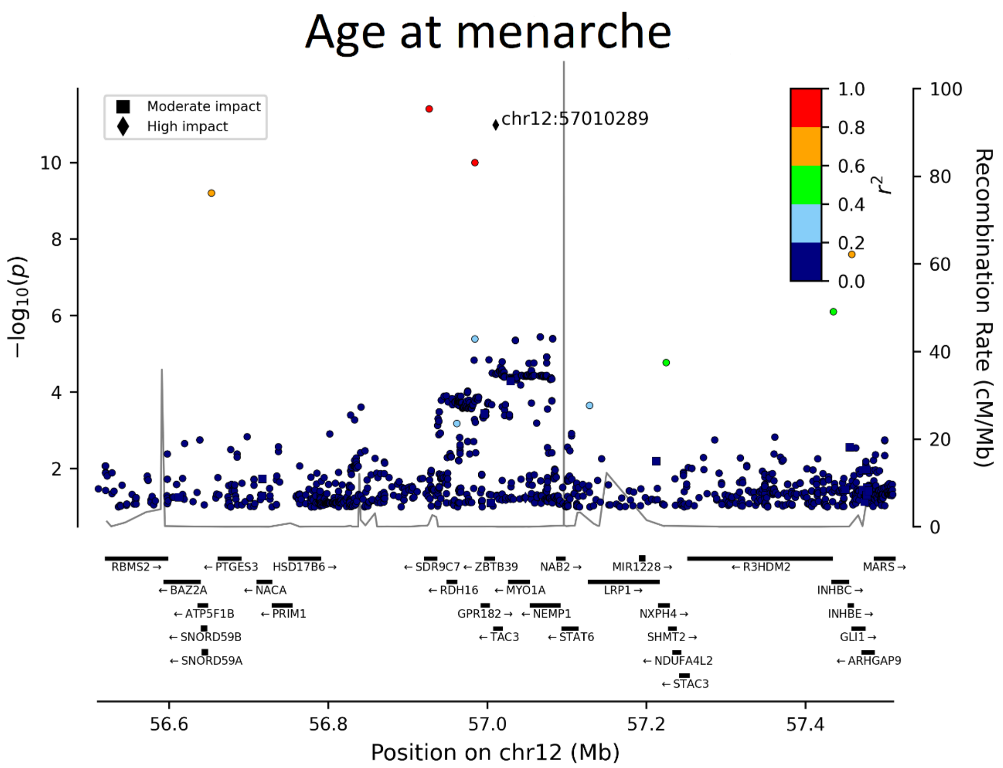

Supplementary Fig. 16 Locus plots.

A) Uric acid and B) Age at menarche associations. P-values in panel a) are computed using a two-sided χ^2-test.

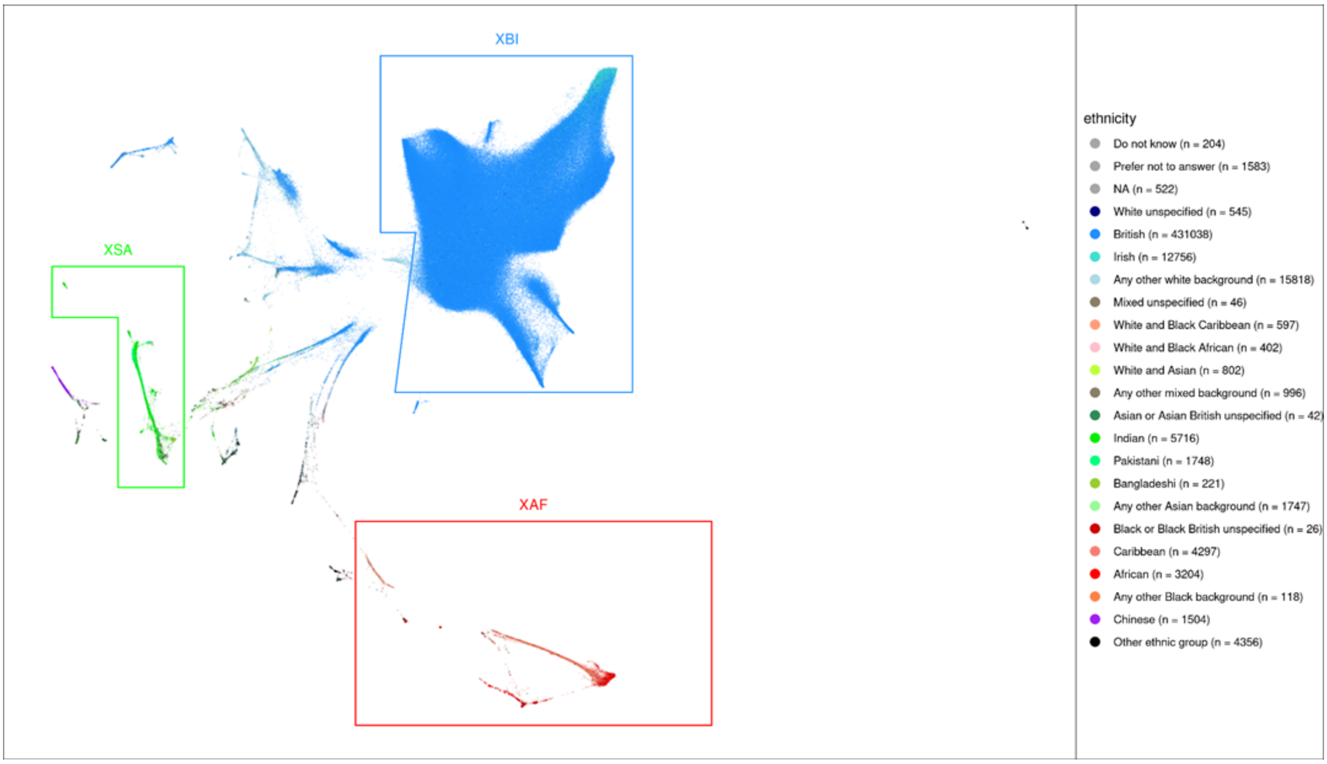

Supplementary Fig. 17 UMAP and ethnicity.
40 genetic principal components provided by UKB reduced to a latent space of 2 dimensions using UMAP (x and y axes). Individuals are colored according to self-identified ethnicity. The regions defined to delineate the three cohorts XAF, XBI, and XSA are indicated.

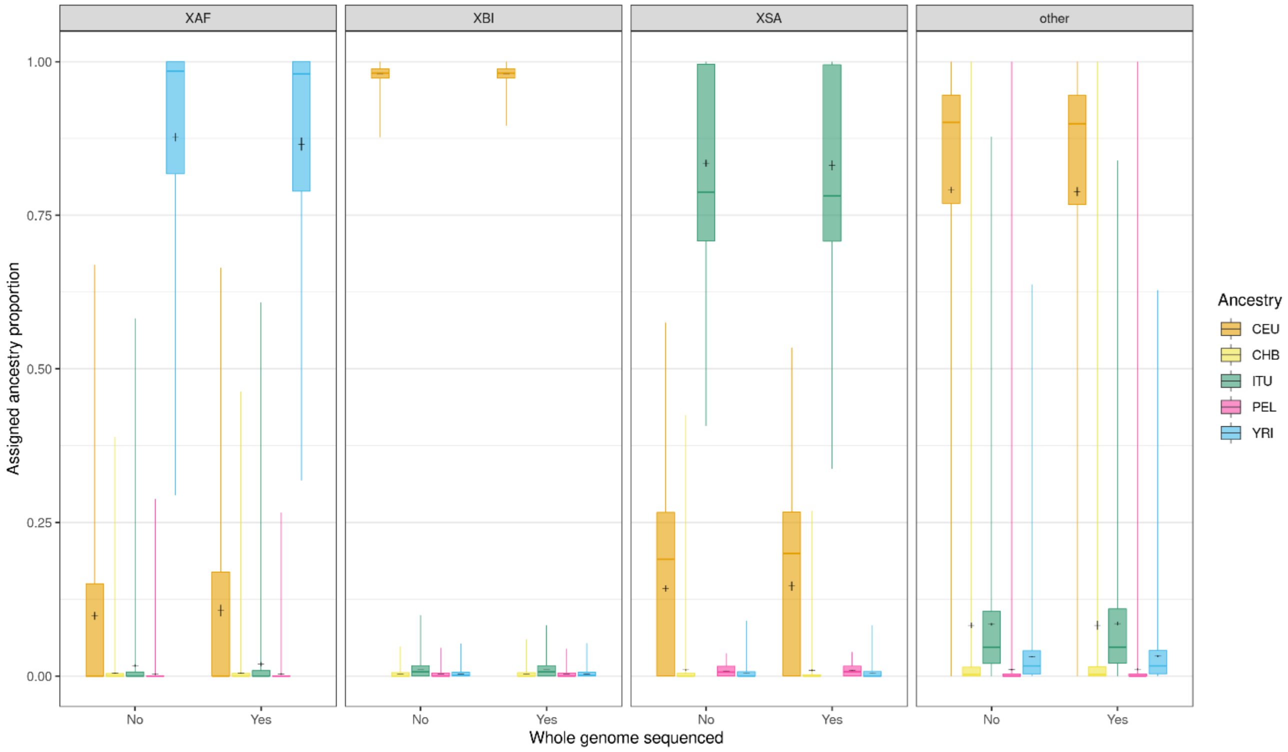

Supplementary Fig. 18 Cohort ADMIXTURE summaries.
Boxplots of ADMIXTURE-assigned ancestry per cohort. Horizontal lines within boxes represent medians, and tops and bottoms of boxes indicate 75th and 25th percentile values respectively. Whiskers (colored vertical lines) extend to minimum and maximum values. Overlaid black horizontal lines represent means, and black vertical lines extending above and below the horizontal lines represent +3 and -3 standard errors respectively. CEU (Northern Europeans from Utah), CHB (Han Chinese in Beijing), ITU (Indian Telugu in the UK), PEL (Peruvians in Lima), and YRI (Yoruba in Ibadan, Nigeria).

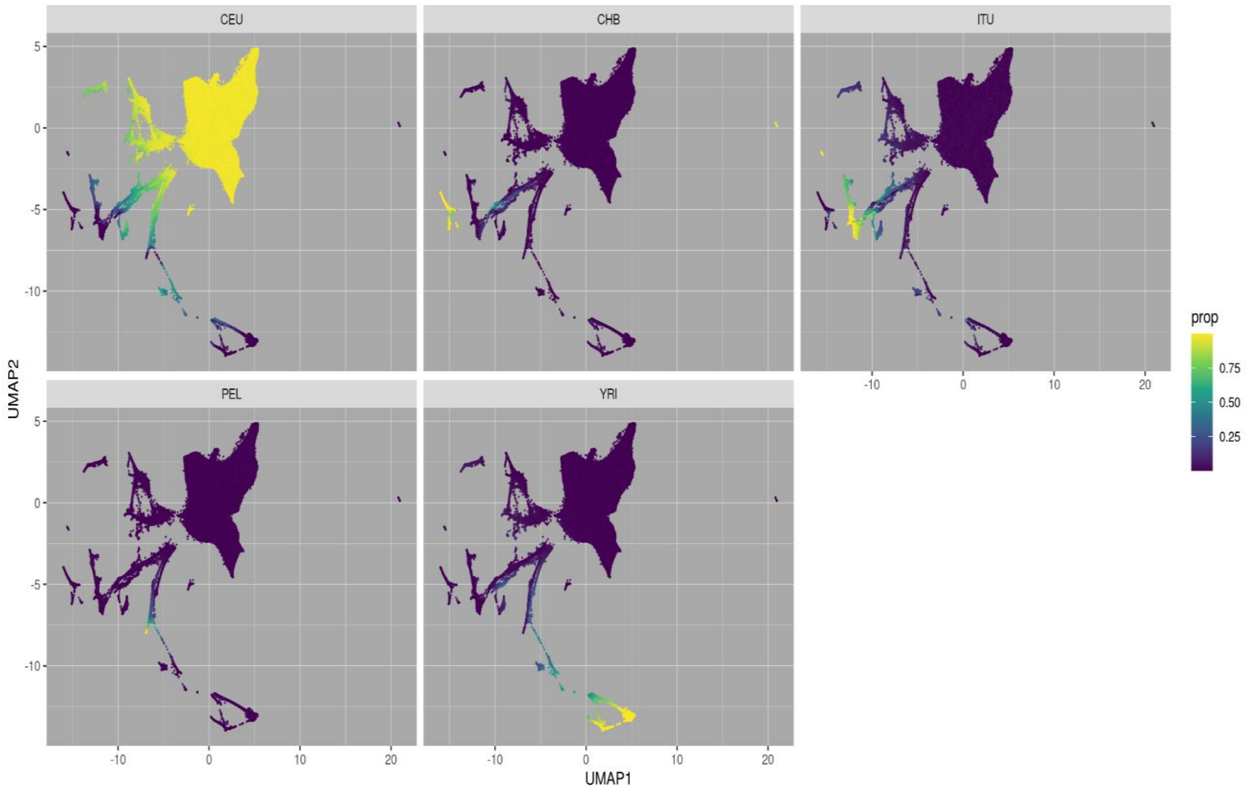

Supplementary Fig. 19 UMAP ADMIXTURE.
40 genetic principal components provided by UKB reduced to a latent space of 2 dimensions using UMAP (x and y axes). Individuals are colored according to proportion of ancestry assigned by supervised ADMIXTURE from five 1000GP training populations (facet headings): CEU (Northern Europeans from Utah), CHB (Han Chinese in Beijing), ITU (Indian Telugu in the UK), PEL (Peruvians in Lima), and YRI (Yoruba in Ibadan, Nigeria).

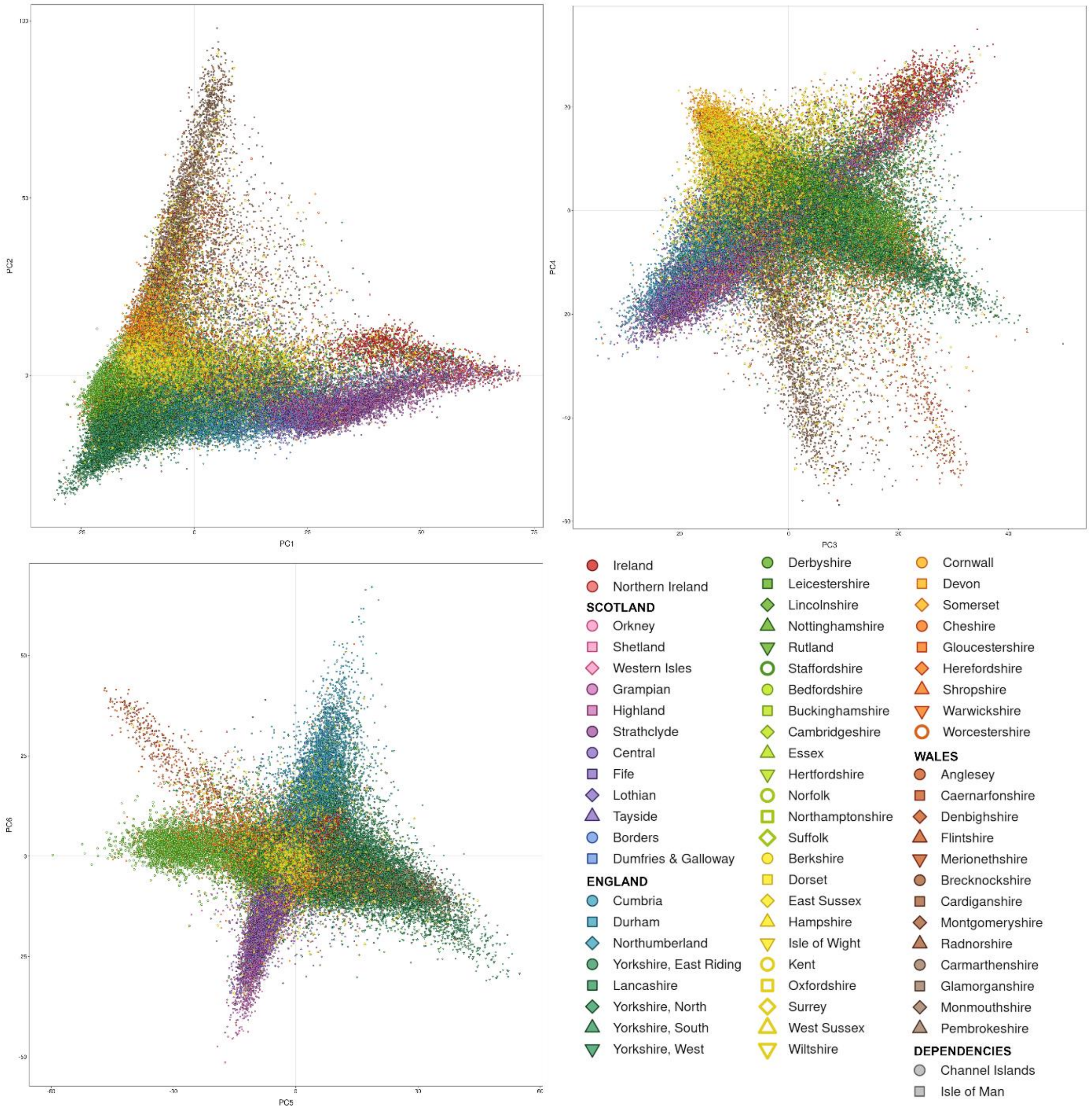

Supplementary Fig. 20 The first six principal components of the XBI cohort.
Plots show PC1 vs PC2, PC3 vs PC4 and PC5 vs PC6. Points represent individuals, colored by place of birth. To show geographic structure in the UK more clearly, we do not show individuals who report being born in urban areas with many internal migrants (Tyne & Wear, Merseyside, Greater Manchester, West Midlands, Bristol, London) or places outside the British-Irish Isles.

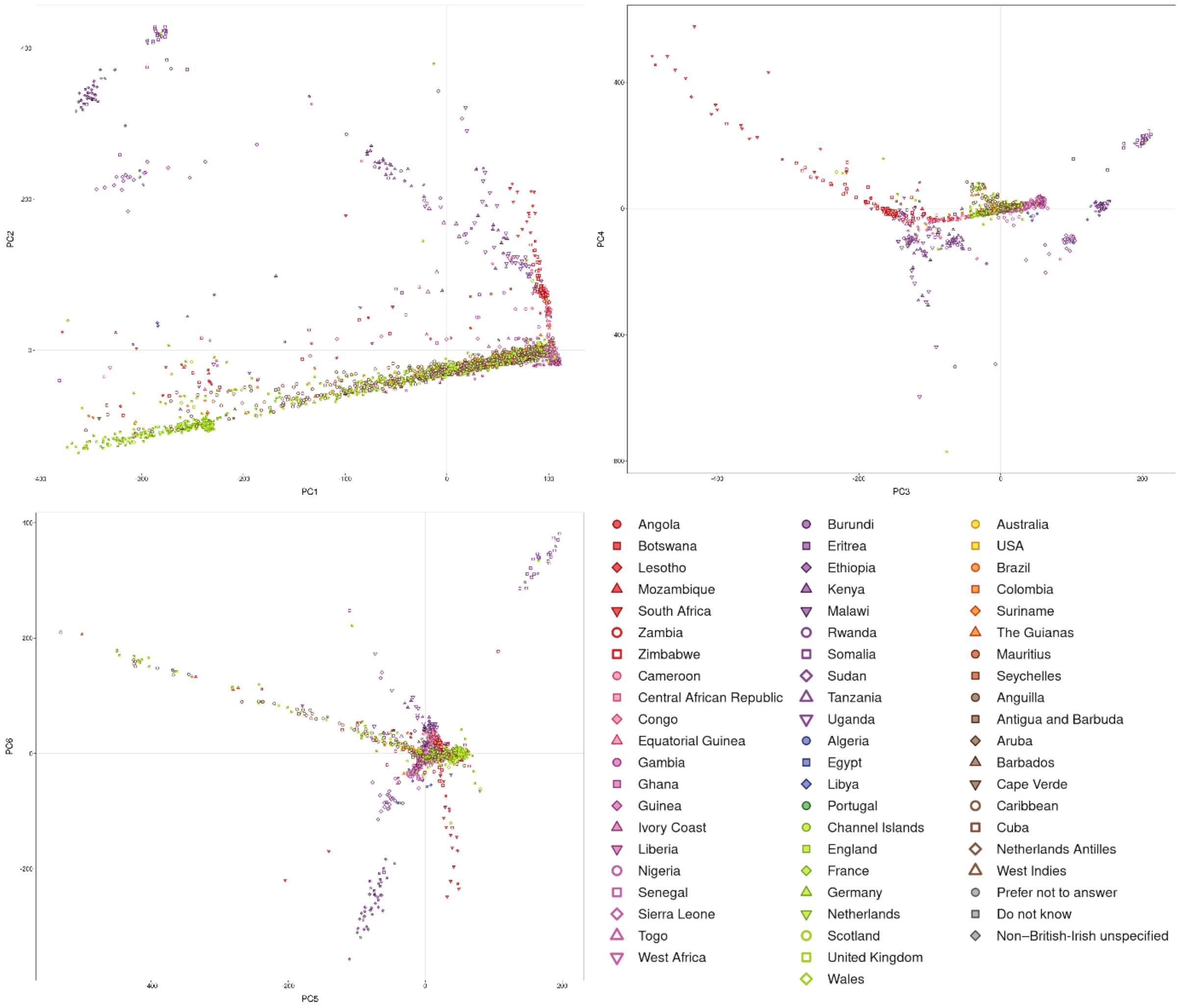

Supplementary Fig. 21 The first six principal components of the XAF cohort.
Plots show PC1 vs PC2, PC3 vs PC4 and PC5 vs PC6. Points represent individuals, colored by place of birth.

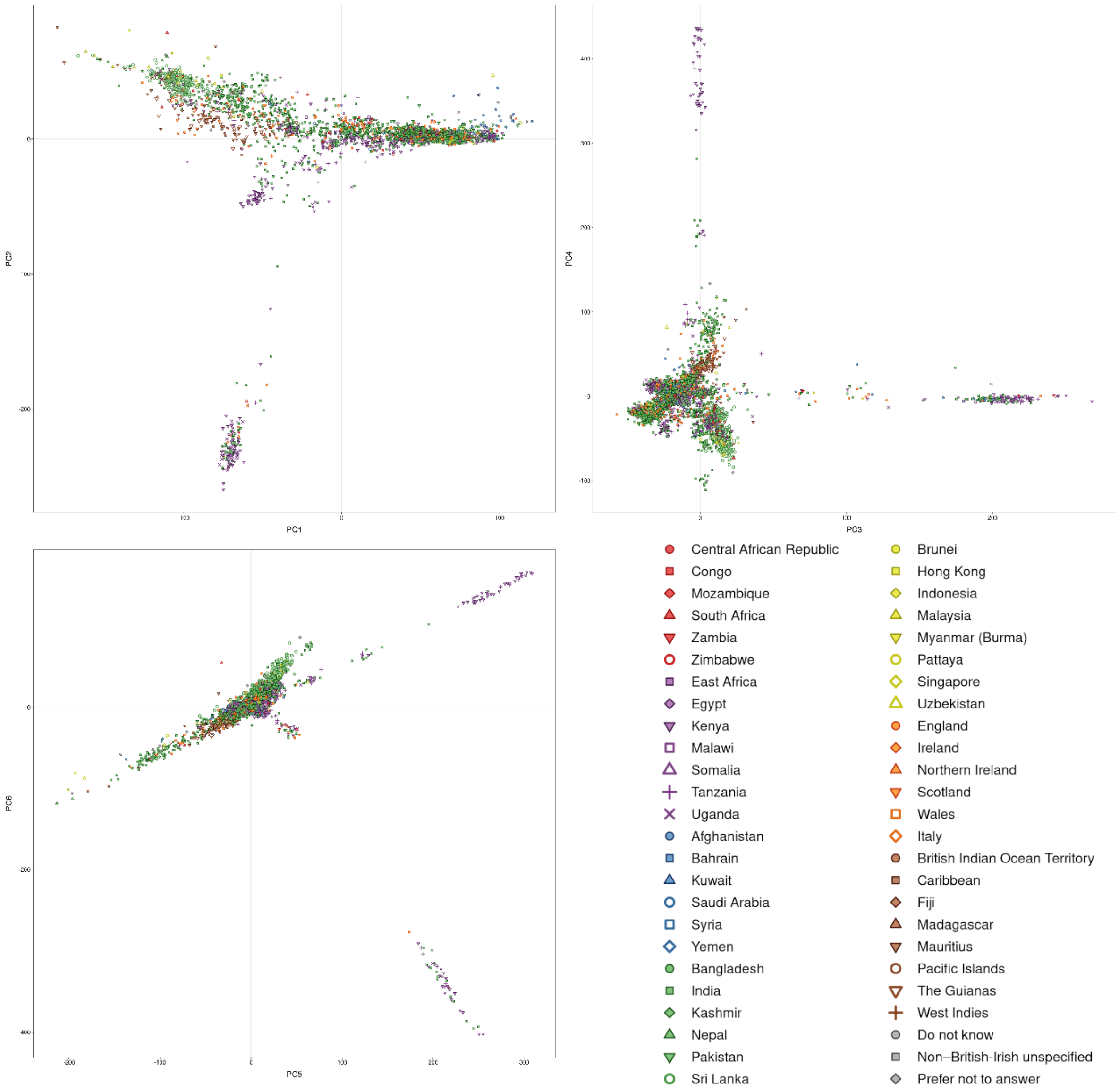

Supplementary Fig. 22 The first six principal components of the XSA cohort.
Plots show PC1 vs PC2, PC3 vs PC4 and PC5 vs PC6. Points represent individuals, colored by place of birth.

Supplementary Tables

A) SNP+Indel

GIAB sample	#Variants	GATK Sensitivity	GATK Precision	GATK F1-score	GraphTyper Sensitivity	GraphTyper Precision	GraphTyper F1-score
HG001	30,717	98.09%	98.90%	98.49%	98.97%	99.29%	99.13%
HG002	29,802	98.14%	99.03%	98.59%	98.84%	99.36%	99.10%
HG003	28,379	98.16%	99.10%	98.63%	99.02%	99.21%	99.11%
HG004	28,539	98.11%	99.02%	98.56%	99.03%	99.48%	99.26%
HG005	26,846	98.47%	99.02%	98.74%	99.08%	99.48%	99.28%
HG006	27,546	98.77%	99.11%	98.94%	99.22%	99.28%	99.25%
HG007	28,798	98.63%	99.21%	98.92%	99.14%	99.29%	99.21%
Average	28,661	98.34%	99.06%	98.70%	99.04%	99.34%	99.19%

B) SNP

GIAB sample	#Variants	GATK Sensitivity	GATK Precision	GATK F1-score	GraphTyper Sensitivity	GraphTyper Precision	GraphTyper F1-score
HG001	26,377	99.50%	99.07%	99.28%	99.63%	99.29%	99.46%
HG002	25,747	99.45%	99.09%	99.27%	99.46%	99.36%	99.41%
HG003	24,450	99.43%	99.19%	99.31%	99.56%	99.20%	99.38%
HG004	24,428	99.47%	99.16%	99.31%	99.60%	99.48%	99.54%
HG005	23,465	99.60%	99.14%	99.37%	99.44%	99.49%	99.46%
HG006	24,226	99.63%	99.18%	99.40%	99.61%	99.27%	99.44%
HG007	25,257	99.59%	99.30%	99.44%	99.53%	99.29%	99.41%
Average	24,850	99.52%	99.16%	99.34%	99.55%	99.34%	99.44%

C) Indel

GIAB sample	#Variants	GATK Sensitivity	GATK Precision	GATK F1-score	GraphTyper Sensitivity	GraphTyper Precision	GraphTyper F1-score
HG001	4,340	89.46%	97.30%	93.21%	94.94%	99.59%	97.21%
HG002	4,055	89.81%	98.42%	93.92%	94.85%	99.42%	97.08%
HG003	3,929	90.26%	98.12%	94.03%	95.61%	99.54%	97.54%
HG004	4,111	89.93%	97.68%	93.64%	95.59%	99.43%	97.47%
HG005	3,381	90.47%	97.80%	93.99%	96.50%	99.34%	97.90%
HG006	3,320	92.45%	98.35%	95.31%	96.34%	99.65%	97.97%
HG007	3,541	91.68%	98.25%	94.85%	96.28%	99.51%	97.87%
Average	3,811	90.58%	97.99%	94.14%	95.73%	99.50%	97.58%

Supplementary Table 1 Genome in a bottle (GIAB) v3.3.2 truth set comparison of GATK and GraphTyper in 500 random regions.
F1-score is the harmonic mean of Sensitivity and Precision. A) all variant types, B) SNPs only C) Indels only.

A)

Method	FDR	TP	#Variants
GATK	9.97%	17,140,110	19,038,309
GraphTyper	6.31%	17,915,210	19,123,669
GraphTyperHQ	1.45%	16,768,945	17,016,415

B)

Method	ICPM	Non-ref consistency	Number of non-ref calls
GATK	78.1	95.21%	68,537,823
GraphTyper	70.3	95.81%	70,442,413
GraphTyperHQ	11.8	99.22%	63,556,940

Supplementary Table 2 Genotype consistency
A) Estimate of false discovery rate (FDR) and number of true positive (TP) variants among the 28 parent-offspring trios. The estimates are determined from the allele transmission ratios from parent to offspring. B) Genotype consistency across among the 14 monozygotic twin pairs. ICPM = number of inconsistent genotypes per 1Mb.

A)

Method	Total checks	Error rate
GATK	1,277,130	1.19%
GraphTyper	1,339,337	1.12%

B)

	GATK	GraphTyper
Total variants	166,315	162,773
SNPs only	137,277	125,282
Indels only	29,038	37,491
True positive estimate	145,882	151,838
SNPs only	119,682	117,659
Indels only	26,200	34,179
False discovery rate estimate	12.28%	6.72%
SNPs only	12.82%	6.08%
Indels only	9.77%	8.83%

C)

Method	Non-Ref Variants	Consistent	Error rate
GATK	597,882	564,031	5.66%
GraphTyper	603,589	578,763	4.11%

Supplementary Table 3 Analysis of variant transmission of related samples in the 500 randomly selected 50kb test regions.

A) Number of inheritance errors among the 28 parent-offspring trios. B) Estimates of number True Positives and False discovery rate in GATK and GraphTyper datasets in the trios. The estimates are determined from the allele transmission ratios from parent to offspring. C) Genotype consistency among the 14 pairs of monozygote twins.

Minimum number of carriers	Frequency threshold	GATK N imputed	GATK N markers	GATK Imputed ratio	GraphTyper N imputed	GraphTyper N markers	GraphTyper Imputed ratio
SNPs		54001	200471	26.9%	58494	197508	29.6%
2640	1.0%	3157	3439	91.7%	3380	3500	96.5%
264	0.1%	2480	3225	76.8%	2623	2770	94.6%
26	0.01%	7436	10467	71.0%	7859	9367	83.9%
13	0.005%	5491	7557	72.6%	5857	7331	79.8%
6	0.002%	11326	17013	66.5%	12230	16884	72.4%
3	0.001%	16503	33921	48.6%	18095	33851	53.4%
1	0.0002%	7608	124849	6.0%	8450	123805	6.8%
Indels		6124	21720	30.4%	7876	20218	39.0%
2640	1.0%	842	935	90.0%	1132	1254	90.2%
264	0.1%	602	854	70.4%	790	917	86.1%
26	0.01%	1037	1861	55.7%	1327	1723	77.0%
13	0.005%	570	1054	54.0%	673	966	69.6%
6	0.002%	1038	1954	53.1%	1172	1800	65.1%
3	0.001%	1352	3377	40.0%	1521	3096	49.1%
1	0.0002%	683	11685	5.8%	743	10462	7.1%

Supplementary Table 4 Comparison of imputation of variants from the GATK and GraphTyper call sets on chr22 10-11Mb in the XBI dataset.
A variant is considered imputed if phasing Leave-on-out-r2 (L1or2) is greater than 0.5 and imputation info is greater than 0.8.

A)

Method	WES AF>0.01%	WES AF>0.1%
GATK	21,662 (1.81%)	8,973 (2.54%)
GraphTyper	5,310 (0.44%)	1,903 (0.54%)
GraphTyperHQ	16,774 (1.60%)	7,693 (2.17%)

B)

	Present in WES 200k		GATK		GraphTyper		GraphTyperHQ	
Type	WES AF>0.01%	WES AF>0.1%	WES AF>0.01%	WES AF>0.1%	WES AF>0.01%	WES AF>0.1%	WES AF>0.01%	WES AF>0.1%
A>C	71,587	24,700	1,948	824	580	166	1,511	643
A>G	380,627	127,772	6,260	2,740	1,681	665	5,600	2,650
A>T	44,040	15,489	1,368	620	357	126	908	397
C>G	101,848	34,675	2,640	1,085	706	242	2,097	941
C>T	377,729	126,438	7,649	2,963	1,462	526	5,188	2,425
G>T	71,556	24,815	1,797	741	524	178	1,470	637
Ti/Tv	2.62	2.55	1.79	1.74	1.45	1.67	1.80	1.94

Supplementary Table 5 Comparison of SNP and Indel call sets to WES.
A) Number of variants in the WES 200k dataset that are missing from GATK, GraphTyper and GraphTyperHQ datasets, conditioned on the frequency in WES 200k. The fractions of missing variants are inside the parenthesis. B) Total number of SNP types present in WES 200K conditioned on frequency and how many of those are missing from our WGS datasets, stratified by variant type. Ti = number of transitions, Tv = number of transversions.

Mutation type	Mutations Autosomes	Mutations ChrX	Opportunities Autosomes	Opportunities ChrX	% Total	% Autosomes	% ChrX
C>A	60,519,838	2,659,969	1,077,457,583	56,309,185	5.57%	5.62%	4.72%
C>G	57,676,447	2,854,929	1,077,457,583	56,309,185	5.34%	5.35%	5.07%
C>T	144,136,629	6,328,598	1,025,477,941	54,075,891	13.94%	14.06%	11.70%
CpG>TpG	42,363,944	1,843,388	51,979,642	2,233,294	81.54%	81.50%	82.54%
T>A	43,430,412	1,907,408	1,555,084,506	87,170,953	2.76%	2.79%	2.19%
T>C	159,740,935	6,892,088	1,555,084,506	87,170,953	10.15%	10.27%	7.91%
T>G	47,169,431	2,098,996	1,555,084,506	87,170,953	3.00%	3.03%	2.41%

Supplementary Table 6 Mutation saturation, results presented for autosomes and chrX separately.
Table shows the number of observed mutations in the GraphTyperHQ dataset and the number of possible mutation opportunities in regions of the genome amenable to short read sequence analysis.

A)

Method	Num variants	Missing call rate	Informative calls
GATK	710,913,648	2.57%	103,979,678,355,013
GraphTyper	655,928,639	0.14%	98,332,325,114,654
GraphTyperHQ	643,747,446	0.07%	96,570,956,991,770

B)

Method	SNPs	Transitions (Ti)	Transversions (Tv)	Ti/Tv
GATK	618,290,855	375,860,520	242,430,335	1.550
GraphTyper	593,953,779	369,120,364	224,833,415	1.642
GraphTyperHQ	585,040,410	364,859,729	220,180,681	1.657

C)

Method	Common	%	Rare	%	Singleton	%
GATK	31,501,254	(4.4%)	367,745,957	(51.7%)	311,666,437	(43.9%)
SNP	23,275,707	(3.8%)	317,087,938	(51.3%)	277,927,210	(44.9%)
Non-SNP	8,225,547	(8.9%)	50,658,019	(54.7%)	33,739,227	(36.4%)
GraphTyper	26,445,377	(4.0%)	335,241,409	(51.1%)	294,241,853	(44.9%)
SNP	20,261,132	(3.4%)	303,621,290	(51.1%)	270,071,357	(45.5%)
Non-SNP	6,184,245	(10.0%)	31,620,119	(51.0%)	24,170,496	(39.0%)
GraphTyperHQ	22,975,922	(3.6%)	327,718,095	(50.9%)	293,053,429	(45.5%)
SNP	18,124,082	(3.1%)	297,709,581	(50.9%)	269,206,747	(46.0%)
Non-SNP	4,851,840	(8.3%)	30,008,514	(51.1%)	23,846,682	(40.6%)

Supplementary Table 7 SNP and Indel call set summary
A) Number of variants in GATK, GraphTyper and GraphTyperHQ dataset. B) Variants split by transitions and transversions. C) Common = variants with frequency > 0.1%, rare = carried by more than one individual and frequency < 0.1%, singleton = carried by a single individual.

**Description**	**Beta**	**R**	**R^2**	***P*-value**
Autosomal dominant genes from OMIM	-0.0407	-0.0515	0.00265	6.60E-12
Recessive genes from OMIM	-0.0063	-0.0099	9.90E-05	0.185
Cell essential genes	0.0259	0.0497	0.00247	8.26E-10
Present in Cell essential genes	-0.0907	-0.1624	0.02636	4.31E-105
Hand curated list of Human lethal KO genes	-0.0204	-0.0141	0.0002	0.0627
Hand curated list (more permissive) of Human lethal KO genes	-0.0221	-0.0200	0.0004	0.0074
List of lethal KO genes in mice	-0.0425	-0.0877	0.0077	1.07E-31
List of lethal het. KO genes in mice	-0.0275	-0.0130	0.00017	0.0824

Supplementary Table 8 Regression of average DR overlapping gene exons on annotations from Gene discovery informatics toolkit[33].

A)

Data set	DR score	GERP RS score	CADD score	Eigen score	LINSIGHT score	CDTS
DR score	1	0.005	0.042	0.035	0.012	0.161
GERP RS score	0.005	1	0.535	0.247	0.352	0.005
CADD score	0.042	0.535	1	0.398	0.345	0.035
Eigen score	0.035	0.247	0.398	1	0.504	0.039
LINSIGHT score	0.012	0.352	0.345	0.504	1	0.013
CDTS	0.161	0.005	0.035	0.039	0.013	1

B)

Data set	DR score	GERP RS score	CADD score	Eigen score	LINSIGHT score	CDTS
DR score	1.00000	0.00003	0.00176	0.00123	0.00014	0.02592
GERP RS score	0.00003	1.00000	0.28623	0.06101	0.12390	0.00003
CADD score	0.00176	0.28623	1.00000	0.15840	0.11903	0.00123
Eigen score	0.00123	0.06101	0.15840	1.00000	0.25402	0.00152
LINSIGHT score	0.00014	0.12390	0.11903	0.25402	1.00000	0.00017
CDTS	0.02592	0.00003	0.00123	0.00152	0.00017	1.00000

Supplementary Table 9 a) Pearson correlation coefficient and b) r^2 between DR score and measures of sequence constraint and functional impact, computed over all autosomal chromosomes.

For each one of the 500bp overlapping windows in which the DR score (dr) is defined we compute the average value of the published scores (ps) in that window and then conduct linear regression analysis ($\mathrm{ps} \sim \mathrm{dr}$). The values shown in the table are the squared correlation coefficients of that regression. The correlation between the published datasets is computed from a set of 50bp non-overlapping windows using the average score within each window. A similar regression is conducted between each of the published datasets to obtain the squared correlation coefficient. Note, that the p-value for the linear regression fit is below computational threshold (2.2×10^{-308}) for each pair of data sets in the table. CADD, Eigen and LINSIGHT all incorporate GERP into their annotation and are consequently not independent of each other or GERP. DR score and CDTS employ an analogous methodology, but scores are derived independently of each other and the other metrics.

Cohort	Chip N	WGS N	WGS %
XBI	431,805	132,169	30.6
XAF	9,633	2,963	30.8
XSA	9,252	3,047	32.8
OTH	37,598	11,781	31.9

Supplementary Table 10 Number of individuals in the three cohorts described in this study.

Threshold % XBI	Threshold % XAF,XSA		XBI Snp/Indel	XBI SV	XBI MSat	XAF Snp/Indel	XAF SV	XAF MSat	XSA Snp/Indel	XSA SV	XSA MSat
1%	5%	Phased	11189434	15569	2491240	10782733	15214	2388595	7941383	11773	1812461
		Imputed	11184312	15518	2488009	10728154	14606	2354675	7865444	11211	1743407
		n	11297050	18044	2600902	10864088	17276	2453893	7993858	13415	1859421
0.1%	1%	Phased	6590616	7234	1068743	8365507	9301	1166814	3739563	4230	816116
		Imputed	6586277	7223	1066743	8315664	9140	1129348	3633830	4072	699673
		n	6819668	9185	1329131	8555777	11074	1310478	3852601	5235	896908
0.01%	0.5%	Phased	23598990	24317	1581904	3950291	4139	391700	2122454	2168	369854
		Imputed	23453107	24037	1558812	3914244	4077	369608	2008801	2062	271659
		n	24556101	31246	2330992	4114602	5187	504462	2280485	2808	442328
0.005%	0.2%	Phased	19864378	19181	482916	4386799	4263	354516	2642260	2558	380537
		Imputed	19440299	18735	457453	4316711	4136	319739	2409667	2297	235403
		n	21059670	25103	850280	4722651	5635	515106	2982169	3624	488799
0.002%	0.1%	Phased	43902487	41664	600207	6892163	6336	448483	5032021	4717	539656
		Imputed	41679009	39448	542137	6627483	6041	366561	4292840	3964	260599
		n	50063971	55664	1214690	8424367	9507	772036	6418472	7497	786098
0.001%	0.04%	Phased	52975884	49438	437379	6495546	5681	337279	5944556	5125	428185
		Imputed	47238234	44171	363952	5861702	5106	240464	4635202	3933	162809
		n	72522701	74342	1092057	10462313	10807	713472	9539163	10093	745160
0.0002%	0.008%	Phased	40518700	36567	292304	16233640	12769	569083	12625599	8801	715628
		Imputed	31988313	29453	189966	12109642	10130	321072	6563488	4830	190230
		n	263633284	261011	1935535	59096600	52531	1654282	52146463	47256	1671469

Supplementary Table 11 Imputation and phasing accuracy as a function of frequency within each cohort.
Phased refers to number of variants with Leave-one-out-r2 value > 0.5 and imputed refers to phased variants that also have imputation info > 0.8. Numbers are for variants at frequency above the given threshold and not included in frequency thresholds in earlier lines, e.g., in the XBI population 72,522,701 variants have frequency between 0.001 and 0.002%, of which 52,975,884 could be phased and 47,238,234 could be imputed.

AF Threshold		XBI		XAF		XSA	
	**Panel**	**n**	**present %**	**n**	**present %**	**n**	**present %**
≥10^{-2}	Bycroft	9,675,179	57.1%	9,049,185	54.4%	8,782,729	55.4%
	150k WGS	16,838,810	99.3%	16,500,186	99.3%	15,728,295	99.1%
	Both	9,555,642	56.3%	8,924,920	53.7%	8,645,958	54.5%
	Either	16,958,347	100%	16,624,451	100%	15,865,066	100%
≥10^{-3}	Bycroft	5,150,551	40.8%	4,321,491	37.0%	1,509,037	23.8%
< 10^{-2}	150k WGS	12,497,109	99.1%	11,609,254	99.3%	6,276,519	98.8%
	Both	5,031,517	39.9%	4,236,985	36.2%	1,432,690	22.6%
	Either	12,616,143	100%	11,693,760	100%	6,352,866	100%
≥ 10^{-4}	Bycroft	4,635,660	12.7%	7,894,440	34.0%	1,637,838	17.8%
< 10^{-3}	150k WGS	36,247,790	99.1%	22,801,909	98.2%	8,903,892	96.5%
	Both	4,299,464	11.8%	7,474,332	32.2%	1,315,077	14.3%
	Either	36,583,986	100%	23,222,017	100%	9,226,653	100%
< 10^{-4}	Bycroft	1,786,117	0.9%	4,951,605	8.8%	2,001,548	5.3%
	150k WGS	196,375,197	99.6%	54,623,218	97.1%	37,019,802	97.4%
	Both	942,249	0.5%	3,315,555	5.9%	1,024,799	2.7%
	Either	197,219,065	100%	56,259,268	100%	37,996,551	100%

Supplementary Table 12 Number of markers that impute (Imp Info > .8) in 500k set of UKB using the imputation panel presented here (150k WGS) and an imputation by Bycroft et al.[5]. Both represents number of markers imputed by both panels, either the number of markers in either panel.

# repeats	Frequency	Effect	*P*-value
4.7	5.98E-05	0.02	8.84E-01
5.7	3.64E-01	0.41	3.95E-07
6	1.45E-06	0.02	9.84E-01
6.7	9.99E-04	4.96	2.16E-01
7.7	4.16E-04	0.02	6.94E-01
8.7	7.46E-03	0.69	6.95E-01
9.7	1.21E-03	4.27	2.54E-01
10.7	9.27E-03	0.54	5.10E-01
11.7	1.20E-01	1.45	7.33E-02
12	1.95E-06	0.02	9.81E-01
12.7	1.24E-01	0.85	4.98E-01
13	1.17E-06	0.02	9.87E-01
13.7	1.78E-01	0.77	2.08E-01
14.7	6.30E-02	0.82	5.26E-01
15.7	8.18E-03	0.73	7.43E-01
16.7	9.16E-03	0.01	7.66E-02
17.7	4.88E-03	1.06	9.54E-01
18.7	2.15E-03	0.02	3.72E-01
19.7	4.39E-03	1.44	7.34E-01
20.7	1.81E-02	2.02	1.32E-01
21.7	2.69E-02	1.12	8.27E-01
22.7	1.41E-02	2.4	1.38E-01
23.7	8.77E-03	2.18	3.15E-01
24.7	6.90E-03	2.44	2.59E-01
25.7	7.05E-03	4.06	4.74E-02
26.7	5.17E-03	6.62	1.40E-02
27.7	4.55E-03	9.82	1.50E-03
28.7	3.38E-03	17.93	1.24E-04
29.7	2.33E-03	19.75	4.08E-04
30.7	1.55E-03	30.02	6.02E-05
31.7	1.03E-03	48.35	4.33E-09
32.7	6.98E-04	42.04	1.30E-04
33.7	4.04E-04	74.03	8.07E-06
34.7	3.05E-04	68.27	5.01E-05
35.7	1.48E-04	141.58	1.29E-10
36.7	1.50E-04	45.23	3.35E-02
37.7	9.60E-05	51.68	2.49E-01
38.7	1.04E-04	92.19	3.64E-03
>=39.7	4.32E-05	161.74	1.09E-07

Supplementary Table 13 Association of number of repeat copies of microsatellite in 3’ UTR in DMPK with myotonic dystrophy.
Individuals carrying 39.7 or more copies of the repeat are grouped together by popSTR[55]. P-values are computed using a two-sided χ^2-test.

A)

Gene	Number of Allelic variants in OMIM	OMIM Phenotype with allelic variants * (Mode of inheritance) **
ALB	61	Analbuminemia (AR)
CACNA1A	37	Episodic ataxia, type 2 (AD) ; Migraine, familial hemiplegic, 1 (AD); Epileptic encephalopathy, early infantile, 42 (AD); Spinocerebellar ataxia 6 (AD)
HBB	540	Delta-beta thalassemia(AD); Erythrocytosis 6 (AD) ; Heinz body anemia (AD); Hereditary persistence of fetal hemoglobin (AD); Methemoglobinemia, beta type(AD); Sickle cell anemia (AR); Thalassemia-beta, dominant inclusion-body (AD)
PCSK9	8	Hypercholesterolemia, familial, 3 (AD)
PIEZO1	16	Dehydrated hereditary stomatocytosis(AD); Lymphedema, hereditary, III (AR)
GHRH	0	None
DMPK	1	Myotonic dystrophy 1 (AD)
GCSH	1	None
TAC3	2	Hypogonadotropic hypogonadism 10 with or without anosmia (AR)
NMRK2	0	None

B)

Gene	N Drug ***	Indications ***	Link
ALB	None		
CACNA1A	5	7	https://platform.opentargets.org/target/ENSG00000141837
HBB	3	11	https://platform.opentargets.org/target/ENSG00000244734
PCSK9	6	28	https://platform.opentargets.org/target/ENSG00000169174
PIEZO1	None		
GHRH	None		
DMPK	None		
GCSH	None		
TAC3	None		
NMRK2	None		

Supplementary Table 14 Information on genes presented.
A) Phenotypes and allelic variants in OMIM for selected genes. B) Known drug data and in open targets for selected targets. *Excluding the ones with provisional phenotype gene relationship "?"; multifactorial diseases"{ }" and non diseases"[ ]" ** Mode of inheritance : AD Autosomal dominant; AR Autosomal recessive. ***Known drug data according to Open Targets[72].

Phenotype	Data showcase field	Extra information
Age at menopause	3581	Adjusted for year of birth and 20 principal components, then inverse-normal transformed
Age of menarche	2714	Adjusted for year of birth and 20 principal components, then inverse-normal transformed
Albumin	30600	Adjusted for age, age² and 20 principal components, then combined and inverse-normal transformed
Calcium	30680	Adjusted for age, age² and 20 principal components, then combined and inverse-normal transformed
Glycine	23462	Metabolomics
Height	50	Adjusted for year of birth, sex and 20 principal components for males and females separately, then combined and inverse-normal transformed
Hemoglobin concentration, Asian ancestry	30060	Adjusted for age, age² and 45 principal components for males and females separately, then combined and inverse-normal transformed
IGF-1 serum levels	30770	Adjusted for age, age² and 20 principal components
Mean corpuscular volume	30040	Adjusted for age, age² and 20 principal components for males and females separately, then combined and inverse-normal transformed
Non-HDL cholesterol, European ancestry	Field 30690 minus field 30670 (HDL)	Adjusted for age, age² and 20 principal components; lipid-lowering drug users had their measurements divided by 0.8, then combined and inverse-normal transformed
Non-HDL cholesterol, African ancestry	Field 30690 minus field 30670 (HDL)	Adjusted for age, age² and 20 principal components; lipid-lowering drug users had their measurements divided by 0.8, then combined and inverse-normal transformed
Total cholesterol	30690	Adjusted for age, age² and 20 principal components; lipid-lowering drug users had their measurements divided by 0.8, then combined and inverse-normal transformed
Uric acid	30880	Adjusted for age, age² and 20 principal components, then combined and inverse-normal transformed
Gout	ICD-19 code M10* on fields 41270, 41271 and 42040	Adjusted for year of birth, sex and 20 principal components
Hereditary ataxia	ICD-10 code G11 on fields 41270, 41271 and 42040	Adjusted for year of birth, sex and 20 principal components
Myotonic dystrophy	ICD-10 code G71.1 on fields 41270, 41271 and 42040	Adjusted for year of birth, sex and 20 principal components

Supplementary Table 15 Phenotypes used in this study, their field in the UKB data showcase and adjustments performed prior to association analysis

Parameter	Information Requested	Definition
prc_auto_ge_15x	Coverage	PCT_15X from .wgsmetrics_autosome in QCPreview
coverage	autosomal mean coverage	MEAN_COVERAGE * (1.0 - PCT_EXC_DUPE - PCT_EXC_OVERLAP - PCT_EXC_ADAPTER) / (1.0 - PCT_EXC_TOTAL) from .wgsmetrics_autosome in QCPreview
genetic_sex	Sex	if NX<=0.3 then "Female" else if NX>=0.7 then "Male" else "Undetermined" from .sexcheck output file in QCStats
yield	Yield	GENOME_TERRITORY * MEAN_COVERAGE * (1.0 - PCT_EXC_DUPE - PCT_EXC_OVERLAP - PCT_EXC_ADAPTER) / (1.0 - PCT_EXC_TOTAL) from .wgsmetrics output file in QCPreview
read_haps_error_percentage	Read_haps	100*DOUBLE_ERROR_FRACTION from .contamination output file in QCStats
freemix_percentage	Freemix/Verify Bam ID	100 * FREEMIX from .verifyBamId.selfSM output file in QCStats
prc_proper_pairs	Proportion of mapped read pairs	100 * (reads_properly_paired/reads_mapped) from .stats output file in QCPreview
discordance_prc	NRD Genotyping	100 * (1.0 - NON_REF_GENOTYPE_CONCORDANCE) from .genotype_concordance_summary_metrics in Concords or -1 if chip genotypes are not available

Supplementary Table 16 QA/QC metrics derived from the files delivered to the UKB. The result is written to a file, qaqc_metric.

Column	Min	Max	Flag	Explanation
SAMPLE_ID				Read group ID
LANE				Lane ID (=Read group ID)
FAILURE_FLAGS				Failure flag
JOINT_CALLING_FLAGS				Joint calling failure flag
STRICT_FLAGS				Strict failure flag
TOTAL_BPS	3e8	1e14	C	Total basepairs
TOTAL_READ_PAIRS				Total read pairs
READ_LENGTH				Read length
MEAN_BASE_QUAL_PER_READ	30	100	Q	Mean of base calling quality
STD_BASE_QUAL_PER_READ	-1	10	Q	Std dev of mean base calling quality
MEAN_N_COUNT_PER_READ	-1	10	N	Mean Percentage N
STD_N_COUNT_PER_READ	-1	30	N	Std dev of Percentage N
MEAN_GC_CONTENT_PER_READ	39	45	G	Mean percentage of GC bases
STD_GC_CONTENT_PER_READ	-1	15	G	Std dev of Percentage GC
MEAN_BASE_QUAL_PER_POSITION	30	100	Q	Mean of mean base calling quality
STD_BASE_QUAL_PER_POSITION	-1	6	Q	Std dev of mean base calling quality
MEAN_N_PER_POSITION	-1	10	N	Mean Percentage N
STD_N_PER_POSITION	-1	10	N	Std dev of Percentage N
MEAN_A_PER_POSITION	25	35	B	Mean Percentage A
STD_A_PER_POSITION	-1	10	B	Std dev of Percentage A
MEAN_C_PER_POSITION	15.5	25	B	Mean Percentage C
STD_C_PER_POSITION	-1	10	B	Std dev of Percentage C
MEAN_G_PER_POSITION	17	24	B	Mean Percentage G
STD_G_PER_POSITION	-1	10	B	Std dev of Percentage G
MEAN_T_PER_POSITION	25	33	B	Mean Percentage T
STD_T_PER_POSITION	-1	10	B	Std dev of Percentage T
32_MER_ERROR_RATE				Estimated 32-mer error rate
ADAPTER_8_MERS	-1	5	A	Percentage of Universal adapter 8-mers
MARKED_DUPLICATE	-1	60	D	Percentage marked as duplicate
UNMAPPED	-1	20	U	Percentage unmapped reads
BOTH_UNMAPPED	-1	30	U	Percentage both reads in pair unmapped
FIRST_UNMAPPED	-1	30	U	Percentage only first unmapped in pair
SECOND_UNMAPPED	-1	30	U	Percentage only second unmapped in pair
PROPER_PAIRS				Percentage proper pairs
PROPER_PAIRS_AUTOSOME	95	1000	P	Percentage proper pairs autosome
FF_RR_PAIRS	-1	0.1	o	Percentage FF/RR oriented pairs
MEAN_COVERAGE	0.1	100000	C	Mean coverage
STD_COVERAGE	-1	100000	C	Std dev of coverage
MEAN_INSERT_SIZE	-1	10000	I	Mean insert size
STD_INSERT_SIZE				Std dev of insert size
ADAPTER_INSERT_SIZE	-1	20	A	Percent insert size < read length
MAPPING_QUAL_60				Percentage reads with mapping quality <60
MAPPING_QUAL_40				Percentage reads with mapping quality <40
MAPPING_QUAL_20				Percentage reads with mapping quality <20
MEAN_MISMATCHES	-1	5	m	Mean mismatches per read pair
MEAN_DELETIONS				Mean deletions per read pair
MEAN_INSERTIONS				Mean insertions per read pair
NZ_DELETIONS	-1	0.1	d	Fraction or reads that have a deletion
NZ_INSERTIONS	-1	0.1	I	Fraction of reads that have an insertion
CLIPPED_5_PRIME	-1	6	c	Percentage of reads clipped at 5'-end
CLIPPED_3_PRIME	-1	30	c	Percentage of reads clipped at 3'-end
C>A	0.3	0.7	O	C>A triplet conversion rate
G>A	0.4	0.6	O	G>A triplet conversion rate
T>A	0.3	0.7	O	T>A triplet conversion rate
A>C	0.3	0.7	O	A>C triplet conversion rate
G>C	0.3	0.7	O	G>C triplet conversion rate
T>C	0.3	0.7	O	T>C triplet conversion rate

Supplementary Table 17 Metrics collected for each lane by bamqc_summary.
If any flag is raised, the lane is excluded from the merge process. The values, per read group, are collected in the file .bamqc_summary.

A)

Method	#Variants	Common (>0.1%)	Rare (<0.1%)	Singleton
GATK	6,221,575	284,303	3,259,421	2,677,851
GraphTyper	5,569,026	224,715	2,855,132	2,489,179

B)

Method	#Variants	SNPs	Non-SNPs
GATK	6,221,575	5,400,679	820,896
GraphTyper	5,569,026	5,040,466	528,560

C)

Method	Missing genotypes	#Informative calls
GATK	3.26%	903,536,315,740
GraphTyper	0.11%	835,097,232,768

D)

Method	Transitions (Ti)	Transversion (Tv)	Ti/Tv
GATK	3,246,174	2,154,505	1.507
GraphTyper	3,130,524	1,909,942	1.639

Supplementary Table 18 Results for 500 random test regions.
A) Number of variants called by GATK and GraphTyper conditioned on frequency class. B) Number of variants conditioned on variant type. C) Fraction of missing variant calls. D) Number of transitions and transversions.

A)

Method	Total common	Failed	%
GATK	284,303	21,234	7.47%
GraphTyper	224,715	2,277	1.01%

B)

	Failed count	
Test	GATK	GraphTyper
Sanger Vanguard vs. Sanger Main	13,440	999
Sanger Vanguard vs. deCODE	16,751	1,825
Sanger Main vs. deCODE	13,510	1,141

Supplementary Table 19 Number of common variants (frequency > .1%) that showed significant association with sequencing center in the 500 random regions test set.
A) Total number of variants that failed in any test. B) Number of failed variants stratified by sequencing protocol. Variant is considered "Failed" if p-value < 1e-6, Fisher's exact test.

A)

Method	Common	SaM vs. deC	SaV vs. deC	SaV vs. SaM
GATK	31,501,254	1,202,575	1,164,682	810,105
GraphTyper	26,445,377	166,371	175,144	66,838
GraphTyperHQ	22,975,922	28,432	36,283	8,096

B)

Method	Common	Any test $p < 10^{-6}$	Any test $p < 10^{-10}$
GATK	31,501,254	1,792,003 (5.69%)	1,197,839 (3.80%)
GraphTyper	26,445,377	257,860 (0.97%)	136,521 (0.52%)
GraphTyperHQ	22,975,922	46,556 (0.20%)	22,307 (0.10%)

Supplementary Table 20 Number of common variants (frequency > 0.1%) that show significant association to sequencing center, indicating batch effects.
Computed using a Fisher's exact test, for common (> 0.1% frequency) variants. A) Number of failed variants stratified by test using $p < 10^{-6}$. deC = samples sequenced at deCODE genetics. SaV = samples sequenced using the Sanger Vanguard processing pipeline. SaM = samples sequenced using the Sanger main phase pipeline. B) Total number of variants that failed in any test, using both $p < 10^{-6}$ and $p < 10^{-10}$.

Dataset	Shared with both other	Specific to	Absent from	Absent from and same carrier in both other datasets	Fraction of missing variants with same carrier in both datasets
GATK	6,608,669	230,808	15,567	12,700	81.58%
GraphTyperHQ	6,608,669	54,909	87,773	56,052	63.86%
WES200k	6,608,669	28,039	498,181	476,195	95.59%

Supplementary Table 21 Three-way comparison between the GraphTyperHQ, GATK and WES200k[59] call analyzed inside WES capture regions within the set of 109,618 individuals present in both the WES200k call set an our set of 150,119 individuals.

XBI	*P*-value	MaF > 0.01	MaF 0.01 - 0.001	MaF < 0.001
Unfiltered	> 0.05	17.0-17.5M (89-91%)	9.27-9.33M (93-94%)	208-215M (94-97%)
	0.005-0.05	1.05-1.13M (5.5-5.9%)	481-497K (4.9-5.0%)	5.60-12.4M (2.5-5.6%)
	5e-4 - 0.005	238-258K (1.2-1.3%)	64.1-77.8K (0.65-0.78%)	443-848K (0.2-0.38%)
	5e-8 - 5e-4	214-324K (1.1-1.7%)	28.9-49.9K (0.29-0.5%)	36.8-65.6K (0.017-0.03%)
	< 5e-8	127-540K (0.66-2.8%)	7.74-49.2K (0.078-0.5%)	364-3697 (0.00016-0.0017%)
Filtered	> 0.05	16.0-16.1M (94-94%)	8.94-8.96M (94-95%)	207-214M (94-97%)
	0.005-0.05	808-839K (4.7-4.9%)	435-445K (4.6-4.7%)	5.57-12.3M (2.5-5.6%)
	5e-4 - 0.005	103-122K (0.6-0.72%)	46.9-55.5K (0.5-0.59%)	439-840K (0.2-0.38%)
	5e-8 - 5e-4	36.1-78.4K (0.21-0.46%)	10.1-16.7K (0.11-0.18%)	36.1-60.7K (0.016-0.028%)
	< 5e-8	11.2-68.9K (0.066-0.4%)	2.37-11.5K (0.025-0.12%)	115-463 (5.2e-05-0.00021%)
**XAF**	***P*-value**	**MaF > 0.01**	**MaF 0.01 - 0.001**	**MaF < 0.001**
Unfiltered	> 0.05	29.7-29.9M (94-95%)	22.5-22.9M (93-95%)	80.8-84.6M (95-99%)
	0.005-0.05	1.43-1.48M (4.5-4.7%)	1.04-1.44M (4.3-6.0%)	0.717-4.41M (0.84-5.2%)
	5e-4 - 0.005	152-189K (0.48-0.6%)	79.6-143K (0.33-0.59%)	10.6-118K (0.012-0.14%)
	5e-8 - 5e-4	20.4-73.6K (0.065-0.23%)	6.87-18.2K (0.029-0.076%)	1-5392 (1.2e-06-0.0063%)
	< 5e-8	732-29023 (0.0023-0.092%)	62-335 (0.00026-0.0014%)	0-1 (0.0-1.2e-06%)
Filtered	> 0.05	27.4-27.4M (95-95%)	21.8-22.2M (93-95%)	80.1-83.9M (95-99%)
	0.005-0.05	1.26-1.30M (4.4-4.5%)	0.994-1.39M (4.3-6.0%)	0.709-4.38M (0.84-5.2%)
	5e-4 - 0.005	127-133K (0.44-0.46%)	75.7-135K (0.33-0.58%)	10.5-117K (0.012-0.14%)
	5e-8 - 5e-4	13.4-23.8K (0.046-0.083%)	6.36-15.3K (0.027-0.066%)	1-5294 (1.2e-06-0.0063%)
	< 5e-8	28-5752 (9.7e-05-0.02%)	0-166 (0.0-0.00071%)	0-0 (0.0-0.0%)
**XSA**	***P*-value**	**MaF > 0.01**	**MaF 0.01 - 0.001**	**MaF < 0.001**
Unfiltered	> 0.05	18.9-19.1M (94-95%)	14.1-14.5M (94-96%)	73.9-76.5M (95-99%)
	0.005-0.05	919-989K (4.6-4.9%)	521-817K (3.5-5.4%)	1.00-3.58M (1.3-4.6%)
	5e-4 - 0.005	99.7-142K (0.5-0.71%)	32.1-92.3K (0.21-0.61%)	17.3-83.7K (0.022-0.11%)
	5e-8 - 5e-4	13.2-67.8K (0.066-0.34%)	2.97-12.7K (0.02-0.085%)	358-3980 (0.00046-0.0051%)
	< 5e-8	665-30416 (0.0033-0.15%)	92-278 (0.00061-0.0018%)	0-2 (0.0-2.6e-06%)
Filtered	> 0.05	17.0-17.0M (95-95%)	13.6-13.9M (94-96%)	73.3-75.9M (95-99%)
	0.005-0.05	796-809K (4.4-4.5%)	494-778K (3.4-5.4%)	0.994-3.56M (1.3-4.6%)
	5e-4 - 0.005	82.4-87.9K (0.46-0.49%)	30.6-85.5K (0.21-0.59%)	17.1-82.8K (0.022-0.11%)
	5e-8 - 5e-4	9.29-15.8K (0.052-0.088%)	2.75-10.1K (0.019-0.07%)	331-3865 (0.00043-0.005%)
	< 5e-8	16-4327 (8.9e-05-0.024%)	1-142 (6.9e-06-0.00098%)	0-0 (0.0-0.0%)

Supplementary Table 22 Batch effects for sequencing center in the raw genotype calls.
Six phenotypes for batch effects are tested. Results are conditioned on marker minor allele frequency (MAF). Table shows the minimum and maximum number and fraction of markers, across the six phenotypes) with p-value in each p-value range. E.g., when considering the unfiltered dataset and the XSA cohort, MAF > 0.01, between 919 and 989k markers have p-value between 0.005 and 0.05, corresponding to 4.6-4.9% of markers with MAF > 0.01. P-values are computed using a two-sided χ2-test.

XBI	*P*-value	MaF > 0.01	MaF 0.01 - 0.001	MaF < 0.001
Unfiltered	> 0.05	16.8-17.1M (92-94%)	9.47-9.55M (94-95%)	254-266M (93-98%)
	0.005-0.05	887-910K (4.9-5.0%)	472-495K (4.7-4.9%)	6.30-17.3M (2.3-6.3%)
	5e-4 - 0.005	113-155K (0.62-0.85%)	54.4-71.4K (0.54-0.71%)	466-942K (0.17-0.35%)
	5e-8 - 5e-4	40.2-137K (0.22-0.75%)	10.4-40.5K (0.1-0.4%)	38.5-74.5K (0.014-0.027%)
	< 5e-8	15.9-180K (0.087-0.99%)	925-27218 (0.0092-0.27%)	85-1389 (3.1e-05-0.00051%)
Filtered	> 0.05	15.4-15.4M (95-95%)	8.78-8.79M (95-95%)	216-225M (93-97%)
	0.005-0.05	733-738K (4.5-4.5%)	418-421K (4.5-4.6%)	5.63-14.7M (2.4-6.4%)
	5e-4 - 0.005	72.5-82.8K (0.45-0.51%)	42.1-44.9K (0.46-0.49%)	425-848K (0.18-0.37%)
	5e-8 - 5e-4	8.07-24.2K (0.05-0.15%)	4.74-6.31K (0.051-0.068%)	35.4-64.2K (0.015-0.028%)
	< 5e-8	117-11166 (0.00072-0.069%)	0-592 (0.0-0.0064%)	0-7 (0.0-3e-06%)
**XAF**	***P*-value**	**MaF > 0.01**	**MaF 0.01 - 0.001**	**MaF < 0.001**
Unfiltered	> 0.05	27.9-28.0M (95-95%)	19.6-19.8M (94-95%)	77.1-79.6M (96-99%)
	0.005-0.05	1.30-1.34M (4.4-4.5%)	0.950-1.13M (4.6-5.4%)	0.670-3.04M (0.84-3.8%)
	5e-4 - 0.005	132-148K (0.45-0.5%)	72.6-124K (0.35-0.59%)	16.1-107K (0.02-0.13%)
	5e-8 - 5e-4	13.8-32.8K (0.047-0.11%)	5.97-14.3K (0.029-0.068%)	300-5385 (0.00037-0.0067%)
	< 5e-8	92-5922 (0.00031-0.02%)	36-136 (0.00017-0.00065%)	0-3 (0.0-3.7e-06%)
Filtered	> 0.05	25.3-25.3M (95-95%)	17.6-17.8M (94-95%)	60.3-62.2M (96-99%)
	0.005-0.05	1.16-1.19M (4.4-4.5%)	856-996K (4.6-5.3%)	0.556-2.42M (0.89-3.9%)
	5e-4 - 0.005	110-118K (0.41-0.44%)	64.7-106K (0.35-0.57%)	13.9-85.6K (0.022-0.14%)
	5e-8 - 5e-4	11.6-13.5K (0.043-0.051%)	5.14-10.9K (0.027-0.058%)	258-4427 (0.00041-0.0071%)
	< 5e-8	1-104 (3.8e-06-0.00039%)	0-1 (0.0-5.3e-06%)	0-0 (0.0-0.0%)
**XSA**	***P*-value**	**MaF > 0.01**	**MaF 0.01 - 0.001**	**MaF < 0.001**
Unfiltered	> 0.05	17.7-17.8M (95-95%)	13.8-14.1M (94-96%)	67.2-68.7M (97-99%)
	0.005-0.05	836-876K (4.5-4.7%)	506-780K (3.5-5.3%)	0.674-2.14M (0.97-3.1%)
	5e-4 - 0.005	84.3-103K (0.45-0.55%)	33.4-84.6K (0.23-0.58%)	14.8-71.5K (0.021-0.1%)
	5e-8 - 5e-4	9.70-26.1K (0.052-0.14%)	2.83-10.0K (0.019-0.068%)	531-3555 (0.00077-0.0051%)
	< 5e-8	83-6253 (0.00044-0.033%)	26-94 (0.00018-0.00064%)	0-11 (0.0-1.6e-05%)
Filtered	> 0.05	15.6-15.6M (95-95%)	10.8-11.0M (94-96%)	40.0-40.9M (97-99%)
	0.005-0.05	718-736K (4.4-4.5%)	412-603K (3.6-5.3%)	0.478-1.38M (1.2-3.3%)
	5e-4 - 0.005	71.8-76.5K (0.44-0.47%)	25.4-65.0K (0.22-0.57%)	11.0-49.4K (0.027-0.12%)
	5e-8 - 5e-4	8.02-9.39K (0.049-0.057%)	2.14-6.70K (0.019-0.058%)	394-2561 (0.00095-0.0062%)
	< 5e-8	0-47 (0.0-0.00029%)	0-0 (0.0-0.0%)	0-0 (0.0-0.0%)

Supplementary Table 23 Batch effects for sequencing center in the imputed genotype calls. Six phenotypes for batch effects are tested. Results are conditioned on marker minor allele frequency (MAF). Table shows the minimum and maximum number and fraction of markers, across the six phenotypes) with p-value in each p-value range. E.g., when considering the unfiltered dataset and the XSA cohort, MAF > 0.01, between 836 and 876k markers have p-value between 0.005 and 0.05, corresponding to 4.5-4.7% of markers with MAF > 0.01. P-values are computed using a two-sided χ^2-test.

Phenotype	LD score intercept	Mean χ^2 unadj	λ unadj	λ unadj maf<0.01	Attenuation ratio	Method	Marker
Age at menopause	1.051	1.463	1.048	1.005	0.110	BOLT-LMM	chr19:3939254
Age of menarche	1.095	2.081	1.048	1.028	0.088	BOLT-LMM	chr12:57010289
Albumin	1.236	2.028	1.094	1.017	0.229	BOLT-LMM	chr4:73399955
Calcium	1.166	1.985	1.078	1.011	0.169	BOLT-LMM	chr4:73399955
Glycine	0.976	1.457	1.016	0.983	-0.053	BOLT-LMM	chr16:81069345
Height	1.825	5.222	1.150	1.107	0.195	BOLT-LMM	chr20:37261871
Hemoglobin concentration, Asian ancestry	1.008	1.015	1.001	0.998	0.574	Linear regression	chr16:88716656
IGF-1 serum levels	1.320	2.995	1.079	1.053	0.160	BOLT-LMM	chr20:37261871
Mean corpuscular volume	1.215	1.896	1.033	1.018	0.240	BOLT-LMM	chr11:5225486
Non-HDL cholesterol, European ancestry	1.786	2.465	1.082	1.010	0.537	BOLT-LMM	chr1:55029214
Non-HDL cholesterol, African ancestry	1.000	1.005	1.005	1.004	0.072	Linear regression	chr1:55063542
Total cholesterol	1.739	2.568	1.082	1.009	0.471	BOLT-LMM	chr4:73399955
Uric acid	0.803	4.198	1.059	1.036	-0.062	BOLT-LMM	chr1:125079549, chr1:121062032
Gout	1.008	1.336	0.847	0.838	0.024	Logistic regression	chr1:125079549, chr1:121062032
Hereditary ataxia	1.019	1.017	0.262	0.154	1.142	Logistic regression	chr19:13207859
Myotonic dystrophy	1.050	1.036	0.119	0.053	1.408	Logistic regression	chr19:45770205

Supplementary Table 24 Correction factors and inflation metrics from phenotypes used in this study.
LD score intercept, mean chi-squared unadjusted value, unadjusted lambda value, unadjusted lambda value for rare (< 1% MAF) markers and attenuation ratio. Marker represents the ID of the association reported.

Marker	R^2 imp vs raw	SaM vs. others	SaM vs. SaV	SaV vs. others	deCODE vs. Sa	deC vs. SanM	deC vs. SaV
chr1:55063542	0.997	0.098	0.294	0.746	0.211	0.1120	0.9887
chr19:13207859	0.995	0.176	0.317	0.496	0.390	0.2105	0.6639
chr19:45770205	0.879	0.292	0.583	0.731	0.394	0.3174	0.8304
chr11:5225486	1.000	0.436	0.984	0.730	0.349	0.3726	0.6142
chr12:57010289	1.000	0.429	0.006	0.006	0.634	0.7400	0.0090
chr1:121062032	0.997	0.060	0.413	0.896	0.080	0.0563	0.8189
chr1:125079549	0.998	0.103	0.317	0.620	0.186	0.1133	0.8276
chr20:3726187	0.995	0.682	0.116	0.133	0.714	0.897	0.1720
chr19:3939254	0.999	0.811	0.653	0.484	0.556	0.707	0.4582
chr1:55029214	1.000	0.352	0.091	0.042	0.092	0.235	0.0318
chr4:73399955	1.000	0.547	0.815	0.624	0.407	0.479	0.5579
chr16:88716656	0.995	0.057	0.031	0.059	0.460	0.113	0.1034
chr16:81069345	1.000	0.012	0.245	0.907	0.023	0.012	0.735

Supplementary Table 25 R^2 between raw genotypes and imputed markers in the XBI cohort. p-value for batch effect in the XBI cohort for markers presented in this study. deC = samples sequenced at deCODE genetics. SaV = samples sequenced using the Sanger Vanguard processing pipeline. SaM = samples sequenced using the Sanger main phase pipeline. Sa = samples sequenced at Sanger. Relationship between marker IDs and phenotypes can be seen in Supplementary Table 24.

Supplementary Notes

Supplementary Note 1: WGS data quality specification.

Sequencing was performed at the two sequencing providers, deCODE genetics and the Wellcome Trust Sanger Institute, according to the specifications set forth in the material transfer agreement for UKB Access application nr. 52293 – Summarized as follows:

QC parameter	Sample level	Batch level
Sequencer type	Illumina NovaSeq6000 or better with standard 151 base, parired-end chemistry	
Sequencing library	PCR-free, uniquely dual-indexed in multiplexed pools	
Read-length	>100bp	
Proper-pairs	% of mapped read-pairs from the same DNA fragment with appropriate orientation and separation:<br>≥95% PASS<br><95% FAIL	
Coverage	% of autosome covered ≥15x:<br>≥95% PASS<br><95% FAIL	The mean sample genome coverage across the monthly sequencing batch is expected to be approximately 30X across the genome with a minimum coverage of 26X.
Contamination level 1<br>(Freemix)	Freemix sample contamination level as measured by VerifyBamID[77]:<br>≥5% FAIL<br>>1% and <5% further analyzed with Read_haps[78]<br><1% PASS	≤4 samples per 96 sample sequencing plate<br>≤1% per monthly sequencing batch
Contamination level 2<br>(Read_haps)	For samples with Freemix values 1-5%, contamination is verified by Read_haps	
Sample Identity Concordance	Discordance at non-reference genotypes ≥2% FAIL<br><2% PASS	Sample identity concordance failures within each monthly sequencing batch must be <0.05%
Montly seq batch overall failure rate		Repeat Sample requests are no more than 1% of the monthly sequencing batch

All calculations of data quantity (yield) and coverage must exclude duplicate reads, adaptors, overlapping bases from reads from the same fragment, soft-clipped bases

Supplementary Note 2: Whole genome sequencing

DNA samples were selected by UK Biobank using its picking algorithm which ensures pseudo-randomisation of recruitment centres and collection times across batches, to avoid potential batch effects and shipped on dry-ice to the sequencing centers at Welcome Sanger Institute in Cambridgeshire, UK (WSI) and deCODE genetics in Reykjavik, Iceland (deCODE). The samples were in 70 µL aliquots in Fluid-X 0.3 mL, externally threaded 2D barcoded tubes in 96-well racks with linear barcodes (Brooks Life Sciences) at a normalized, target DNA concentration of 12 ng/µL in 1x TE buffer (10 mM Tris-HCl, 1.0mM EDTA, pH 8.0). Upon arrival, samples/plates were registered in the respective Laboratory Information Management System (LIMS) and stored until use at -20 °C. DNA concentration was confirmed by UV/VIS spectrophotometry (Trinean DropSense system or equivalent). Sequencing libraries were prepared using the NEBNext Ultra™ II PCR-free kit (New England Biolabs). In short, 500 ng of genomic DNA was fragmented to a mean target size of 450-500 bp using high frequency Adaptive Focused Acoustics Technology (AFA) from Covaris Inc (LE220plus instruments and 96-well TPX-AFA plates) . End repair and A-tailing was performed in a single step followed by ligation of unique dual indexed sequencing adaptors (IDT for Illumina) and two rounds of SPRI-bead purification (0.6X) using an automatic 96/8-channel liquid handler (Hamilton Microlab STAR and Tecan Freedom EVO). Quality (concentration and insert size) of sequencing libraries was determined using the LabChip GX (96-samples) instrument (Perkin Elmer). Sequencing libraries were pooled appropriately using automatic 8-channel liquid handlers and sequenced using Illumina´s NovaSeq6000 instruments. Paired-end sequencing on the S4 flowcell (v1.0 chemistry) was performed with a read length of 2x151 cycles of incorporation and imaging, in addition to 2*8 index cycles to a mean coverage of at least 26X per sample. Real-time analysis (RTA) involved conversion of image data to base-calling in real-time. All steps in the workflow were monitored using the in- LIMS with barcode tracking of all samples/plates and reagents.

Supplementary Note 3: Sequence processing pipeline

The deCODE pipeline (Supplementary Fig. 5, Supplementary Fig. 6) for UKB consists of the following steps. An automated pipeline monitors the data coming off the sequencers and starts processing the data when the sequence run folder is ready. The steps taken are:

1. bcl2fastq is run on the sequencer run folder to demultiplex the data and convert each (lane,index) combination into fastq pairs. A checksum is generated for each fastq pair and stored for future reference. The reads in the fastq files are counted and compared against the expected counts coming from the sequencer. The Undetermined read files are inspected, looking for reads that haven't been accounted for.
2. Each pair of fastq files is processed to create a CRAM file. The steps are
    a. Align against GRCh38
    b. Fix mate pair information
    c. Mark duplicates.
    d. Sort in genomic order

    e. calculate checksum and compare with fastq checksum. Failure if they don't match and process is rerun
3. CRAM file is compared with chip genotypes for same sample. Result reported back to the lab. Failure if mismatch rate >2% (potential sample error)
4. QC stats are collected and thresholds applied (Supplementary Fig. 7). Results are reported back to the lab and CRAM is failed if it doesn't pass all quality parameter thresholds. Failed lanes are archived and not used in further processing.
5. A merge process monitors the (lane,index) data and merges the data when it is likely that sufficient data have been collected for a sample. The merge process injects all the necessary header information into the file making it ready for export to UKB.
6. When the file has been created, a checksum is generated for each read group and compared with the corresponding checksums for the fastq files. Failure if the don't match and the merge process is rerun.
7. The merged CRAM file is archived and the upstream data are marked for deletion.
8. Variant calling is performed on the CRAM file and the result is prepared for export to UKB. This includes the production of the BQSR[16] table as well as a gVCF file.
9. QC stats for the merged file are collected and thresholds applied. Results are reported back to the lab.
    a. If the file fails on quantity only, the file is held, the lab initiates a top-up run which is processed as described above and upon completion is merged with the held CRAM file into a new merged CRAM file. That new merged CRAM file is then processed again as described above
    b. If the file fails on other quality parameters, the file is failed and the sample is flagged in the lab. The lab must decide the appropriate action (abandon sample, request a new library)
10. The merged CRAM file, along with variant calling and auxiliary data are sent to UK Biobank

Pipeline details
Alignment
Each read group is aligned to GRCh38 reference (GRCh38 reference with alt contigs plus additional decoy contigs and HLA genes) with bwa mem (v0.7.17)[14] using parameters '-K 100000000 -Y -t 24'. To add MC and MQ tags, samblaster[79] (v0.1.24) is used with parameters '-a --addMateTags'. Duplicates are marked using Picard MarkDuplicates (v2.20.3) with parameters "ASSUME_SORT_ORDER=queryname READ_NAME_REGEX='[a-zA-Z0-9-]+:[0-9]+:[a-zA-Z0-9]+:[0-9]:([0-9]+):([0-9]+):([0-9]+)'", then the results are coordinate sorted using samtools[80] (v1.9).
Merging
Internal thresholds are set for total sequence yield and read count, GC fraction (first and second read in pair) and bias compared to reference, flagging of base conversions in sample preparation, where certain trinucleotides are more commonly observed in sequencing than their reverse complement, flagging of base conversions in sample preparation, where certain trinucleotides are more commonly observed in sequencing than their reverse complement, percentage aligned library read pairs, library insert fragment size distribution, sequencing adapter contamination level, sequence run base call quality values, genotype concordance rate against supplied genome-wide genotype data supplied by UKB for each participant sample, sequence error rate, sequence contamination rate and

genome coverage. Read group bam files are assessed for these parameters and those that pass all the thresholds are merged using samtools[80] merge (v1.9) and converted to CRAM format.

Single sample variant calling

A base quality recalibration table is created using GATK BaseRecalibrator (v4.0.12) with known sites files dbSNP138, Mills and 1000G gold standard indels, and known indels from GATK resource bundle and parameters "--preserve-qscores-less-than 6 -L chr1 .. -L chr22". For each chromosome in chr1 .. chr22, chrX, chrY, the resulting base recalibration table is applied using GATK ApplyBQSR (v4.0.12) with parameters "--preserve-qscores-less-than 6 --static-quantized-quals 10 --static-quantized-quals 20 --static-quantized-quals 30 --create-output-bam-index" and then variants are called using GATK[16] HaplotypeCaller (v4.0.12) with parameters "-ERC GVCF". The resulting 24 chromosome g.vcf files are then combined using Picard[16] MergeVcfs (v2.20.3).

Quality assessment reports

Reports (Supplementary Table 16) to assess the data quality are created using the following programs (in the steps Lane QC, QCPreview and QCStats):

- BamQC (v1.0.0) run on each lane before merge (Supplementary Table 17).
- samtools[80] stats (v1.9) using parameters "-d -p" , i.e. excluding duplicates and overlapping basepairs
- Picard CollectWGSMetrics (v2.20.3) is run with parameters "USE_FAST_ALGORITHM=True MINIMUM_BASE_QUALITY=0 MINIMUM_MAPPING_QUALITY=0 COVERAGE_CAP=1000" once for whole genome, once for autosomes only
- Genotypes are called from .g.vcf files using GATK GenotypeGVCFs (v4.0.12)
- Sample contamination is assessed by running verifyBamId[77] (v1.1.3) with parameters "--ignoreRG --chip-none --free-full --maxDepth 100 --precise" using 1000G phase 3 autosomal SNPs with European MAF > 0.01
- Sample contamination is accessed again using read_haps[78] "-q 30 -mq 30 -c 1 -w 1000"
- Genetic sex is determined using a set of some 100 000 chrX SNPs from gnomad with Non-Finnish European MAF > 0.2. For each variant, the genotype is called using GATK GenotypeGVCFs. Then the ratio of observed to expected heterozygosity assuming diploidy is computed. If ratio > 0.7 the sample is called female, if ratio < 0.3 the sample is called male, otherwise undetermined. Implemented using in-house script gvcf_sexcheck.py
- Picard[16] Genotypeconcordance (v2.20.3) is run with parameter "MIN_GQ=30" to determine concordance with genotypes for quality variants from a chip array.

Supplementary Note 4: Sequence coverage

Our design was to have at least 95% of the genome covered to at least 15x coverage in each sample. Nearly half of the variants detected in this study are singletons, detected in only one sample and a large majority of the variants are rare. GraphTyper requires that at least 4 high quality reads be observed at position for a marker to be called. At 15x coverage the probability that a variant observed in a single individual would be misclassified due to random sampling is 3.5%. Sequence coverage across the genome computed over 1,000 randomly selected samples can be seen in Supplementary Fig. 8.

Supplementary Note 5: SNP and indel calling with Calling with GATK

We used GATK versions 4.1.7.0 for all regions. Regions that failed were rerun with version 4.1.8.1.

The process starts by slicing the 50kb region (padded with 1kb) of every sample file with tabix (from htslib[80] version 1.9) onto local disk and then builds a GenomicsDB with GATK GenomicsDBImport. The command we ran was the following:

```
gatk --java-options "-Xmx${JAVAMEM_TOTAL}G
 -Xms${JAVAMEM_TOTAL}G
 -DGATK_STACKTRACE_ON_USER_EXCEPTION=true"
    GenomicsDBImport
    --genomicsdb-workspace-path ${GDB}
    --intervals ${REGION_PADDED}
    --tmp-dir ${GDB_TMP}
    --sample-name-map ${SNMAP}
    --batch-size ${BATCH_SIZE}
    --reader-threads ${RTHREADS}
```

where `SNMAP` is the tab-delimited text file of sample names and paths to samples. The parameters `--batch-size` and `--reader-threads` are used to reduce memory usage.
We then split the padded region into as many smaller regions as the number of threads, and pad those regions again with 1kb. The GenotypeGVCFs command was then ran wrapped in GNU parallel

```
parallel --halt=now,fail=1
 --jobs=${NTHREADS}
 --xapply
    "${GATK_WITH_OPTS} GenotypeGVCFs
    --genomicsdb-use-vcf-codec
      -R ${REF}
      -V gendb://${GDB}
      --tmp-dir=${tmpdir}
      -L {1}
      -O {2} &&
    ${GATK_WITH_OPTS} SelectVariants -R ${REF}
    -V {2}
    -L {3}
    -O {4}"
    ::::: ${REGIONS_PADDED} ${SPLITFILES_PADDED} ${REGIONS} ${SPLITFILES}
```

where `REF` is the reference, `REGIONS_PADDED` is a file containing the padded subregions, `SPLITFILES_PADDED` is a file containing the intermediate padded output file paths, `REGIONS` is a file containing the subregions and `SPLITFILES` is a file containing the intermediate output file paths after selecting the variants.
We then run the following command to combine the intermediate output files

```
gatk --java-options "-Djava.io.tmpdir=$tmpdir
 -Xmx${JAVAMEM_TOTAL}G
 -Xms${JAVAMEM_TOTAL}G
 GatherVcfs -R ${REF}
  -O ${OUT}
  --arguments_file ${VARARGS}
```

where VARARGS is a file containing arguments for all input intermediate vcfs.

It should be noted that running GATK out of the box will cause every job to read the entire gVCF index file (.tbi) for each of the 150,119 samples. The average size of the index files is

4.15MB, so each job would have to read 4.15*150,126 = 623GB of data on top of the actual gVCF slice data. For 60,000 jobs, this would amount to 623GB*60,000 = 37PB or 25.2GB/sec of additional read overhead if the jobs are run on 20,000 cores in 17 days. This read overhead will definitely prevent 20,000 cores from being used simultaneously. However, this problem was avoided by pre-processing the .tbi files and modifying the software reading the gVCF files from the central storage in a similar fashion as we did for GraphTyper and the CRAM index files (.crai).

All jobs were run initially with 6 cores and 100GB of RAM. Jobs that failed due to memory were rerun with more memory, up to a maximum of 1,458GB. Calling for 320 of the 50kb regions failed using GATK version 4.1.7.0, either due to 1,458GB of memory being insuffient or program failure. These regions were split into 3,066 5kb regions (regions at the end of chromosomes were smaller than 50kb) and rerun with GATK version 4.1.8.1. 320 regions, representing 1.6Mb, of the 3,066 regions again failed calling with GATK version 4.1.8.1. No further attempt was made to call these regions. Total reserved CPU time on cluster was 9.6M CPU hours and total effective compute time 4.0M CPU hours. The difference in these numbers is explained by the fact that while 6 cores reserved for the program it may not utilize all at the same time.

Supplementary Note 6: Evaluation of SNP and indel callers across 500 random regions

Prior to running variant calling on the whole dataset, we evaluated joint variant callers for the UKB sequencing effort. We evaluated the quality of the genotype calls and feasibility of variant calling 150,000 or more WGS samples. There were some minor differences between this call set and the final set, for example we included seven Genome in a Bottle (GIAB) samples for evaluation purposes in the evaluation set. However, we believe these differences should have minimal effects on the results.

Input data

The evaluation was run on the set of 150,126 WGS samples including 7 WGS samples obtained from the GIAB Consortium (websites).

All of the GIAB BAM files were down sampled to approximately 30x coverage using `samtools view -s 42.FRAC` option with seed 42 and FRAC was the fraction of reads to keep such that 30x was obtained to represent more closely the target coverage of the other input files. Samtools version 1.9 was used.

We evaluated 500 regions (50kb each). We selected the regions at random by listing all such regions (only excluding regions which contained only Ns) and using the first 500 regions from the output of `sort -R`.

SNP and indel calling with GraphTyper

We ran GraphTyper as described for the whole dataset, with the additional option `--normal_and_no_variant_overlapping`. This was done to simplify the comparison to the GIAB truth sets using the files which contained no variant overlaps as `rtg vcfeval` sometimes misinterprets overlapping variants. This option however should normally be omitted to generate only a set where variants may overlap. We used the non-overlapping

set when comparing to the GIAB truth sets but in all other analysis of GraphTyper variants we used the "normal" variants set.

Resource Requirements

GraphTyper

The GraphTyper jobs were run on 12 cores and 60GB of memory reserved for each job (5GB/core). Average CPU time was 82 hours and average elapsed walltime was 7.8 hours, resulting in average reserved core time (walltime*12) of 93.6 hours. For 150k samples and the entire genome (60,000 50kb slices), this translates to overall compute time of 93.6*60,000 = 5.62M hours, or 12 days if the jobs are run in parallel on 20,000 cores. The input data to GraphTyper are CRAM files. The average size of an input CRAM file is 17.8GB, so the total size of data to be read is 17.8GB*150,126 = 2.7PB. Reading those data once over a period of 12 days was estimated to result in average sustained read rate of 2.6GB/sec, assuming no overhead.

GATK HaplotypeCaller

The GATK jobs were run on 6 cores and 80GB of memory reserved for each job (13.33GB/core). With these settings, 488 of the 500 jobs completed. The 12 remaining jobs finished when given more memory. The average cpu time was 53.4 hours and average elapsed walltime was 22.5 hours, resulting in average reserved core time (walltime*6) of 135.0 hours. For 150k samples and the entire genome (60,000 50kb slices), this translates to overall compute time of 135*60,000 = 8.1M hours, or 17 days if the jobs are run in parallel on 20,000 cores.

Output sizes

Both programs return a gzip compressed vcf file (.vcf.gz), one for each region. The average file size for GATK is 12.0GB while for GraphTyper it is 7.6GB. For 150k samples and the entire genome, this translates to a total estimated output size of 12GB*60,000 = 720TB for GATK, while the output for GraphTyper was 7.6GB*60,000 = 445TB. This difference in size may in part be explained by the fact that GATK reports more variants and in part by the fact that GATK does not cap genotype likelihoods at 255 like GraphTyper, thus resulting in worse compression ratio.

Comparison to the GIAB truth sets

In both sets we genotyped seven GIAB samples. We extracted the calls made in each of those sample in the 150k sample run and compared to their v3.3.2 truth set in high confidence regions. Variant callers do not generally have the same output when genotyping a single sample compared to extracting the sample from a multi-sample run.
We ran the tool RTG-vcfeval[81] to make the comparison to the truth set in the high confidence regions which overlapped the 500 regions. For all of the samples, GraphTyper had both higher sensitivity and precision than GATK on the full sets (Supplementary Table 1). The difference between the two callers was small (99.44% vs. 99.34%,Supplementary Table 1) for SNPs but more marked for indels (97.58% vs. 94.14%, Supplementary Table 1), were both methods performed much worse on indels only compared to single sample calling, indicating that indel calling is particularly difficult when genotyping a large population.

Overview of genotyping results

We analyzed the evaluation set to further learn the differences between the two genotyping datasets. In this analysis, all of the variants from the VCF were analyzed on per alternative allele basis. Therefore the number of variants we report here is higher than the number of VCF records due to multi-allelic variants.

Variant counts
We counted the number of variants in each dataset (Supplementary Table 18,Supplementary Fig. 9). We saw that there were more variants in the GATK dataset. However, GATK also had greater number of missing calls (genotype quality = 0 in the VCF). It is expected that the ratio of SNP transitions to transversion is roughly 2.1-2.3 in humans genome-wide. We saw lower ratios in the call sets, but it was higher in the GraphTyper set (1.639) than in the GATK set (1.507).
Indel sizes were limited to 100 bp in the GraphTyper dataset but had a larger range in the GATK set (Supplementary Fig. 10).

Batch Effect by Sequence Center
Further, we investigated how many common variants had genotype calls which were highly correlated to the sequence center for which the sample was sequenced in. As the batches had a highly different amount of samples we randomly selected 10,000 samples from each batch and restricted our analysis to those sample. We tested whether there were more alternative calls (either ref/alt or alt/alt calls) compared to the number of reference calls in each set using Fisher's exact test. Only common variants were tested, as we expect fewer rare markers to be rejected due to smaller sample size. We used a p-value threshold of 10^{-6}, any variants with a lower p-value in any of three tests were considered as failed.

To our surprise, we saw that a large fraction of the common variants are highly correlated with the sequence center (Supplementary Table 19), on average of 7.47% and 1.01% of variants for GATK and GraphTyper, respectively.

Singletons variants
Supplementary Fig. 11a) shows the distribution of singletons by mutation classes between and the variant allele frequency (VAF) of singletons. A VAF of 50% is expected for singletons.

Parent-Offspring Trio Analysis
There were 28 parent-offspring trios in the dataset. We analyzed Mendelian errors in the trios as well as the rate of transmission of alternative alleles from parent to offspring. We assume that the alleles transmit from parent to child with equal likelihood and use the transmission rate to estimate false discovery rate and number of germline variants in the datasets. More info on the method is described[15].

Mendelian Errors
We measured non-reference Mendelian errors by checking for Mendelian consistency when a parent had an alternative genotype (ref/alt or alt/alt) (Supplementary Table 3).

Estimating FDR and number of TP in trios

Using transmission rate in trios we estimate both false discovery rate (FDR) and the number of true positive (TP) variants[15]. We also stratified the results by variant type. We estimated that GraphTyper finds slighlty more true positive variants across all variant types with a much lower false discovery rate than GATK (Supplementary Table 3). GATK finds more true positive SNPs, but GraphTyper more true positive indels.

Monozygotic Twin Non-Ref Error Rate
There were 14 pairs of monozygotic twins in the dataset. We checked how many of the non-reference variants were consistent between a pair of monozygotic twins. We considered a variant to be non-ref if either twin had an alternative allele in their genotyped. GraphTyper had lower error rate between monozygotic twins (Supplementary Table 3C).

Summary
Overall, we find that GraphTyper performs consistently slightly better than GATK in the variant quality experiments. Despite that GATK reports more variants than GraphTyper, we estimate that GraphTyper's sensitivity is better in both the GIAB truth set comparison and family trio analysis. There appears to be larger gap between the methods in terms of noise, GATK performs worse in precision in the GIAB comparison, in the family trios we estimated that GATK's false discovery rate is twice as much as GraphTyper's, and 7-fold more common GATK variants failed the batch effect test compared to GraphTyper.

Supplementary Note 7: Comparison of final GraphTyper and GATK call sets.

In addition to the two callsets, we also define the set "GraphTyperHQ" as the set of GraphTyper alternative alleles with AAScore above 0.5.

Variant counts and frequency classes
We counted total number of variants in the sets (Supplementary Table 7). When counting the number of "variants" in any context hereafter, we are referring to alternative alleles excluding the alleles that are denoted as '*' in the VCF.

An informative call is one with non-zero quality (GQ > 0). We saw that GATK had more variants but also much more missing calls. We split the sets into three frequency classes: Common (Allele frequency (AF) > 0.1%), rare (AF <0.1%, excluding singletons) and singletons (one called carrier in the set). A vast majority of the datasets (95.6% - 96.0%) are have an allele frequency below 0.1%. Singletons account for nearly half of the variants (43.9-45.5%) (Supplementary Table 7).
The transition transversion ratio was 1.550, 1.642 and 1.657 for the GATK, GraphTyper and GraphTyperHQ datasets, respectively (Supplementary Table 7B, Supplementary Fig. 12).

Batch effect by sequence center
We investigated how many common variants had genotype calls which were highly correlated to the sequence center, i.e. the location which the sample was sequenced at. We randomly selected 10,000 samples from each sequencing center analysis pipeline and restricted our analysis to those samples. We tested whether there were more alternative calls (either ref/alt or alt/alt calls) compared to the number of reference calls in each set using Fisher's exact test. Only common variants were tested, as we expect rare variants are less likely to be rejected due to limited sample size. The same variant often fails multiple

tests, 5.69%, 0.97% and 0.20% of common variants associate with sequencing center for the GATK, GraphTyper and GraphTyperHQ datasets, respectively (Supplementary Table 20).

Variant transmission in parent-offspring trios and monozygotic twin pairs
There were 28 parent-offspring trios in the dataset. We analyzed the rate of transmission of alternative alleles from parent to offspring. We assume that the alleles transmit from parent to child with equal likelihood and use the transmission rate to estimate false discovery rate (FDR) and number of germline true positive (TP) variants in the datasets[15]. From the family trios we estimate that GraphTyper has more true positive variants while also having lower rate of false positive ones. GraphTyperHQ has considerably lower false discovery rate than the GATK call set (Supplementary Table 2).
There were 14 pairs of monozygotic twins in the dataset. We checked how many inconsistent genotypes in the twins were on average in a 1MB region (ICPM). We also calculate the total non-reference consistency rate among, by checking for consistency among all calls where either twin had a call with an alternative allele. The raw GATK and GraphTyper datasets have many inconsistent calls between monozygotic twins but the filtered GraphTyper dataset is much more consistent (Supplementary Table 2).

Supplementary Note 8: Batch effects in final dataset

Sequencing was performed in three batches; individuals sequenced at deCODE genetics (deCODE), sequenced at the Welcome Trust Sanger Institute processed using Vanguard phase pipeline (Sanger Vanguard), sequenced at the Welcome Trust Sanger Instititute using the main phase pipeline (Sanger Main). From the lists of individuals, we constructed six different phenotypes, comparing each sequencing batch both to the two other sequencing batches both jointly and separately. Association tests were performed per cohort and both for the raw genotypes and the imputed dataset, following the protocol describe in subsection “Association testing”. Association results are presented for both a filtered and an unfiltered dataset. For the raw genotypes the filtered set refers to markers with AAscore > 0.5, or the GraphTyper HQ set. For the imputed genotypes the filtered set refers to markers markers with AAscore > 0.5 and Imp info > 0.8.

Batch effects for sequencing center are shown in Supplementary Table 22 for raw genotypes and in Supplementary Table 23 for imputed genotypes, with results conditioned on frequency and association p-value. Considerable batch effects can be observed in all datasets. As expected, lower levels of batch effects were detected for the filtered dataset. More common variants show higher levels of batch effects. We note that marker batch effect is conflated with missing data in genotype calling.

For the purpose of the Supplementary Table 22 and Supplementary Table 23 frequency is computed from genotype likelihoods, where the likelihoods are transformed into probabilities that the individual is a carrier. In this way an individuals with no sequence reads is assigned frequency 50%, upweighing rare markers where a large fraction of markers have missing data. Alternatively frequencies can be computed from the carrier status of individuals without missing data.

Supplementary Note 9: Overlap with UKBB WES SNPs

Comparison based on minor allele frequency

A recent UKB WES dataset has 200,000 individuals (WES200k[59]). In the dataset there are 1,047,397 SNPs with WES AF >0.01% and 353,889 with WES AF >0.1%. We checked how many of those were not found in the WGS datasets. 1.81, 0.44 and 1.60% of variants with frequency > 0.01% in the WES200k dataset were missing the in the GATK, GraphTyper and GraphTyperHQ datasets, respectively (Supplementary Table 5).

Variant normalization

To reliably compare two datasets (the result of different samples, technologies or tools), the data needs to be in a standardized format. The commonly used VCF format is unfortunately very ambiguous:

1. Two variation events may be represented as a single multi-allelic VCF record in one set or as two VCF records in another.
2. A single variation event has many equivalent representations, i.e. variants are not required to be left-aligned and parsimonious[82].
3. While records are required to be ordered by POS, two records with the same POS have no defined order. This makes line-wise comparisons and merges difficult. In particular, the order generated by bcftools norm is not alphabetical.
4. Different conventions exist for how to name chromosomes ("Chr1" vs "1"; "ChrX" vs "Chr23" vs "23").
5. IDs are absent from some files, making it more difficult to return to the original entry after changes have happened.

Our normalization pipeline employs bcftools norm to split multi-allelic variants and to left-align and trim them. It enforces a naming convention for the chromosomes ("Chr1" ... "ChrX") and adds an ID-String if missing. Finally, the data is split into 50KB regions and sorted by "Chrom,Pos,Ref,Alt". Since normalization may influence the POS field of a VCF record, it may fall into a different 50KB bin than before; these cases are handled.
Once all datasets are normalized, a merged dataset is created from them. This consists of one set of VCF files where all INFO fields from the original datasets are included with a set-specific prefix, e.g. "GATK_AF" instead of "AF". The original datasets' ID, QUAL and FILTER fields are also included in the merged files' INFO fields as "GATK_ID", "GATK_QUAL" etc. This representation of the data is sparse because missing entries do not take up space. For analysis purposes, a TSV or GOR[Z] file can be created for individual regions or full chromosomes. The transformation from .VCF.GZ files to .GORZ and further operations (e.g. JOINs) are efficiently possible, because our VCF records are already fully sorted.

Comparison of WES and WGS call sets on the same sets of samples

In an attempt to make a judicial comparison between WES and WGS as well as between the GraphTyperHQ and GATK call sets we analyzed seperately the calls made for a subset of 109,618 individuals included in our dataset as well as the 200k release of WES data from the UKB[59].
Variants not present in any of the 109,618 indivdiuals were removed from analysis, resulting in 558,128,486 GraphTyperHQ variants and 13,815,704 WES variants. We then split the variants by functional annotation and tabulated the number of variants shared between the two call sets and the number of variants absent from the other call set (

Table 3).
To further explore the accuracy of genotype callers we analyzed specifically variants inside regions that are purportedly captured by exome sequencing (websites,Supplementary Table 21), 6,608,669 variants are found in all three call sets. Variants in one call set and not another may be either true or false positives. A priori, we would expect that variants found in two call sets to be a strong indication of the variant being a true positive. This analysis is complicated by the fact that although we have filtered the set of GraphTyper variants GATK variants have not been filtered for true positives.
A total of 87,773 variants are found by both GATK and WES but missed by GraphTyperHQ. 32,875 of these variants were present in the unfilterd GraphTyper dataset but filtered due to low AAscore. 56,909 out of the 87,773 variants have the same primary carrier in both datasets, while the remaining 30,864 are found by both callers but not in the same sample. These variants represent a shared tendancy of false positive calls at the same variant (but in different samples) across both datasets. Best practices use of GATK recommends filtering of variants based on a number of factors. While we have not computed all of these, we computed for these variants what we believe are some of the most common causes of failure; failing variants that have variant allele frequency (VAF) below 25%, failing variants that are not supported by reads from both strands and failing variant that are not supported by both a read that is first in pair and one that is second in pair. Applying these three filters removed 69.3% of the 56,909 variants, suggesting at most a small fraction of the variants found by both GATK and WES, but not GraphTyper, are in fact called reliably enough to be used in a recommended genetic analysis.
Cursory analysis of the variants found by both GraphTyper and WES, but not GATK suggested that these were similarly possibly problematic.
Analysis of variants found by both GATK and GraphTyper however suggested that these were in large part true positives. We considered the distribution of the 898,764 singletons shared between the callers and found their distribution (XAF 78,229 (8.70%), XBI 564,346 (62.79%), XSA 71,823 (8.00%), OTH 184,366 (20.51%)), to be similar to that of the distribution of singleton calls overall (XAF 746,289 (8.40%), XBI 5,731,044 (64.50%), XSA 707,379 (7.96%), OTH 1,701,318 (19.15%)). We would expect false positive calls due to sequencing artifacts would be similar to the fraction of individuals from each cohort in our intersected sequencing set (XAF 2.05%, XBI 87.89%, XSA 2.08%, OTH 7.99%).

Supplementary Note 10: Microsatellite calling with popSTR

We followed the protocol described above for Graphtyper before we ran PopSTR(v2.0) and created chopped CRAI indices for all samples as well as a reference sequence cache for each processed region.
We scanned all CRAM files in 50kb regions using the popSTR subcommand computeReadAttributes.
The format of the command was:
```
popSTR computeReadAttributes ${CRAI_TMP}/sampleList.txt ${RESULT_TMP}
markerList flanking <(readLength-2*flanking) "." longRepeats N
```
Results over a predetermined set of microsatellites from chr21(our kernel) were used to estimate a slippage rate for each individual using the popSTR subcommand computePnSlippageDefault.
The format of the command was:

```
popSTR computePnSlippageDefault
-PL $sample
-AD ${RESULT_TMP}/attributes/chr21/
-OF ${outDir}/pnSlippage
-FP $sampleIDx
-MS ${codeDir}/kernelSlippageRates
-MD ${codeDir}/kernel/kernelModels
```

Combining CRAM analysis results and sample slippage rates we performed genomewide genotyping using the popSTR subcommand msGenotyperDefault

The format of the command was:

```
popSTR msGenotyperDefault -ADCN ${RESULT_TMP}/attributes/${chrom}/ -PNS
pnSlippage -MS ${RESULT_TMP}/markerSlipps/${chrom}/markerSlippage -VD
${RESULT_TMP} -VN vcfName -ML markerList -I $idx -FP 1
```

`CRAI_TMP` is a path to the chopped CRAI files on the local disk, `RESULT_TMP` is a folder on the local disk to store results, `flanking` is a parameter specifying the number of bps required to anchor a read to the microsatellite, `readLength` is the length of reads in the CRAM file, `markerList` is a list of all microsatellites in the 50kb region being analysed, `outDir` is a directory to store sample slippage results, `sampleIDx` is the index of the sample being analysed in the `sampleList.txt`, `codeDir` is the directory where popSTR and its dependencies are stored and `$idx` is the index of the region being analyzed.

Filtering of microsatellites

We recommend the following best practice filtering guidelines.

Filter marker where:

average coverage < 10 or average coverage > 75

command:
```
bcftools query -f
'%CHROM\t%POS\t%INFO/nReads\t%INFO/nPnsWithReads\n' $file |
awk '{print $1,$2,$3/$4}' | awk '{if ($3>10 && $3<75){print
$1\t$2}}' > pass; bcftools view -T pass -o filtered_${file} -O
z $file; tabix filtered_${file}
```

average genotype quality < 20

command:
```
bcftools query -f
'%CHROM\t%POS[\t%GT\t%GQ]\n' $file | awk '{sum=0; miss=0;
avail=0; for (i=4;i<=NF;i+=2){if ($(i-
1)=="./."){miss+=1}else{sum+=$i; avail+=1}}
if(avail>0){mean=sum/avail}else{mean=0} print $1,$2,mean}' |
awk '{if ($3>20){print $1\t$2}}' > pass; bcftools view -T pass
-o filtered_${file} -O z $file; tabix filtered_${file}
```

number of individuals with reads < 75,000

command:
```
bcftools query -f
'%CHROM\t%POS\t%INFO/nPnsWithReads\n' $file |awk '{if
($3>75000){print $1\t$2}}' > pass; bcftools view -T pass -o
filtered_${file} -O z $file; tabix filtered_${file}
```

number of reads not supporting genotype/number of reads available > 0.3

command:
```
bcftools query -f
'%CHROM\t%POS\t%INFO/nNonSupportReads\t%INFO/nReads\n' $file |
awk '{if ($3/$4<0.3){print $1\t$2}}' > pass; bcftools view -T
pass -o filtered_${file} -O z $file; tabix filtered_${file}
```

A total of 2,393,292 variants pass these filters.

Supplementary Note 11: Imputation results

We refer to a variant as being reliably imputed if its L1oR2 score is greater than 0.5 and imputation info[1] was above 0.8.

Imputation and phasing accuracy of SNPs and indels for the GraphTyperHQ set is shown in (Fig. 3, Supplementary Fig. 4, Supplementary Table 11). GraphTyperHQ filters variants based on an AAscore of 0.5. Requiring higher AAscore allows a higher fraction of variants to be imputed (Supplementary Fig. 13). We found that variants located > 100kb from a chip genotyped variant and variants in regions that were placed on different chromosomes on GRCh38[13] and CHM13[83] imputed less accurately than others.

SVs and microsatellites are imputed less accurately than SNPs and indels (Supplementary Fig. 4), in part due to difficulty in genotyping those variants. For microsatellites, this may in part be attributed to the high mutation rate of microsatellites and in part to the fact that the results are presented for the unfiltered microsatellite set, we expect that a higher fraction of microsatellites would impute after filtering.

Comparison of imputation from GATK and GraphTyper variants

We imputed all variants genotyped by GATK and GraphTyper across chr22, 10-11Mb. We define a variant to be imputed if the phasing leave-one-out r2[1] (L1or2) was at least 0.5 and imputation info[1] was at least 0.5. We present the number of variants that could be imputed as a function of frequency and variant type (Supplementary Table 4). Although more variants are called by GATK, there are more variants called by GraphTyper that can be imputed, across all frequency classes and variant types.

Supplementary Note 12: Genome annotation

We downloaded Refseq and Ensembl gene map annotations from Ensembl[84], version 100 database. The gene maps were transformed to segments with each position in GRCh38 annotated as at least one of 3'utr, 5'utr, coding, downstream, intergenic, intronic, spliceregion, splicesite, upstream.

These regions were grouped and ordered by precedence:

1 – coding – coding
2 – splice – spliceregion, splicesite
3 – 5‘UTR – 5‘UTR
4 – 3‘UTR – 3‘UTR
5 – proximal – upstream, downstream, intronic
6 – intergenic – intergenic

Each position was then given annotation according to its lowest precedence rank annotation, e.g. a position annotated as both spliceregion and 5‘UTR was given the annotation „splice“.

Supplementary Note 13: WGS individuals carrying actionable genotypes meeting ACMG criteria

The American College of Medical Genetics and Genomics (ACMG) recommends reporting secondary findings in a list of actionable genes associated with diseases that are highly penetrant and for which a well-established intervention is available[18]. The initial version (ACMG SF v1.0) was published in 2013 and included 56 actionable genes but has since been updated twice to ACMG SF v2.0 and v3.0 listing 59 and 73 actionable genes, respectively. 2.0% of the 49,960 WES individuals from the UKB were reported[19] to carry an actionable variant in at least one gene from the ACMG v2.0 list of 59 genes. Using their criteria, we detected actionable genotypes in 2.6% of 150.119 WGS individuals. When applying the same criteria to the ACMG v3.0 gene list (73 genes), the fraction of individuals carrying an actionable genotype increases to 3.5%. In the ACMG v3.0 list of actionable genes, HFE p.Cys282Tyr homozygotes are recommended to be reported, but does not fullfill the previously described criteria[19]. In the set of 150,119 WGS individuals, we observe 929 HFE p.Cys282Tyr homozygotes (0.62%), thereby increasing the fraction of individuals carrying an actionable genotype in one of the ACMG v3.0 genes to 4.1%.

Supplementary Note 14: Genotype count of rare LoF variants

We counted the number of autosomal heterozygous and homozygous genotypes per individual for rare LoF variants (minor allele frequency (MAF)<1% in all 3 groups, XBI, XAF and XSA). LoF variants are those annotated by the Variant Effect predictor as having consequence as one of: stop gained, frameshift, splice acceptor, splice donor og start loss. Heterozygous counts were based on WGS data, and homozygous counts were based on phased genotypes.

Supplementary Note 15: GWAS enrichment analysis

We have previously described a likelihood-based inference model for estimating the enrichment of trait-associating sequence variants on the basis of their annotations[32]. Similar to our earlier work[32] we defined a set of 22.8M high-quality sequence variants identified as mono-allelic SNPs or Indels in a set 28,075 whole genome sequenced individuals from the Icelandic population.

The high-quality SNP-indels (22.8M) were then tested for association to a selected set of 614 human diseases and other traits. For each trait, we split the genome into 10Mb windows and selected the strongest sequence variant association from each window where $p < 1 \cdot 10^{-9}$. Then, for each chromosome, we sorted the selected sequence variants according to P-value to then determine whether the second best variant still associates at $p < 1 \cdot 10^{-9}$ after adjusting the trait for the strongest variant on that same chromosome. If so, this second best sequence variant was incorporated into a final set of „independently associated“ variants for that trait, and the process continued for all other sequence variants down the list –each time adjusting for „stronger“ variants on the same chromosome. This yielded a set of 3,431 independently associated sequence variants in 322 traits. For each of the 3,431 trait-associated variants, we searched for correlated sequence variants (r2>0.80) in the same Icelandic population. In this way, a given trait association variant along with its correlated variants (found in linkage disequilibrium; LD) defines an association

signal. P-values were estimated by determining how often the enrichment estimate (E) is above or below E=1 by bootstrapping (N=5000) of the GWAS association signals.
We then annotated sequence variants according to whether or not they are found within regions that show low and high DR scores (1st percentile versus 99th percentile; i.e. most and least conserved regions, respectively); refered to as DR-1% and DR-99%, respectively. In this model, we specified eleven other annotations of sequence variants: loss of function, missense, splice-donor/acceptor, splice region, synonymous, 5kb gene-upstream, 5kb gene-downstream, 3´UTR, 5´UTR, intronic and the remaining sequence variants as „other“ (not found in any of the specified annotation categories). Similarly, we specified another model wherein we estimated enrichment for DR-5% and DR-95%.

Supplementary Note 16: Overlap with ENCODE regions

We used annotations from ENCODE[9] and compute the odds ratios these annotations in regions of different DR scores. We label each bp in the genome with a_{11},a_{12},a_{21} or a_{22}, where the first number represent that the bp was annoted with the given ENCODE annotation (1) or not (2) and the second number represents that the DR score was above (1) or below (2) a given threshold.
The odds ratio for the ENCODE annotation given the DR score threshold is then:
$OR=a_{11}/a_{21} \times a_{22}/a_{12}$.
The marker label parameters are computed for each one of the annotations on a set of 1Mb windows across the regions annoted with a DR score. The mean odds ratio is computed by summing up the individual parameters for the complete set of windows. We use bootstrapping to estimate the confidence limits for the odds ratio we, for each bootstrap sample we sample with replacement from the complete set of 1Mb windows, sum up individually the resulting set a_{ij}‘s and compute the odds ratio for the bootstrap sample. The odds ratio is computed for a total of 1000 bootstrap samples and the confidence intervals defined between the 2.5% and 97.5% quantile of the resulting dataset.

Supplementary Note 17: RNA sequence data

RNA sequencing was performed on samples from cardiac right atrium of 169 Icelanders. The data and subsequent sequence alignment to GRCh38 has been described[85]. To estimate the effect of deletion of exon 6 in transcript ENST00000168977.6 of *NMRK2* we counted fragments aligning from the donor site of exon 5 to either acceptor site of exon 6 or exon 7 (Extended Data Fig. 9).

Supplementary Note 18: Computing principal components within cohorts

Microarray data
For all cohorts, we first removed variants with missingness >3% and 135 individuals with genomewide missingness >5%. We then removed a canonical set of long-range high-LD regions and all indels.

For the XAF and XSA cohorts, the following procedure was followed. We first excluded both individuals from each pair of relatives with kinship coefficient 0.0625 or greater; these

excluded individuals were later projected onto the principal components. We then pruned for variants in complete linkage disequilibrium (r2 = 1) using plink --indep-pairwise 200 25 0.999999, and then removed all variants with MAF <1%. PCA for these two cohorts was performed using smartpca[86] with parameters numoutevec: 45, numoutlieriter: 0, ldregress: 200, and ldposlimit: 100000. We then projected all relatives using the OADP method implemented in bigsnpr's[87] function bed_projectSelfPCA().

A slightly different approach was used for the XBI set, due to the very large number of individuals. We first excluded: individuals from each pair of relatives at a kinship coefficient threshold of 0.0442 or greater; individuals with inbreeding of 0.1 or greater; individuals with genomewide missingness 1% or greater; and all remaining individuals defined as "HetMiss" (heterozygosity/missingness) outliers by UKB. We next removed variants with < 0.05% MAF and a Hardy-Weinberg disequilibrium p-value (calculated with plink --hwe midp) of <1e-100. Then LD clumping was performed using bigsnpr's bed_clumping() function using thr.r2 = 0.2 and [window] size = 500 [kb]. We calculated 30 PCs on the remaining variants and individuals using bigsnpr's bed_randomSVD(), and the previously excluded individuals were projected onto these PCs using OADP.

WGS data
To prepare each WGS cohort for PCA, we first removed all variants with missingness >3%. We then excluded individuals with genomic inbreeding over 0.1 and both individuals in any pair of 3rd degree or closer relatives. The excluded individuals were later projected onto the principal components. After excluding these individuals, we removed all singleton variants. For XBI in particular, we also removed all variants with minor allele count <10, in order to make computation more tractable and to minimise the influence of very recent genealogical structure.

bigsnpr[86] was used to remove a canonical list of long-range, high-LD regions [long-range LD ref] and then perform LD clumping using bed_clumping() with an r2 threshold of 0.1 and a window size of 5 megabases. We then used bed_randomSVD() in bigsnpr to calculate 50 PCs on each of the cohorts.

The first six principal components in each cohort are shown in Supplementary Fig. 20, Supplementary Fig. 21 and Supplementary Fig. 22.

Supplementary Note 19: Inbreeding

Genomic inbreeding in the form of F_{ROH} (proportion of the genome in runs of homozygosity) was calculated on microarray genotypes using PLINK[88] v1.9 and the same parameters specified in ROHgen2[89]: homozyg-window-snp 50; homozyg-snp 50; homozyg-kb 1500; homozyg-gap 1000; homozyg-density 50; homozyg-window-missing 5; homozyg-window-het 1. Genotype data had been filtered to remove variants: that were not in the "in_HetMiss" set defined by UKB; that had >2% cohortwide missingness; or that were found to have highly discordant allele frequencies compared to other British–Irish datasets or to be in apparent inter-chromosomal LD[90].

Supplementary Note 20: IBD segment computation

We called IBD segments between UKB individuals' microarray genotypes using KING v2.2.4 --ibdseg[91]. Genotype data was split into 90 batches and run using --projection mode to calculate IBD between batches. Kinship coefficients quoted throughout the supplementary refer to the PropIBD values reported by KING divided by 2. Genotype data had been filtered to remove variants with cohortwide missingness >3%.

Supplementary Note 21: ADMIXTURE

We assigned proportions of continental-scale ancestry to all UKB microarray genotypes using ADMIXTURE[76]. ADMIXTURE was run on --supervised mode using the 1000G populations CEU (Northern Europeans from Utah), CHB (Han Chinese in Beijing), ITU (Indian Telugu in the UK), PEL (Peruvians in Lima), and YRI (Yoruba in Ibadan, Nigeria) as training data. The 1000G training data had previously been filtered to remove close (at least 2nd degree) relatives using KING[91] --kinship, to remove some apparent genomic ancestry outliers using PCA and leave-one-out unsupervised ADMIXTURE (especially PEL individuals with high European ancestry), and also pruned for LD using PLINK[88] v1.9 --indep-pairwise 50 5 0.2. The ADMIXTURE program was run for batches of 30 UKB individuals at a time and the results subsequently merged.

Supplementary Note 22: Birthplace data

All location analyses were performed in R using the sf package[92], the sp package[93], and the gstat package[94]. Spatial interpolation of birthplaces was performed using linear variogram models (gstat::vgm(), range 60,000) and ordinary kriging (gstat::krige(), nmax = 300).

For some analysis, we binned the birthplaces into the following administrative divisions: the ceremonial counties of England; the historic counties of Wales; the 1975 local government areas of Scotland; the Isle of Man, Northern Ireland, and the [Republic of] Ireland each as their own divisions; and Jersey and Guernsey grouped together into a division we labelled the Channel Islands.

Supplementary Note 23: Websites:

GraphTyper
https://github.com/DecodeGenetics/graphtyper

GATK resource bundle
gs://genomics-public-data/resources/broad/hg38/v0

Svimmer
https://github.com/DecodeGenetics/svimmer

popSTR
https://github.com/DecodeGenetics/popSTR

Dipcall

https://github.com/lh3/dipcall

RTG Tools
https://github.com/RealTimeGenomics/rtg-tools

bcl2fastq
https://support.illumina.com/sequencing/sequencing_software/bcl2fastq-conversion-software.html

Samtools
http://www.htslib.org/

samblaster
https://github.com/GregoryFaust/samblaster

BamQC
https://github.com/DecodeGenetics/BamQC

GIAB WGS samples
- *HG001 https://ftp-trace.ncbi.nlm.nih.gov/ReferenceSamples/giab/data/NA12878/NIST_NA12878_HG001_HiSeq_300x/NHGRI_Illumina300X_novoalign_bams/HG001.GRCh38_full_plus_hs38d1_analysis_set_minus_alts.300x.bam*
- *HG002 https://ftp-trace.ncbi.nlm.nih.gov/ReferenceSamples/giab/data/AshkenazimTrio/HG002_NA24385_son/NIST_HiSeq_HG002_Homogeneity-10953946/NHGRI_Illumina300X_AJtrio_novoalign_bams/HG002.GRCh38.60x.1.bam*
- *HG003 https://ftp-trace.ncbi.nlm.nih.gov/ReferenceSamples/giab/data/AshkenazimTrio/HG003_NA24149_father/NIST_HiSeq_HG003_Homogeneity-12389378/NHGRI_Illumina300X_AJtrio_novoalign_bams/HG003.GRCh38.60x.1.bam*
- *HG004 https://ftp-trace.ncbi.nlm.nih.gov/ReferenceSamples/giab/data/AshkenazimTrio/HG004_NA24143_mother/NIST_HiSeq_HG004_Homogeneity-14572558/NHGRI_Illumina300X_AJtrio_novoalign_bams/HG004.GRCh38.60x.1.bam*
- *HG005 https://ftp-trace.ncbi.nlm.nih.gov/ReferenceSamples/giab/data/ChineseTrio/HG005_NA24631_son/HG005_NA24631_son_HiSeq_300x/NHGRI_Illumina300X_Chinesetrio_novoalign_bams/HG005.GRCh38_full_plus_hs38d1_analysis_set_minus_alts.300x.bam*
- *HG006 https://ftp-trace.ncbi.nlm.nih.gov/ReferenceSamples/giab/data/ChineseTrio/HG006_NA24694-huCA017E_father/NA24694_Father_HiSeq100x/NHGRI_Illumina100X_Chinesetrio_novoalign_bams/HG006.GRCh38_full_plus_hs38d1_analysis_set_minus_alts.100x.bam*
- *HG007 https://ftp-trace.ncbi.nlm.nih.gov/ReferenceSamples/giab/data/ChineseTrio/HG007_NA24695-hu38168_mother/NA24695_Mother_HiSeq100x/NHGRI_Illumina100X_Chinesetrio_novoalign_bams/HG007.GRCh38_full_plus_hs38d1_analysis_set_minus_alts.100x.bam*

ENSEMBL
https://m.ensembl.org/info/data/mysql.html

Shapefiles for UK
http://discover.ukdataservice.ac.uk/catalogue/?sn=5819&tyep=Data%20catalogue
http://census.ukdataservice.ac.uk/get-data/boundary-data.aspx
https://ukdataservice.ac.uk/help/data-types/census-data/
https://gadm.org/

Exon capture regions
http://biobank.ndph.ox.ac.uk/ukb/ukb/auxdata/xgen_plus_spikein.b38.bed

ClinVar
https://www.ncbi.nlm.nih.gov/clinvar/

UKB data showcase
https://biobank.ndph.ox.ac.uk/showcase/search.cgi

GERP
http://mendel.stanford.edu/SidowLab/downloads/gerp/hg19.GERP_scores.tar.gz

Eigen
http://www.funlda.com/toolkit

LINSIGHT
http://compgen.cshl.edu/LINSIGHT/

CADD
https://cadd.gs.washington.edu/download

Open Targets
https://genetics.opentargets.org/

AffiXcan
https://rdrr.io/bioc/AffiXcan/man/trainingCovariates.html

umap
https://github.com/tkonopka/umap

Supplementary References

78. Eggertsson, H. P. & Halldorsson, B. V. read_haps: using read haplotypes to detect same species contamination in DNA sequences. *Bioinformatics* **37**, 2215–2217 (2021).
79. Faust, G. G. & Hall, I. M. SAMBLASTER: fast duplicate marking and structural variant read extraction. *Bioinformatics* **30**, 2503–2505 (2014).
80. Li, H. *et al.* The sequence alignment/map format and SAMtools. *Bioinformatics* **25**, 2078–2079 (2009).
81. Cleary, J. G. *et al.* Comparing Variant Call Files for Performance Benchmarking of Next-Generation Sequencing Variant Calling Pipelines. doi:10.1101/023754
82. Tan, A., Abecasis, G. R. & Kang, H. M. Unified representation of genetic variants. *Bioinformatics* **31**, 2202–2204 (2015).
83. Nurk, S. *et al.* The complete sequence of a human genome. *bioRxiv* 2021.05.26.445798 (2021). doi:10.1101/2021.05.26.445798
84. McLaren, W. *et al.* Deriving the consequences of genomic variants with the Ensembl API and SNP Effect Predictor. **26**, 2069–2070 (2010).
85. Thorolfsdottir, R. B. *et al.* Coding variants in RPL3L and MYZAP increase risk of atrial fibrillation. *Commun. Biol. 2018 11* **1**, 1–9 (2018).
86. Patterson, N., Price, A. L. & Reich, D. Population Structure and Eigenanalysis. *PLOS*

Genet. **2**, e190 (2006).
87. Privé, F., Aschard, H., Ziyatdinov, A. & Blum, M. G. B. Efficient analysis of large-scale genome-wide data with two R packages: bigstatsr and bigsnpr. *Bioinformatics* **34**, 2781–2787 (2018).
88. Purcell, S. M. PLINK: a toolset for whole-genome association and population-based linkage analysis. *Am. J. Hum. Genet.* **81**, (2007).
89. Clark, D. W. *et al.* Associations of autozygosity with a broad range of human phenotypes. *Nat. Commun. 2019 101* **10**, 1–17 (2019).
90. Kunert-Graf, J., Sakhanenko, N. & Galas, D. Allele Frequency Mismatches and Apparent Mismappings in UK Biobank SNP Data. *bioRxiv* 2020.08.03.235150 (2020). doi:10.1101/2020.08.03.235150
91. Manichaikul, A. *et al.* Robust relationship inference in genome-wide association studies. *Bioinformatics* **26**, 2867–2873 (2010).
92. Pebesma, E. Simple features for R: Standardized support for spatial vector data. *R J.* **10**, 439–446 (2018).
93. Applied Spatial Data Analysis with R. *Appl. Spat. Data Anal. with R* (2008). doi:10.1007/978-0-387-78171-6
94. Gräler, B., Pebesma, E. & Heuvelink, G. Spatio-temporal interpolation using gstat. *R J.* **8**, 204–218 (2016).
