# Supplementary material for: The sequences of 150,119 genomes in the UK Biobank
Source: Nature. 2022 Jul 20;607(7920):732–40. doi: 10.1038/s41586-022-04965-x (PMC9329122; doi:10.1038/s41586-022-04965-x)
Supplement: Supplementary file 2 — Reporting Summary [file 41586_2022_4965_MOESM2_ESM.pdf]

nature portfolio

Corresponding author(s): Bjarni V. Halldorsson
Kari Stefansson

Last updated by author(s): 2022 04 29

Reporting Summary

Nature Portfolio wishes to improve the reproducibility of the work that we publish. This form provides structure for consistency and transparency in reporting. For further information on Nature Portfolio policies, see our Editorial Policies and the Editorial Policy Checklist.

Statistics

For all statistical analyses, confirm that the following items are present in the figure legend, table legend, main text, or Methods section.

n/a	Confirmed	
☐	☒	The exact sample size (*n*) for each experimental group/condition, given as a discrete number and unit of measurement
☐	☒	A statement on whether measurements were taken from distinct samples or whether the same sample was measured repeatedly
☐	☒	The statistical test(s) used AND whether they are one- or two-sided<br>*Only common tests should be described solely by name; describe more complex techniques in the Methods section.*
☐	☒	A description of all covariates tested
☐	☒	A description of any assumptions or corrections, such as tests of normality and adjustment for multiple comparisons
☐	☒	A full description of the statistical parameters including central tendency (e.g. means) or other basic estimates (e.g. regression coefficient) AND variation (e.g. standard deviation) or associated estimates of uncertainty (e.g. confidence intervals)
☐	☒	For null hypothesis testing, the test statistic (e.g. *F*, *t*, *r*) with confidence intervals, effect sizes, degrees of freedom and *P* value noted<br>*Give P values as exact values whenever suitable.*
☒	☐	For Bayesian analysis, information on the choice of priors and Markov chain Monte Carlo settings
☒	☐	For hierarchical and complex designs, identification of the appropriate level for tests and full reporting of outcomes
☐	☒	Estimates of effect sizes (e.g. Cohen's *d*, Pearson's *r*), indicating how they were calculated

Our web collection on statistics for biologists contains articles on many of the points above.

Software and code

Policy information about availability of computer code

Data collection	We used publicly available software URLs are listed below. BamQC (v 1.0.0), https://github.com/DecodeGenetics/BamQC. GraphTyper (v2.7.1), https://github.com/DecodeGenetics/graphtyper. GATK resource bundle (v4.0.12), gs://genomics-public-data/resources/broad/hg38/v0. Svimmer (v0.1), https://github.com/DecodeGenetics/svimmer. popSTR (v2.0), https://github.com/DecodeGenetics/popSTR. Dipcall (v0.1), https://github.com/lh3/dipcall. RTG Tools (v3.8.4), https://github.com/RealTimeGenomics/rtg-tools. bcl2fastq (v2.20.0.422), https://support.illumina.com/sequencing/sequencing_software/bcl2fastq-conversion-software.html. Samtools (v1.9), http://www.htslib.org/. Samblaster (v0.1.24) https://github.com/GregoryFaust/samblaster.
Data analysis	We used R (v3.6.0) https://www.r-project.org/ extensively to analyze data and create plots.

For manuscripts utilizing custom algorithms or software that are central to the research but not yet described in published literature, software must be made available to editors and reviewers. We strongly encourage code deposition in a community repository (e.g. GitHub). See the Nature Portfolio guidelines for submitting code & software for further information.

Data

Policy information about availability of data

All manuscripts must include a data availability statement. This statement should provide the following information, where applicable:

- Accession codes, unique identifiers, or web links for publicly available datasets
- A description of any restrictions on data availability
- For clinical datasets or third party data, please ensure that the statement adheres to our policy

WGS, genotype data, phased and imputed data can be accessed via the UKB research analysis platform (RAP), https://ukbiobank.dnanexus.com/landing. The

Research Analysis Platform is open to researchers who are listed as collaborators on UK Biobank-approved access applications. Summary statistics for GWAS studies are available at https://www.decode.com/summarydata/. DR score is included as supplementary data. Summary statistics for the Danish replication phenotype can be made available upon request to OBV. Summary statistics for the Icelandic replication phenotype can be made avaliable upon request to KS. Human reference genome GRCh38, http://ftp.1000genomes.ebi.ac.uk/vol1/ftp/technical/reference/GRCh38_reference_genome/. GIAB WGS samples https://ftp-trace.ncbi.nlm.nih.gov/ReferenceSamples/giab/data/ ENSEMBL https://m.ensembl.org/info/data/mysql.html.

Field-specific reporting

Please select the one below that is the best fit for your research. If you are not sure, read the appropriate sections before making your selection.

☒ Life sciences ☐ Behavioural & social sciences ☐ Ecological, evolutionary & environmental sciences

For a reference copy of the document with all sections, see nature.com/documents/nr-reporting-summary-flat.pdf

Life sciences study design

All studies must disclose on these points even when the disclosure is negative.

Sample size	The UKB has approximately 500,000 samples, a pseudo-random subset of 150,119 of those were sequenced. No statistical analysis was performed to choose sample size. The study presented here is the first data release of the WGS of all 500,000 participants, the choice of sample size was a balance between purported power in association analysis and a consideration of the time from start and finish of the project. All available samples in the Icelandic and Danish replication cohorts were used for replication analysis.
Data exclusions	A small subset of individuals withdrew consent during the time of study.
Replication	We attempted replication for SNPs when the phenotype information was available and the variant was present and common enough in a replication. We attempted replication for rare variants in menarche and uric acid in Denmark and Iceland respectively. In both cases the association was successfully replicated.
Randomization	Samples were pseudorandomly selected among the set of 500,000 samples in the UK biobank and pseudorandomly distributed between the two sequencing centers.
Blinding	Investigator were blinded to the randomization.

Reporting for specific materials, systems and methods

We require information from authors about some types of materials, experimental systems and methods used in many studies. Here, indicate whether each material, system or method listed is relevant to your study. If you are not sure if a list item applies to your research, read the appropriate section before selecting a response.

Materials & experimental systems

n/a	Involved in the study	
☒	☐	Antibodies
☒	☐	Eukaryotic cell lines
☒	☐	Palaeontology and archaeology
☒	☐	Animals and other organisms
☐	☒	Human research participants
☒	☐	Clinical data
☒	☐	Dual use research of concern

Methods

n/a	Involved in the study	
☒	☐	ChIP-seq
☒	☐	Flow cytometry
☒	☐	MRI-based neuroimaging

Human research participants

Policy information about studies involving human research participants

Population characteristics	Characteristics of the UK biobank have been described in Sudlow, C. et al. UK Biobank: An Open Access Resource for Identifying the Causes of a Wide Range of Complex Diseases of Middle and Old Age. PLOS Med. 12, e1001779 (2015).
Recruitment	Recruitment of individuals to the UK biobank has been described in previous studies, the research describes the whole genome sequencing of those samples.
Ethics oversight	The North West Research Ethics Committee reviewed and approved UKB's scientific protocol and operational procedures (REC Reference Number: 06/MRE08/65). Data for this study were obtained and research conducted under the UKB applications license numbers 24898 and 68574.

Note that full information on the approval of the study protocol must also be provided in the manuscript.
